# A coordinated transcriptional program controls de novo Golgi biogenesis

**DOI:** 10.1038/s44318-026-00828-7

**Published:** 2026-06-06

**Authors:** Francesca Forno, Domenico Abete, Elena V Polishchuk, Xabier Bujanda Cundin, Fioranna Renda, Roberta Crispino, Josephine Salzano, Raffaella Petruzzelli, Rossella De Cegli, Martina Sofia, Nicolina Cristina Sorrentino, Lorenzo Vaccaro, Davide Cacchiarelli, Juul Verbakel, Jan De Boer, Bruno Goud, Alexey Khodjakov, Franck Perez, Roman S Polishchuk

**Affiliations:** 1 Telethon Institute of Genetics and Medicine, Pozzuoli, Italy; 2 Institut Curie, Paris, France; 3 Wadsworth Center, New York State Department of Health, Albany, NY USA; 4 Armenise/Harvard Laboratory of Integrative Genomics, Pozzuoli, Italy; 5 Department of Translational Medicine, University of Naples “Federico II”, Naples, Italy; 6 Genomics and Experimental Medicine Program, Scuola Superiore Meridionale (SSM School of Advanced Studies), Naples, Italy; 7 Eindhoven University of Technology, Eindhoven, The Netherlands

**Keywords:** Organelles

## Abstract

The Golgi apparatus expands during differentiation and increased secretory demand, yet the transcriptional mechanisms governing its biogenesis remain poorly defined. To investigate this process, we developed an enzymatic approach to ablate preexisting Golgi and induce large-scale de novo organelle formation. Transcriptional profiling of cells rebuilding the Golgi revealed a coordinated induction of a broad gene network, coinciding with structural and functional maturation of the organelle. This network spans all Golgi subcompartments and activities, supporting the existence of a unified “Golgi regulon” that synchronizes expression of components required for organelle integrity. Failure to activate this regulon impaired Golgi reassembly. Promoter analysis and RNAi screening identified CREB3L1 as a central transcription factor for Golgi gene activation. Following Golgi loss, CREB3L1 translocates to the nucleus and activates its target genes, thereby driving biosynthesis of the components that are essential for organelle assembly. These findings demonstrate that Golgi biogenesis is governed by a coordinated transcriptional program that promotes organelle regeneration and enables secretory-pathway plasticity. This mechanism may support physiological remodeling by coupling Golgi biogenesis to cellular demand.

## Synopsis

When the demand for secretion is high, cells build new Golgi cisternae through the coordinated activation of a diverse gene network. This work finds CREB3L1 at the centre of this transcriptional program, enabling structural and functional maturation of newly formed Golgi stacks.

- A selective enzymatic ablation system enables synchronized de novo biogenesis of a fully functional Golgi apparatus.
- Single-cell RNA-seq reveals coordinated activation of a broad Golgi gene network: a “Golgi regulon” that spans Golgi compartments and functions.
- Golgi gene activation is required for structural maturation and trafficking competence of newly formed Golgi stacks.
- CREB3L1 acts as a key transcriptional regulator that drives Golgi gene expression and organelle regeneration after Golgi loss.

## Abstract (web summary)

CREB3L1 coordinates a Golgi regulon that governs the formation of functional Golgi stacks.

## Graphical abstract

**Figure:**
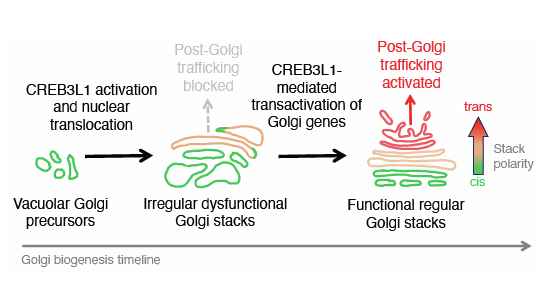

**Figure:**
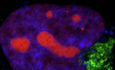

## Introduction

Biogenesis of the Golgi apparatus is a vital process that occurs in various physiological contexts, particularly during increased secretory demand and cell differentiation. Golgi expansion is observed in various secretory cell types, including pancreatic beta cells, thyroid cells, and mammary acinar cells, upon stimulation (Clermont et al, 1993; Marsh et al, 2004; García et al, 2017). In B cell differentiation, for example, the Golgi volume increases nearly sevenfold to support antibody secretion (Kirk et al, 2009). New Golgi units emerge in differentiating neurons and muscle cells (Lu et al, 2001; Valenzuela and Perez, 2015), and in organisms such as *Giardia lamblia*, Golgi-like structures appear only at specific life stages (Luján et al, 1995), underscoring the organelle dynamic and responsive nature.

Golgi biogenesis requires the synthesis of proteins, lipids and other macromolecules to support organelle growth and function. However, the transcriptional mechanisms coordinating these biosynthetic processes with Golgi morphogenesis remain incompletely understood. A major barrier to dissecting this regulation has been the absence of an experimental system capable of triggering synchronized, large-scale formation of new Golgi structures from a depleted state. Such a system would ideally initiate from near-complete Golgi loss, requiring cells to regenerate morphologically and functionally mature Golgi stacks using newly synthesized components.

Complete Golgi disassembly can be achieved by treating cells with Brefeldin A (BFA), but upon drug removal, the Golgi rapidly reassembles from preexisting components without requiring new protein or lipid synthesis (Lippincott-Schwartz et al, 1989). Because this bypasses de novo biosynthesis, BFA washout may not fully capture the requirements of de novo Golgi biogenesis.

To address this, alternative models have been explored. In our earlier work, we employed diaminobenzidine (DAB) to inactivate Golgi in mannosidase II-HRP (ManII-HRP) expressing cells and, thereby, initiate new Golgi formation. Although Golgi-like membranes appeared within 2–6 h, they failed to form stacked cisternae or support cargo trafficking (Jollivet et al, 2007). Moreover, Golgi inactivation has been shown to impair mitotic progression and promote cell death (Guizzunti and Seemann, 2016).

Another strategy—laser-mediated removal of the Golgi-containing region—elicits organelle regeneration in the remaining portion of the cell (Tängemo et al, 2011; Ronchi et al, 2014). Notably, this approach revealed that Golgi regeneration occurs slowly, with discernible stacks forming only after ~12 h. However, its technical complexity and low throughput limit its broader application.

The slow kinetics of Golgi biogenesis prompted us to revisit the enzyme-inactivation method with extended recovery time to enable full regeneration of functional Golgi. Here, we demonstrate that selective inactivation of the preexisting Golgi triggers the formation of new, morphologically and functionally mature Golgi structures in a significant fraction of cells. This system provides a unique platform to investigate de novo Golgi biogenesis. Using single-cell RNA sequencing, we identify a robust transcriptional program activated during this process, including the upregulation of a broad Golgi gene network, implicating transcriptional regulation as a critical component of Golgi biogenesis.

## Results

### Cells recover Golgi stacks after enzyme-mediated inactivation of preexisting organelle

In our previous study, we observed that incubating HeLa cells expressing ManII-HRP with H_2_O_2_ and DAB resulted in the deposition of a DAB polymer that crosslinked the lumenal contents of Golgi cisternae, thereby fully inactivating the Golgi apparatus (Jollivet et al, 2007). This inactivation of the preexisting organelle stimulated the formation of “new” Golgi-like structures in a large fraction of the cell population, providing an effective tool to trigger Golgi biogenesis. However, we also noted that these newly forming Golgi units were structurally immature and failed to support trafficking, which limited the utility of this approach to study the early stages of Golgi biogenesis (Jollivet et al, 2007).

**Figure 1:**
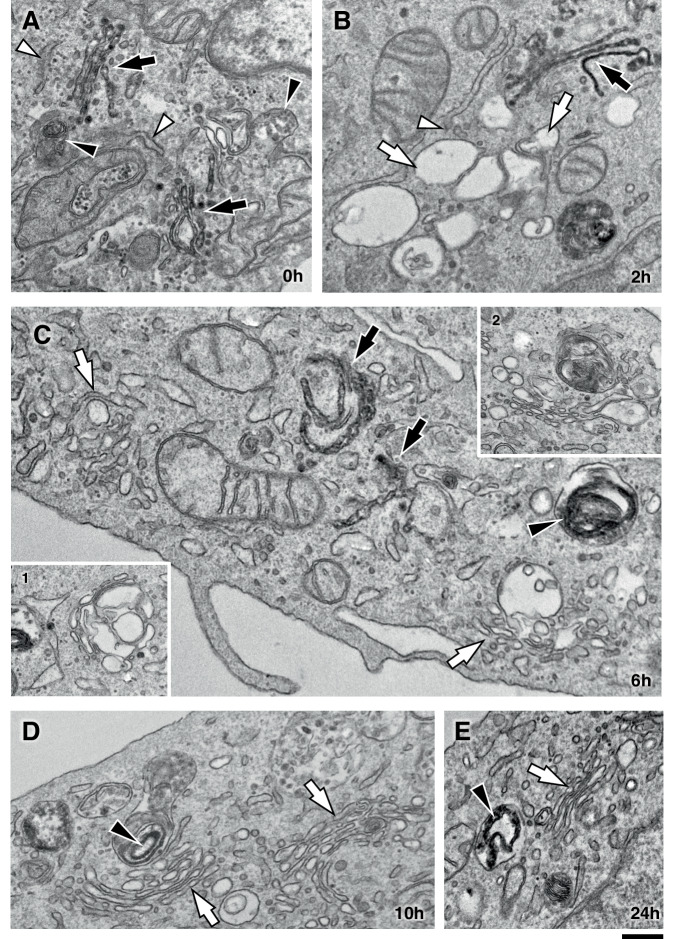
HeLa cells recover Golgi stacks after inactivation of the preexisting Golgi organelle. ManII-HRP-expressing HeLa cells were incubated with DAB and H_2_O_2_ to cross-link the preexisting (“old”) Golgi and were fixed for EM immediately (**A**) or after incubation with fresh medium for 2 (**B**), 6 (**C**), 10 (**D**), or 24 h (**E**). (**A**) Black arrows indicate Golgi stacks with electron-dense DAB polymer, which was deposited within cisternae across the entire stack. Endo-lysosomal structures (black arrowheads) and ER cisternae (white arrowheads) did not contain DAB precipitate. (**B**–**E**) DAB-negative, newly forming Golgi structures (“new” Golgi) are indicated by white arrows and were initially represented by vacuolar clusters (**B**) located near ER exit sites (white arrowhead in (**B**)), irregular stacks ((**C**) and insets 1, 2), or regular stacks (**D**, **E**). Remnants of DAB-positive old Golgi were detected in the cytosol (black arrows) or sequestered inside lysosomes (black arrowheads). Scale bar: 360 nm (**A**–**E**). Source data are available online for this figure.

To test whether fully functional Golgi might be recovered, we first optimized HRP-mediated inactivation procedure to improve cell survival (see “Methods”). Second, we extended the observation to longer recovery time (up to 24 h) that may be required to complete de novo Golgi biogenesis (Tängemo et al, 2011; Ronchi et al, 2014) and investigated the formation of new Golgi structures by electron microscopy (EM). We found that immediately after treating the cells with low dose of H_2_O_2_, precipitated DAB filled all the cisternae in most Golgi stacks (Fig. 1A; Appendix Fig. S1A). In a small subset of the stacks (less than 10%) only the cis-most or trans-most cisternae remained DAB-negative. The other intracellular structures, including the Golgi-adjacent ER, ER exit sites, endosomes, and lysosomes (Fig. 1A), were not positive for DAB. This indicates that HRP-mediated inactivation specifically targets and effectively eliminates the pre-existing Golgi, without affecting other organelles or compartments.

**Figure EV1:**
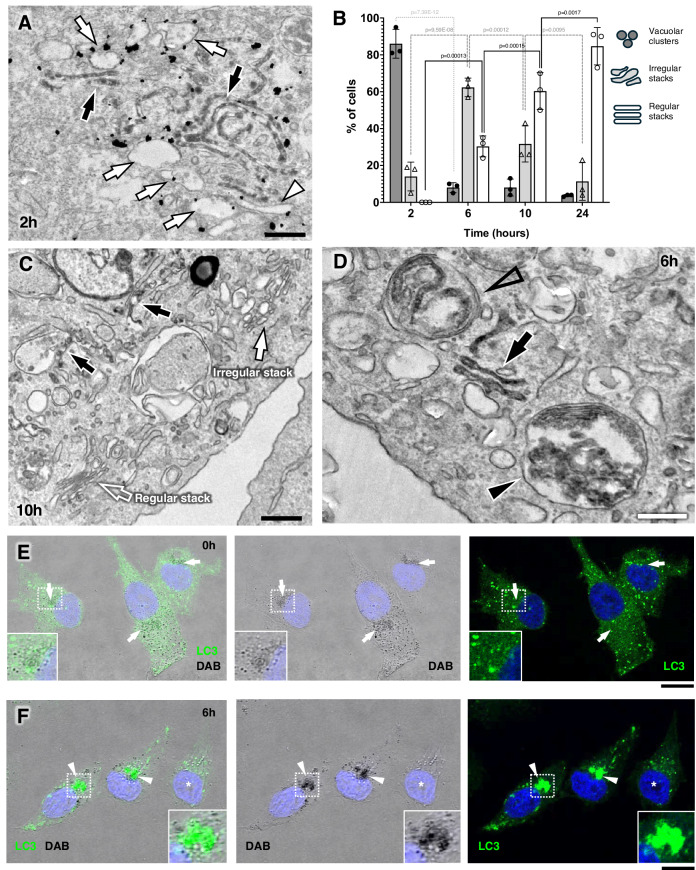
Morphodynamics of newly forming Golgi and inactivated Golgi structures in ManII-HRP-expressing cells. (**A**) Immuno-EM shows GM130-associated gold particles at the newly forming Golgi (white arrows) 2 h after inactivation of the old Golgi (black arrows). (**B**) Morphometric analysis shows cell fractions with different phenotypes of new Golgi at different time points post-inactivation (mean ± SD, two-way ANOVA; *n* = 3 replicates). (**C**) White and empty arrows show different phenotypes of the new Golgi within the same cell 10 h after inactivation of the old Golgi (black arrows). (**D**) DAB-positive remnants of the old Golgi were detected in the cytosol (arrow), in the autophagosome (empty arrowhead) and autolysosome (arrowhead). (**E**, **F**) Representative images show that inactivated DAB-positive Golgi (**E**, arrowheads) recruits LC3 by 6 h post-inactivation (**F**, arrowheads). Asterisk indicates DAB-negative cell without crosslinked Golgi. Scale bars: 320 nm (**A**, **C**, **D**), 18 µm (**E**, **F**).

Two hours after inactivation, we observed clusters of vacuolar membrane-bound structures located near the old DAB-positive Golgi remnants (Fig. 1B). Further immuno-gold labeling for GM130 confirmed that these newly forming clusters represented Golgi-like compartments (Fig. EV1A, white arrows). Frequently, the membranes within these clusters appeared stacked together (Fig. 1B, white arrows), and some began to flatten, acquiring cisternae-like shapes (Fig. EV1A, arrowhead). The ultrastructure of these membrane clusters resembled previously reported morphologies of newly forming Golgi units in the cells, whose old Golgi has been eliminated by either enzyme inactivation (Jollivet et al, 2007) or laser ablation (Tängemo et al, 2011; Ronchi et al, 2014). We also noted that these Golgi-like structures were often associated with ER exit sites (ERES), exhibiting typical ultrastructural features such as a ribosome-free surface with numerous COPII-coated buds (indicated by white arrowheads in Fig. 1B and Movie EV1) and flanking clusters of tubular–vesicular membranes (Bannykh et al, 1996; Mironov et al, 2003; Weigel et al, 2021). This suggests that formation of new Golgi units might be linked to the export of material from the ER.

At 6 h after inactivation of the old Golgi, the majority of the newly forming Golgi units were represented by stacks with markedly swollen cisternae, which we termed “irregular” stacks (or “irregular” Golgi) (Fig. 1C, white arrows; inset 1). The proportion of Golgi precursors resembling vacuolar clusters significantly decreased (Fig. EV1B), and only a few Golgi structures with the morphology of regular stacks were detected (Fig. 1C, inset 2).

By 10 h, newly formed Golgi membranes began to adopt a stacked architecture with flattened cisternae being observed in a substantial fraction of cells (Figs. 1D and EV1B). Over time, the number of stacked structures increased in cells undergoing Golgi recovery (Figs. 1E and EV1B), while vacuolar clusters and irregular stacks became less frequent, although they remained present in some cells (see quantification in Fig. EV1B).

Importantly, we observed different phenotypes of newly forming Golgi within the same cell. Figure EV1C shows the irregular stack (white arrow) coexisting with normal Golgi stack (empty arrow) within the same cell, suggesting that these phenotypes represent different stages of organelle biogenesis rather than distinct pathways of Golgi recovery.

We also observed that the old inactivated Golgi stacks underwent gradual disruption, with DAB-positive cisternae detaching from one another (Figs. 1B,C and  EV1D, black arrows). These DAB-positive cisternae were also found within autophagosomes (Fig. EV1D, empty arrowhead) and within lysosome-like structures (Figs. 1C–E and  EV1D, black arrowheads). In agreement with previous study (Gomes-da-Silva et al, 2019), we observed that, with the time significant portions of the old Golgi became decorated with the autophagic marker LC3 (Fig. EV1E,F). This is consistent with EM observations showing the sequestration of DAB-positive cisternae within autophagosomes and autolysosomes. Apart from the appearance of autophagic and lysosomal structures sequestering the old Golgi, other intracellular organelles (such as mitochondria or the ER) appeared largely normal during Golgi recovery (Fig. 1). This indicates that the impact of Golgi inactivation is relatively specific and does not significantly affect other intracellular compartments or structures.

In summary, our EM observations indicate that cells are capable of rebuilding a new Golgi organelle with stacked architecture after the inactivation of the pre-existing Golgi. However, this process requires a significant amount of time (usually ≥10 h) and can vary considerably from one cell to another, as evidenced by the presence of substantial fractions of cells with different Golgi phenotypes even at the 10- and 24-h time points (Fig. EV1B).

These observations were further confirmed by live cell imaging in ManII-HRP cells expressing the fluorescent Golgi marker GalT-GFP. We found that DAB deposition in preexisting Golgi quenched GalT-GFP fluorescence (Movie EV2). Subsequently, cells exhibited a gradual reappearance of the GalT-GFP signal within newly forming Golgi units, reaching a recovery plateau ~10–12 h post-inactivation (Movie EV3; Appendix Fig. S1A,B). Notably, the viability of cells undergoing Golgi regeneration appeared largely preserved, as many proceeded through cell division.

### Biogenesis of the new Golgi organelle is associated with changes in its molecular composition

Our data showed that cells could reconstitute new Golgi units after inactivation, but we wondered whether the molecular composition of the recovering Golgi was also restored. We analyzed the localization of a panel of Golgi markers including proteins from various Golgi compartments (*cis*, medial, *trans*) and from different functional classes of Golgi proteins (tethers, coat/adaptors, regulatory GTPases, glycosylation enzymes, structural proteins, etc.). Notably, the panel of markers included proteins with varying topologies ranging from transmembrane to peripheral Golgi proteins.

**Figure 2:**
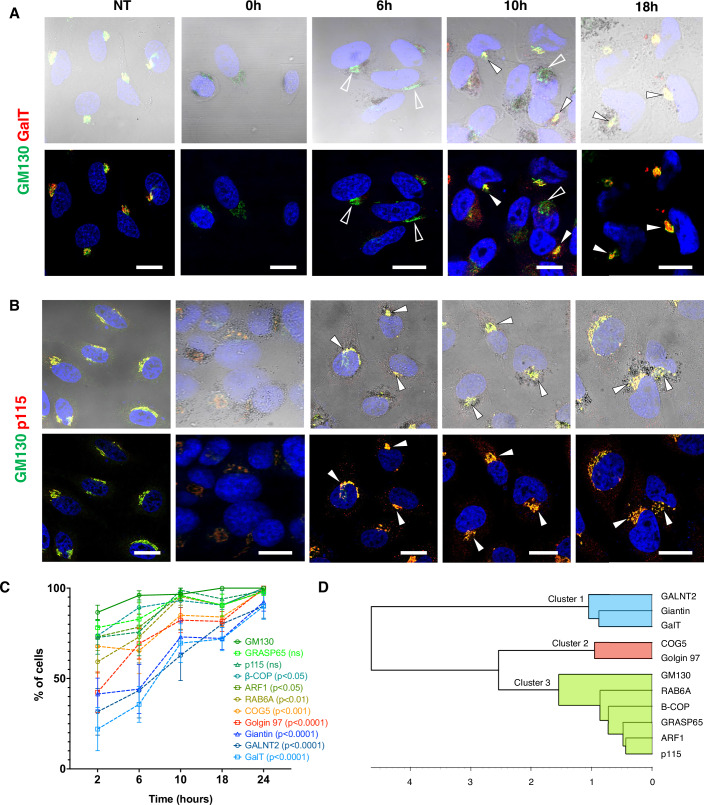
Dynamics of Golgi resident proteins in the newly forming Golgi units. ManII-HRP-expressing HeLa cells were incubated with DAB and H_2_O_2_ to cross-link the preexisting (old) Golgi and fixed for IF either immediately or after incubation with fresh medium for 2, 6, 10, or 24 h. DAB-treated and non-treated (NT) cells were stained for GM130 (green) and another Golgi marker (red), as indicated in the corresponding panels. (**A**) Arrowheads indicate newly forming Golgi that recovered GalT; empty arrowheads show newly forming Golgi lacking GalT. (**B**) Arrowheads show that almost all newly forming Golgi units contain p115. (**C**) The graph shows the percentage (mean ± SD) of cells with the respective marker in the new Golgi at each time point following inactivation of the old Golgi. *P* values indicating the statistical significance for comparison of each marker with GM130 are provided (two-way ANOVA; *n* = 3 replicates). (**D**) Cluster analysis of the time course experiments (as shown in (**C**)) demonstrates the degree of similarity in the dynamics of Golgi marker appearance within newly forming Golgi units. Scale bars: 17 µm (**A**, **B**). Source data are available online for this figure.

As previously reported by Jollivet et al (Jollivet et al, 2007), GM130 appeared rapidly at perinuclear, newly forming Golgi structures adjacent to the old DAB-positive Golgi (Fig. 2A, empty arrows). Because GM130 was also detected by EM in early-stage recovering Golgi membranes (Fig. EV1A), we used it as a reference marker to quantify the kinetics of other Golgi proteins repopulating the emerging Golgi membranes.

We found that Golgi markers were recovered in the newly forming Golgi with distinct kinetics (see examples in Fig. 2A,B and quantification in Fig. 2C). Cluster analysis identified three main marker groups based on their recovery dynamics (Fig. 2D). The first group comprised cytosolic proteins p115, GRASP65, ARF1, β-COP and RAB6A (Fig. 2D), which appeared on newly forming Golgi membranes with kinetics similar to, or slightly slower than, GM130 (Fig. 2B,C). This likely reflects their rapid recruitment from the cytosol and/or early secretory compartments, such as ER exit sites and the ER–Golgi intermediate compartment (ERGIC), where substantial pools of these proteins reside (Scales et al, 1997; Nelson et al, 1998; Marra et al, 2001). Golgi markers from second and third groups (Fig. 2D) showed slower recovery in the new Golgi (Fig. 2C). In particular, transmembrane proteins of the core Golgi region, including GalT, Giantin, and GALNT2 (cluster 1 in Fig. 2D), were detected in more than 50% of cells only by 10 h post-inactivation (Fig. 2C). Notably, this timing coincided with the appearance of regular stacks in a large fraction of cells (see EM data in Figs. 1D and  EV1B), suggesting that a substantial (or complete) set of Golgi proteins is required to establish the typical stacked-cisternal architecture. Finally, even at 24 h post-inactivation, a small subset of cells (about 10%) failed to recover a full marker set in newly forming Golgi units (Fig. 2C), indicating heterogeneity in the efficiency of the recovery process across the cell population.

### Newly formed Golgi supports trafficking of cargo proteins along different routes

We next tested whether newly forming Golgi structures were functional. To this end, we combined the analysis of Golgi biogenesis with an assessment of trafficking capacity using synchronized RUSH cargoes (Boncompain et al, 2012).

**Figure 3:**
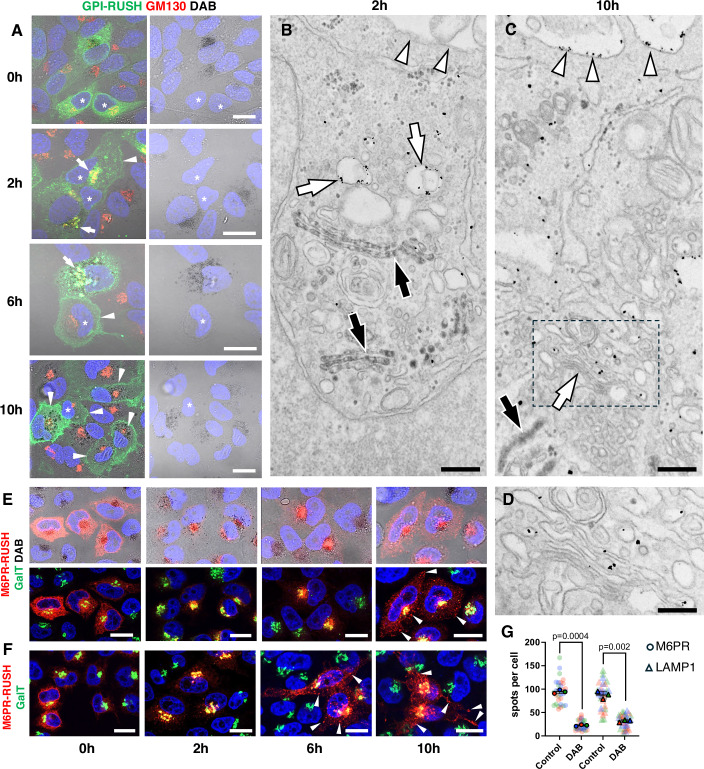
Dynamics of cargo trafficking through newly forming Golgi units. (**A**) ManII-HRP-expressing HeLa cells were transfected with GPI-GFP-RUSH, incubated with DAB and fixed immediately or after 2, 6, or 10 h incubation with biotin to activate trafficking, then labeled for GM130. Asterisks mark DAB-negative cells (internal control). GPI-RUSH reached the Golgi by 2 h in both DAB-positive and DAB-negative cells (arrows). In DAB-positive cells, GPI-RUSH was retained in newly forming Golgi at 6 h (arrows) and reached the plasma membrane (PM) mainly by 10 h (arrowheads). In DAB-negative cells, GPI-RUSH was efficiently delivered to the PM at 2–10 h (arrowheads) and cleared from the Golgi by 6 h. (**B**–**D**) Immuno-gold EM (anti-GFP) after Golgi crosslinking as in (**A**). At 2 h, GPI-RUSH localized to newly forming Golgi vacuolar membranes (**B**, white arrows) with no PM signal (**B**, arrowheads). At 10 h, GPI-RUSH was detected at the PM (**C**, arrowheads) and in the newly formed Golgi stacks (**C**, white arrow; D corresponds to dashed box). No GPI-RUSH was detected in the old Golgi (**B**, **C**; black arrows). (**E**, **F**) M6PR-mCherry-RUSH trafficking was activated in ManII-HRP cells with (**E**) or without (**F**) Golgi crosslinking. M6PR-RUSH reached newly forming Golgi but was retained there until 6 h post-inactivation (**E**). In control cells, M6PR-RUSH was efficiently delivered to post-Golgi compartments by 6 h (**F**; arrowheads). In DAB-treated cells, post-Golgi delivery was mainly observed by 10 h after inactivation (**E**; arrowheads). (**G**) Quantification of post-Golgi spots containing M6PR-RUSH or LAMP1-RUSH at 6 h postinactivation (average ± SD, *t* test; *n* = 3 replicates). Scale bars: 16 µm (**A**, **E**, **F**), 340 nm (**B**, **C**), 200 nm (**D**). Source data are available online for this figure.

**Figure EV2:**
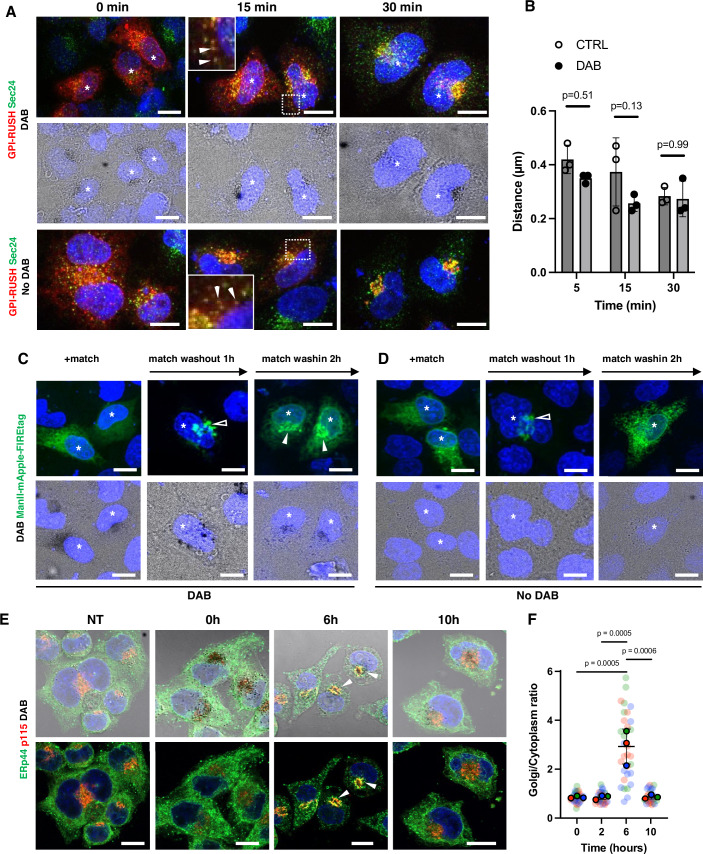
Trafficking at the ER/Golgi interface after Golgi inactivation in ManII-HRP-expressing HeLa cells. (**A**) GPI-RUSH trafficking was activated by biotin in Golgi-inactivated (DAB) and control (No DAB) cells, with GPI-RUSH localizing to Sec24-positive spots (arrowheads). Asterisks indicate GPI-RUSH-expressing cells. (**B**) Coincidence between GPI-RUSH and Sec24 spots remained similar in both conditions (mean ± SD, two-way ANOVA, *n* = 3 replicates). (**C**, **D**) ManII-HRP cells expressing FIREMate-KDEL and ManII-mApple-FIREtag (asterisks) were treated with match to block ER exit of ManII-mApple-FIREtag reporter (see “Methods”). After Golgi inactivation, match was removed in DAB-treated and control cells to allow Golgi delivery (**C**, **D**, empty arrowheads) and then re-added to capture recycling to the ER (**D**). ManII-mApple-FIREtag was retained in the newly forming Golgi (**C**, arrowheads) but efficiently recycled to the ER in controls. (**E**) ERp44 showed ER-like distribution but accumulated in the Golgi 6 h post-inactivation. (**F**) Quantification confirmed significant Golgi retention of ERp44 at 6 h (mean ± SD; one-way ANOVA; *n *= 3 replicates). Scale bars: 16 µm (**A**, **C**–**E**).

We first used ManII-HRP cells transfected with a GPI-GFP RUSH construct to monitor trafficking from the ER along the secretory route to the cell surface. After transfection, cells were incubated with DAB and H_2_O_2_ to inactivate the Golgi apparatus. Cells were then washed and immediately treated with biotin to release GPI-GFP from the ER. As expected, right after inactivation, GPI-GFP localized to the ER (Fig. 3A). Two hours later, in cells with an inactivated Golgi, most GPI-GFP was detected in newly formed GM130-positive compartments (Fig. 3A), indicating that these developing Golgi-like structures can receive cargo from the ER. This was further supported by immuno-EM, which revealed GPI-GFP within vacuolar membranes of newly forming Golgi structures (Fig. 3B) adjacent to the old DAB-positive Golgi. More detailed analysis showed that cargo exit from the ER was not affected by Golgi inactivation. Indeed, as in control cells, GPI-GFP was rapidly sorted at ERES into transport carriers, which then delivered it to newly forming Golgi units (Movies EV4 and EV5 and Fig. EV2A,B).

However, at 2 h post-inactivation, we did not detect GPI-GFP at the plasma membrane (PM) of cells regenerating their Golgi by either light or electron microscopy (Fig. 3A,B). In contrast, neighboring DAB-negative cells, whose Golgi remained intact due to low ManII-HRP expression, showed a strong GPI-GFP signal at the cell surface (Fig. 3A, arrowheads). This suggests that newly forming Golgi units are not yet mature enough to support post-Golgi trafficking. At 6 h post-inactivation, the new Golgi still retained substantial amounts of GPI-GFP (Fig. 3A), and only by 10 h cells delivered most GPI-GFP to the cell surface (Fig. 3A,C). The onset of efficient post-Golgi trafficking coincided with the appearance of regular stacks in the new Golgi (Fig. 3C,D), indicating that functional and structural maturation occur on a similar timescale. Live-cell imaging confirmed a marked delay in post-Golgi GPI-GFP trafficking in cells recovering the Golgi after inactivation (compare Movies EV6 and EV7).

A similar delay in cargo export was observed for proteins destined for endo-lysosomal compartments. In control cells, mCherry-tagged M6PR or Lamp1 appeared in post-Golgi endo-lysosomal structures as early as 2 h after release from the ER, whereas in Golgi-inactivated cells the number of such post-Golgi spots remained low until 10 h post-inactivation because cargoes were retained within newly forming Golgi units (Fig. 3E–G). This trend was further supported by live-cell imaging, which showed a significant delay in M6PR exit from newly forming Golgi structures (compare Movies EV8 and EV9).

Finally, using the CATCHFIRE approach that allows real-time monitoring of anterograde and retrograde transport (Bottone et al, 2023), we found that retrograde trafficking from newly forming Golgi to the ER was slowed down (Fig. EV2C,D). Accordingly, the ER-resident protein ERp44 was found accumulated in the Golgi area up to 6 h after Golgi inactivation (Fig. EV2E,F). This accumulation was no longer detectable at 10 h, indicating that Golgi-to-ER retrieval had also been reactivated (Fig. EV2E,F).

Taken together, these trafficking experiments indicate that the developing Golgi requires substantial time to acquire the functional capacity to export cargoes in both anterograde and retrograde directions. Notably, acquisition of this capacity correlates temporally with the appearance of regular Golgi stacks and recovery of a complete set of Golgi markers.

### De novo Golgi biogenesis requires transcription

Once we established that cells can rebuild a new functional Golgi, we sought to investigate whether and how they remodel their transcriptome to support de novo Golgi biogenesis.

**Figure 4:**
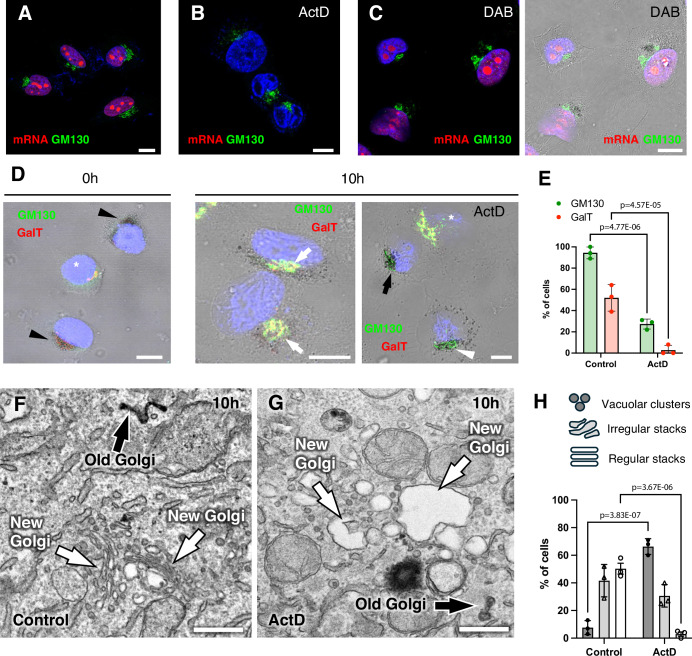
Transcription inhibition impairs Golgi biogenesis in ManII-HRP cells. (**A**) Cells were labelled with kit showing newly synthesised mRNA (red) and GM130 (green). (**B**) After 1 µg/ml Actinomycin D (ActD) treatment, no new mRNA was detected. (**C**) DAB inactivation of old Golgi followed by 6 h recovery did not affect mRNA synthesis. (**D**) Golgi recovery post-DAB was assessed with or without ActD; partial recovery (GM130 only) occurred with ActD (white arrowheads), while full recovery (GM130 and GalT) occurred without ActD (white arrows). Asterisks mark cells with low ManII-HRP expression where Golgi was not inactivated; black arrows indicate cells lacking new GM130-positive Golgi. (**E**) Percentage of cells with GM130 or GalT in new Golgi at 10 h post-inactivation, showing significant reduction with ActD (mean ± SD, two-way ANOVA; *n* = 3). (**F**–**H**) EM analysis at 10 h post-inactivation reveals disrupted Golgi organization with ActD treatment (**G**) compared to control (**F**). Golgi phenotype quantification (**H**) confirms increase in cell fraction containing vacuolar clusters instead of the regular Golgi stacks (mean ± SD, two-way ANOVA; *n* = 3 replicates). Scale bars: 8.5 µm (**A**–**D**), 420 nm (**F**, **G**). Source data are available online for this figure.

Specifically, we first aimed to determine whether transcription is required for the reconstitution of a novel and functional Golgi complex. To address this, we identified the lowest concentration (1 µg/ml) of actinomycin D (ActD) that effectively inhibits transcription while maintaining the cell viability for at least 10 h - the minimum time needed for Golgi recovery (Fig. 4A–C). ActD treatment resulted in a substantial decrease in the number of cells in which the newly forming Golgi was detectable after inactivation of the pre-existing one (Fig. 4D,E). Furthermore, although Golgi units in ActD-treated cells were positive for GM130, most of them lacked GalT, indicating structural immaturity (Fig. 4D,E). This was further mirrored by a marked suppression of GalT-GFP signal recovery in ActD-treated cells observed in vivo (Appendix Fig. S1C,D; Movie EV10). At the EM level, majority of ActD-treated cells failed to regenerate normal Golgi stacks, with most Golgi elements reaching only an incomplete stage of morphogenesis (Fig. 4F–H). These findings suggest that active transcription is essential for the completion of Golgi biogenesis.

This conclusion was further supported by experiments involving cell enucleation, which allowed us to remove the nucleus and generate cytoplasts—nucleus-free cells in which transcription does not occur. While many cytoplasts survived for 8–10 h (a period sufficient for regeneration of Golgi-like structures in control cells), they failed to rebuild a new Golgi after inactivation of the pre-existing organelle (Appendix Fig. S1E,F; Movie EV11).

### Single cell RNA-seq reveals transcriptional activation of Golgi genes in cells rebuilding the new Golgi

To determine how transcription contributes to Golgi biogenesis, we investigated changes in the transcriptional landscape of cells during this process. As outlined above, the significant heterogeneity in the kinetics and efficiency of Golgi recovery required the use of single-cell RNA sequencing (scRNA-seq), which allows transcriptome of individual cells to be analyzed during Golgi regeneration process.

ManII-HRP HeLa cells were subjected to Golgi inactivation and then collected for scRNA-seq immediately (0 h timepoint) or after incubation with fresh medium for 2, 6, 10, 14, 18 and 24 h to allow detailed analysis of transcriptome during different stages of biogenesis of the new Golgi. In parallel, the cells from each time point were subjected to IF and EM analyses to investigate how the dynamics of transcriptional responses correlated with Golgi rebuilding in the framework of the same experiment.

**Figure 5:**
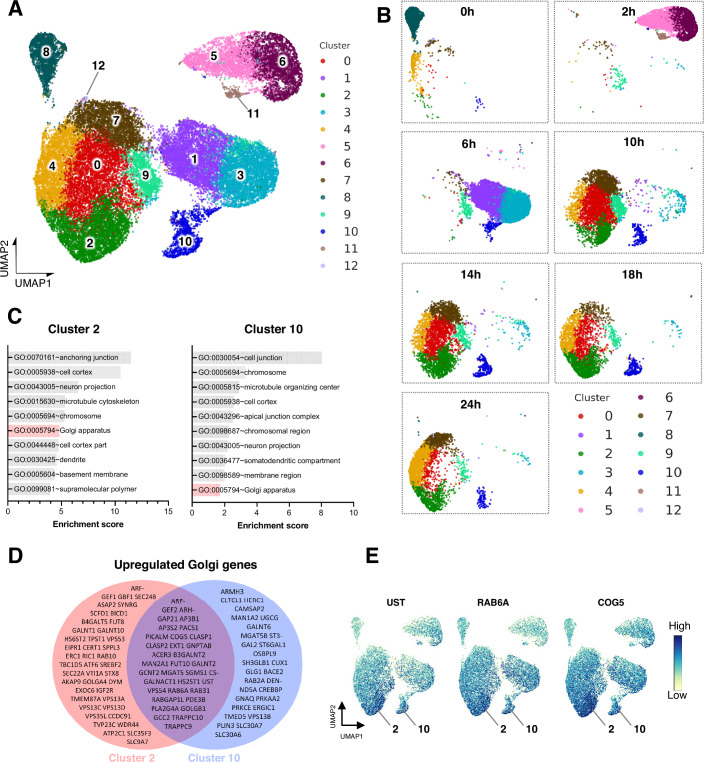
Single cell transcriptomics reveals upregulation of Golgi genes after inactivation of preexisting Golgi organelle. ManII-HRP-expressing HeLa cells were incubated with DAB and H_2_O_2_ to cross-link the preexisting Golgi and collected for scRNAseq immediately or after additional incubation with fresh medium for 2, 6, 10, 14, 18 and 24 h. (**A**) UMAP plot shows outcome of the scRNAseq analysis where dots represent individual cells with at least one count for ManII-HRP transcript. The cells in UMAP were collected from all time points and clustered based on similarity of their transcriptomes. (**B**) The panel shows UMAP plots for each time point. (**C**) Differently expressed genes in each cluster were subjected to gene ontology (GO) enrichment analysis. The graphs show top 10 enriched annotation terms among upregulated transcripts in clusters 2 and 10, including “Golgi apparatus” (highlighted in red). (**D**) VENN diagram shows upregulated Golgi genes that belong to cluster 2, cluster 10, or both. (**E**) Expression patterns of Golgi genes *UST*, *RAB6* and *COG5* are shown in UMAP plots. Cells with higher expression of these genes (dark blue) are concentrated in areas corresponding to clusters 2 and 10 shown in (**A**). Source data are available online for this figure.

The cells from different time points were prepared for sequencing (see “Methods”) and then pulled together for further NGS analysis. ManII-HRP transcripts were detected in approximately 35,000 cells and transcriptomes of individual cells were clustered based on their similarity (Fig. 5A). This analysis revealed that few cell clusters prevailed at early time points post-inactivation (Fig. 5B). Starting from 10 h, several cell clusters persisted until 24 h, indicating that they might be associated with specific cell states/fates after inactivation of the Golgi apparatus (Fig. 5B). To understand the specific features of these clusters, gene ontology (GO) enrichments analysis for up- and downregulated genes was performed for each cluster (Fig. 5C; Appendix Fig. S2). This analysis revealed that upregulated transcripts in clusters 2 and 10 were enriched in Golgi-associated genes, hereafter referred to as “*Golgi genes*” (Fig. 5C). Notably, we also found that clusters 2 and 10 shared a significant number of transactivated *Golgi genes* (Fig. 5D). In total, more than 100 upregulated *Golgi genes* were found in these clusters (Dataset EV1). This number represents a very substantial cohort considering that the sensitivity of scRNA-seq is relatively low as it usually detects about 3000 most abundant transcripts (Luecken and Theis, 2019). Further analysis of individual Golgi genes (*UST*, *RAB6A* and *COG5*) in UMAP plots indicated that their higher expression was predominantly associated with cellular clusters 2 and 10 (Fig. 5E), thereby corroborating the GO analysis conclusions regarding the upregulation of *Golgi genes* in these clusters.

**Figure 6:**
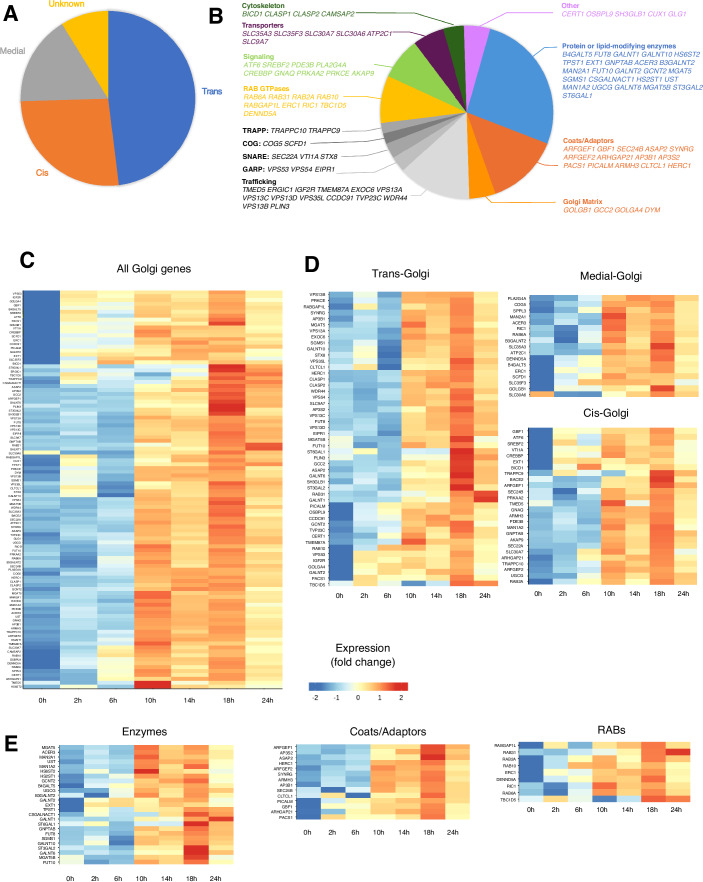
Specific features and expression dynamics of upregulated Golgi genes. (**A**, **B**) Pie plots show proportion of the upregulated Golgi genes, whose protein products are associated with specific Golgi compartment (**A**) or function (**B**). (**C**) Heatmap shows the dynamics of Golgi gene expression after inactivation of the preexisting Golgi organelle. (**D**, **E**) Heatmaps indicate that the trend for elevated expression starting from 10 h post-inactivation is common for majority of the Golgi genes, whose protein products are associated with specific Golgi compartment (**D**) or function (**E**). Source data are available online for this figure.

Next, we analyzed features of transactivated *Golgi genes* in terms of compartmentalization and function of their protein products. First, we wanted to understand whether specific compartment *cis-*, medial- or *trans*-Golgi prevails among activated genes. Although we found that genes encoding *trans*-Golgi/TGN-associated proteins were more numerous, *cis* and medial *Golgi genes* were also well represented (Fig. 6A), indicating that the activation of the *Golgi genes* in response to Golgi loss is not compartment specific. Second, we analyzed to which functional group do upregulated *Golgi genes* belong. Figure 6B shows that genes regulating different Golgi functions were represented among activated transcripts. This includes relatively large groups of genes encoding glycosylation enzymes, structural proteins (coats, adaptors and golgins) and proteins regulating membrane trafficking (components of TRAPP, COG, GARP complexes and SNARES). We also found substantial cohort of genes associated with RAB GTPase activities including *RAB6A* (Fig. 6B), whose protein products RAB6A and RAB6A’ regulate numerous Golgi functions (Goud et al, 2018). Other less prominent functional groups of upregulated *Golgi genes* contained genes encoding transporters and cytoskeleton proteins. The group of transporter genes included sugar transporters SLC35A3 and SLC35F3 as well as manganese/zinc transporters SLC30A6, SLC30A7 and ATP2C1 (Fig. 6B), which supply sugar substrates and metal cofactor for glycosylation enzymes residing in the Golgi (Foulquier and Legrand, 2020). Cytoskeleton-associated genes comprised CLASP1 and CLASP2 (Fig. 6B), whose protein products drive microtubule nucleation at the Golgi (Efimov et al, 2007). In summary, this analysis suggests activated *Golgi genes* were not restricted to a single Golgi compartment or a functional group but targeted more generally Golgi dynamics and function.

**Figure EV3:**
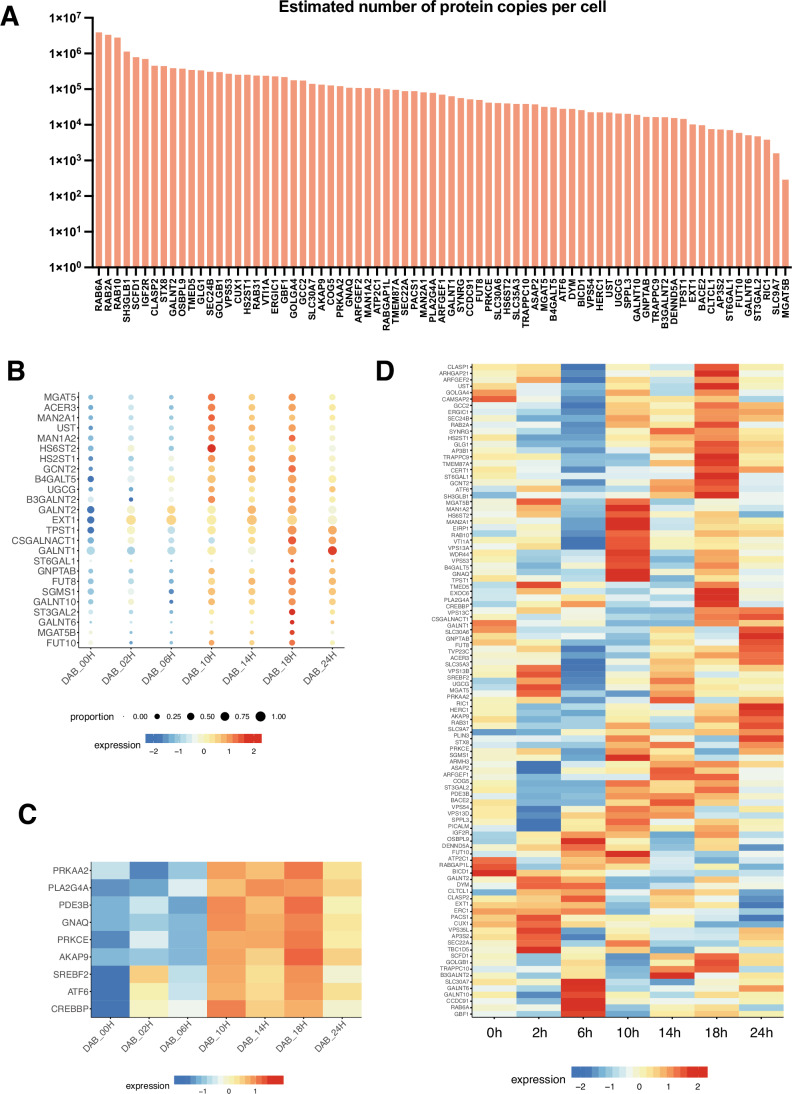
Specific features of activated Golgi genes. (**A**) The estimated numbers of copies of Golgi proteins per cell were recovered from published proteomics data (Fasimoye et al, 2023) and plotted for corresponding genes, whose activation was observed during biogenesis of the new Golgi. (**B**) The bubble plot shows a subset of the Golgi genes (encoding glycosylation enzymes). Both the expression levels and number of cells expressing these genes increase with after inactivation of the old Golgi, especially at 10 h timepoint. (**C**) The heatmap shows expression dynamics of genes encoding Golgi-associated signaling proteins. Transcription factor associated genes ATF6, CREBBP, and SREBF2 exhibit earlier transactivation and are clustered together. (**D**) The expression of the Golgi genes (same as in Fig. 6C) was evaluated at different time points after incubation with DAB in cells without detectable ManII-HRP expression (83,000 cells in total). Majority of the genes exhibit fluctuating expression without specific activation time point.

We also analyzed whether the abundance of a given protein is correlated to activation of its gene after induction of Golgi biogenesis. To this end, we mined recently published proteomics data, which provide approximate number of copies per cell for each Golgi protein (Fasimoye et al, 2023). We found that upregulated *Golgi genes* corresponded to proteins ranging from highly to poorly expressed (Fig. EV3A). These comprise *RAB6A*, whose protein product belongs to the top 20 most expressed Golgi proteins, and *GALNT2* encoding a most abundant Golgi glycosylation enzyme (Fasimoye et al, 2023). On the other hand, weakly expressed genes (e.g., *FUT10*, *GALNT6* and *MGAT5B*) were also transactivated indicating that their protein products might be needed for newly forming Golgi.

### Transactivation of Golgi genes during Golgi biogenesis happens in a synchronous manner

Our scRNA-seq data indicate that extensive Golgi biogenesis after the loss of the preexisting organelle is associated with the activation of a broad set of Golgi-related genes belonging to different functional groups and localized to distinct Golgi compartments. To further characterize this transcriptional response, we examined whether the kinetics of gene activation differ among these groups, such as whether genes associated with the *cis*-Golgi are induced earlier than those related to the *trans*-Golgi, or whether structural components are activated before glycosylation enzymes for example.

Figure 6C illustrates the temporal changes in Golgi gene expression following inactivation of the Golgi apparatus. Notably, the majority of *Golgi genes* showed a significant increase in mRNA levels and in the number of cells expressing these genes, starting between 6 and 10 h after incubation with DAB (Figs. 6C and EV3B). This elevated expression persisted until 24 h, with a peak for many genes at 18 h. This suggests that a broad activation of *Golgi genes* occurs between 6 and 10 h after the induction of Golgi biogenesis.

Interestingly, a small subset of genes displayed increased transcript levels at earlier time points (as early as 2 h post-inactivation). This group includes transcriptional regulators such as ATF6, CREBBP, and SREBF2 (Fig. EV3C), whose activation may be required to support Golgi biogenesis.

We also investigated whether the activation kinetics of relevant genes differs between Golgi compartments. However, genes associated with the *cis*, medial, and *trans*-Golgi exhibited a similar trend, showing significant activation at 10 h after the induction of Golgi biogenesis (Fig. 6D). This pattern was also observed across different functional groups of *Golgi genes*, including those encoding glycosylation enzymes, coat/adaptor-related proteins, RAB GTPases and RAB-associated proteins, and signaling factors (Figs. 6E and EV3C).

Importantly, this kinetics of *Golgi gene* activation were not observed in cells lacking detectable ManII-HRP expression (Fig. EV3D). This indicates that the Golgi-specific transcriptional response occurred only in cells where the Golgi was inactivated.

### Kinetics of Golgi gene activation correlates with structural maturation of the newly forming Golgi

Our findings suggest that structural and functional transformation of the developing Golgi coincides with the period during which scRNA-seq reveals massive activation of *Golgi genes* (~10 h after DAB crosslinking). To further confirm this correlation, we examined Golgi morphology in cells prepared for immunofluorescence and EM, in parallel with cells analyzed by scRNA-seq. New Golgi units in these cells began to acquire a regular stacked morphology by 10 h after the elimination of the preexisting Golgi, and by 14 h, substantial number of newly formed Golgi structures were represented by stacks (Appendix Fig. S3A–D). Morphogenesis of the Golgi stacks correlated with the appearance of GALNT2 in the emerging Golgi structures (Appendix Fig. S3E,F), indicating that these structures recover proteins necessary for their functions, such as glycosylation. This confirms that the activation of *Golgi genes* coincides with the structural and functional maturation of the newly forming Golgi organelle.

We then attempted to directly demonstrate that Golgi gene activation occurs in cells rebuilding the Golgi. This was challenging to monitor using standard bulk methods like real-time PCR, as the cell population following DAB treatment was highly heterogeneous in terms of Golgi recovery efficiency and kinetics. To circumvent this problem, we employed fluorescence in situ hybridization (FISH) to visualize mRNA levels of *Golgi genes* in individual cells.

We used ManII-HRP cells expressing GalT-GFP, which allowed us to observe both the DAB-positive old Golgi and the new Golgi marked by GalT-GFP. The cells were subjected to Golgi inactivation and prepared for FISH either immediately or 18 h after inactivation. The 18-h time point was chosen because most *Golgi genes* reach peak expression at this time following inactivation of the old organelle (Fig. 6C). To assess Golgi gene expression, RNA-scope FISH probes were selected for genes whose protein products belong to different functional groups, including *UST* and *GALNT2* (glycosylation enzymes), *GOLGB1* (Golgi matrix protein), and *COG5* (membrane tethering protein). These genes were part of the cohort of activated *Golgi genes* identified in the scRNA-seq dataset (Fig. 6B,C).

**Figure 7:**
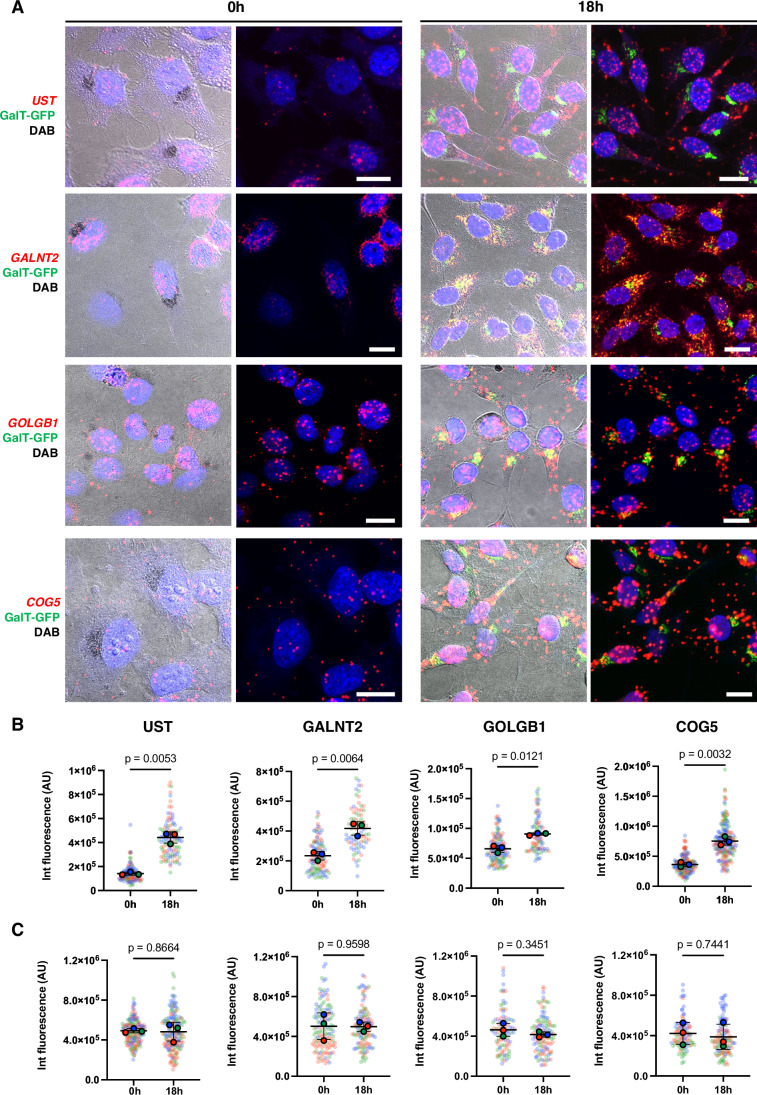
Fluorescence in situ hybridization (FISH) reveals transcriptional activation of Golgi genes in the cells building the new Golgi. HeLa cells expressing ManII-HRP and GalT-GFP were incubated with DAB and H_2_O_2_ to cross-link the preexisting (old) Golgi and fixed immediately or after incubation with fresh medium for 18 h. The cells were then subjected to FISH with RNAscope probes (see “Methods”) to reveal mRNA of *UST*, *GALNT2*, *GOLGB1* and *COG5*. mRNA-associated signal for each gene is represented by red fluorescent spots. Images in (**A**) (0 h) show that deposition of DAB polymer in the Golgi quenched GalT-GFP fluorescence, which was recovered in the new Golgi by 18 h. The cells recovering the Golgi at 18 h show significant increase in red fluorescent signal associated with mRNA of the Golgi genes, as also confirmed by fluorescence quantification (mean ± SD; *t* test, *n* = 3 replicates) in (**B**). (**C**) shows no difference in FISH fluorescent signal (mean ± SD; *t* test, *n* = 3 replicates) for the same genes in control cells, which were treated with only H_2_O_2_ and not with DAB. Scale bars: 17 µm (**A**). Source data are available online for this figure.

As shown in Fig. 7A, the GalT-GFP signal was quenched by DAB in the Golgi immediately after inactivation, while only a few red fluorescent spots associated with *UST* transcripts were present in the cells. By 18 h post-inactivation, the fluorescent signal associated with *UST* mRNA had significantly increased in cells recovering the Golgi (Fig. 7A,B). Similar increases in mRNA levels were also observed for *GALNT2*, *GOLGB1* and *COG5* (Fig. 7A,B), indicating that their transcriptional activation occurs during the biogenesis of the new Golgi. Further control experiments demonstrated that activation of Golgi genes was specifically associated with Golgi biogenesis as incubation with H_2_O_2_ alone fails to stimulate their transcription (Fig. 7C).

**Figure EV4:**
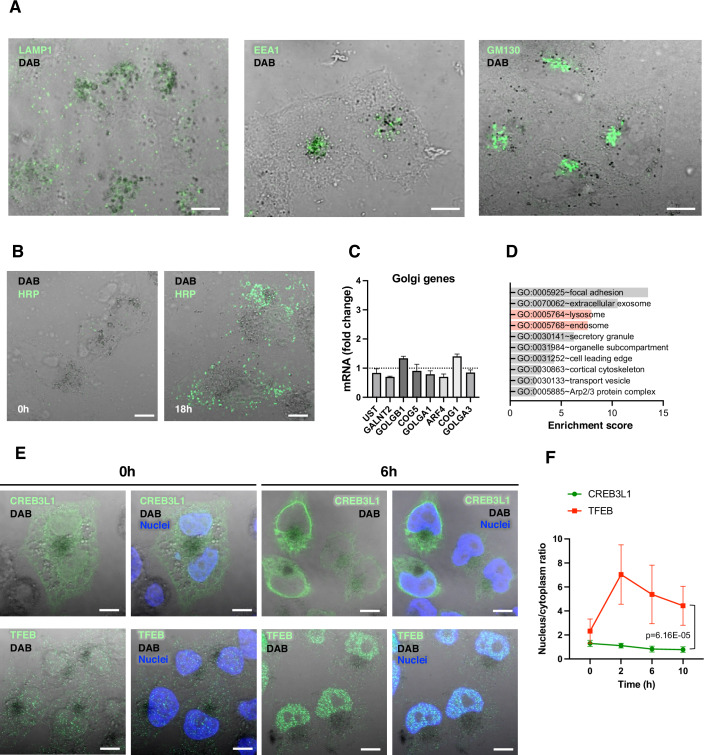
Endosome inactivation does not induce Golgi gene expression. (**A**) HeLa cells were incubated with HRP for 30 min. After HRP loading, endosomes were inactivated using DAB and H₂O₂; DAB colocalized with LAMP1 and EEA1 but not with GM130, indicating the Golgi apparatus was unaffected. (**B**) HRP uptake was minimal immediately after endosome inactivation (0 h) but was restored by 18 h, demonstrating functional and structural recovery of endosomes. (**C**) qRT-PCR analysis showed no significant changes in Golgi gene expression at 18 h post-inactivation compared to 0 h, despite endosome regeneration (mean ± SD; *t* test; *n* = 3 replicates). (**D**) Transcriptional profiles of cells subjected to endosome inactivation (treated with DAB and H₂O₂) and control cells (treated with H₂O₂ only) were compared by RNA-seq. The graph displays the top 10 GO enriched terms for genes upregulated in endosome-inactivated cells, with “Lysosome” and “Endosome” terms (highlighted in red) present but not Golgi-related terms. (**E**) Images show no nuclear translocation of CREB3L1 after endosome inactivation, whereas clear nuclear translocation of the lysosome-specific transcription factor TFEB was observed. (**F**) Quantification of the nuclear/cytoplasmic ratio shows no increase for CREB3L1 and a strong increase for TFEB after endosome inactivation (mean ± SD, two-way ANOVA; *n* = 3 replicates). Scale bars: 10 µm (**A**, **B**, **E**).

Additionally, we investigated whether the induction of *Golgi genes* was specific to Golgi biogenesis or could be triggered by the inactivation and subsequent regeneration of other organelles. To test this, we inactivated endosomes in HeLa cells using HRP uptake (Fig. EV4A), thereby inducing the reformation of new functional endocytic structures (Fig. EV4B). However, endosome inactivation and subsequent biogenesis did not accelerate the expression of a selected set of *Golgi genes* (Fig. EV4C), whose upregulation had been documented during Golgi biogenesis. We reasoned that a limited panel of Golgi genes might overlook activation of other genes, so we performed RNA-seq on cells subjected to endosome inactivation. Subsequent GO enrichment analysis confirmed the absence of enrichment for specific Golgi GO terms among upregulated transcripts (Fig. EV4D) following endosome inactivation. The only partially Golgi-related enriched terms with significant enrichment scores were “transport vesicle” (ranked 9, see Fig. EV4D) which included only five Golgi-relevant genes (*COPB2, COPA, COPB1, CLTA, and SEC31A*). This number is very small compared with the more than 100 Golgi genes that were upregulated after Golgi inactivation. In contrast, cells with inactivated endosomes showed enrichment of “endosome” and “lysosome” GO terms among the upregulated transcripts (Fig. EV4D; Dataset EV2), indicating activation of a distinct transcriptional program supporting the biogenesis of endo-lysosomal organelles.

### Induction of Golgi genes during biogenesis requires activity of transcription factor CREB3L1

The massive and concerted induction of *Golgi genes* suggested that a biogenesis program was activated under the control of one or a few master genes. We thus hypothesized that key transcription factor(s) (TFs) may be responsible for activating a set of *Golgi genes* and necessary for building the new Golgi.

To identify candidate TFs, we first used the pySCENIC tool (Aibar et al, 2017) to infer TF activity from the scRNA-seq data. Then we determined which of predicted TFs might directly regulate *Golgi genes*, whose upregulated transcripts were enriched in clusters 2 and 10. To this end, we used the FIMO tool (Grant et al, 2011) to scan promoter regions of these genes for corresponding TF binding motifs. We additionally included TFs from the CREB3 family in FIMO analysis because (i) these TFs have been reported to be regulators of secretory pathway-associated genes (Fox et al, 2010; García et al, 2017); and (ii) our set of upregulated *Golgi genes* included CREBBP, a known transcriptional coactivator of CREB3 proteins (Chung et al, 2017) (Fig. EV3C).

**Figure 8:**
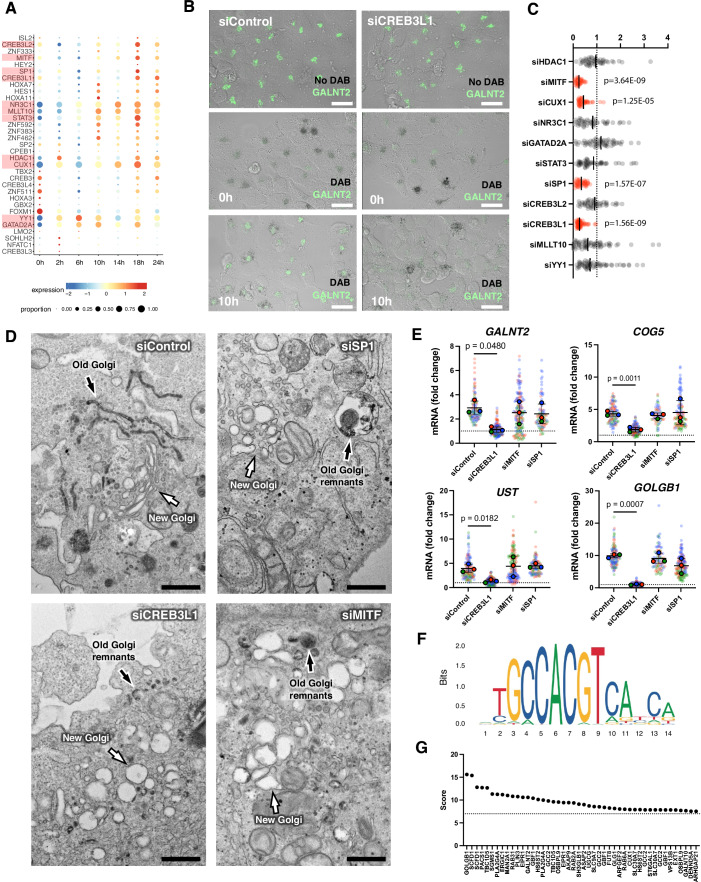
Screening for transcription factors regulating activation of Golgi genes during Golgi biogenesis. (**A**) Bubble plot showing the expression patterns of predicted transcription factors (TFs) in a scRNA-seq dataset. TF candidates selected for RNAi-based screening are highlighted in pink. (**B**, **C**) Candidate TFs were silenced in HeLa cells expressing ManII-HRP. These cells were then subjected to DAB-mediated Golgi inactivation, and the efficiency of Golgi regeneration was assessed by monitoring the recovery of the GALNT2 signal. (**B**) Representative images of cells treated with scrambled siRNAs (siControl) or CREB3L1-specific siRNA (siCREB3L1) before inactivation (No DAB), immediately after inactivation (0 h), and 10 h post-inactivation. Efficient GALNT2 recovery is observed in control cells, while CREB3L1-silenced cells show impaired recovery. (**C**) Quantification of GALNT2 signal recovery following TF silencing. Values are normalized to siControl (dashed line). siRNAs that significantly reduced GALNT2 recovery are shown in red (mean ± SD, one-way ANOVA; *n* ≥ 40 cells). (**D**) Electron microscopy images showing that control cells reestablish Golgi stacks 10 h after inactivation, whereas cells silenced for SP1, CREB3L1, or MITF fail to do so. (**E**) Expression of GALNT2, COG5, UST, and GOLGB1 was assessed by FISH immediately after Golgi inactivation and again 18 h later. mRNA fold changes between these time points were calculated based on FISH fluorescence intensity and show an increase for all tested genes in control cells (siControl). The graphs depict the impact of transcription factor silencing on this increase for each gene (mean ± SD, one-way ANOVA; *n* = 3 replicates). The dashed line indicates gene expression levels immediately after inactivation. (**F**) Position weight matrix (PWM) of the CREB3L1 binding motif used for scanning promoter regions of activated Golgi genes. (**G**) Motif scores for predicted CREB3L1 binding sites in promoter regions of activated Golgi genes. Higher scores indicate stronger motif matches. The dashed line marks the threshold score of 7, below which binding probability was considered weak. All shown matches were statistically significant (*P *< 1.0E-04). Scale bars: 24 µm (**B**), 420 nm (**D**). Source data are available online for this figure.

To prioritize predicted TF candidates for further study, we examined their expression profiles in our scRNA-seq dataset (Fig. 8A). TFs expressed in a very limited number of cells, or whose expression declined along the timeline of Golgi recovery, were excluded from further analysis. The remaining candidates (highlighted in pink in Fig. 8A) were subjected to RNAi-based screening. Each TF was silenced using specific siRNAs. The Golgi complex was inactivated, and reassembly of the new Golgi structures was monitored via labeling of GALNT2, which typically marks substantial structural and functional recovery during biogenesis (see Fig. 2C and Appendix Fig. S3E,F). We found that silencing SP1, MITF, CUX1, and CREB3L1 significantly inhibited GALNT2 signal recovery in the perinuclear region (Fig. 8B,C). Further validation via EM confirmed severe impairment of Golgi reconstitution in cells silenced for SP1, MITF, and CREB3L1, as mainly vacuolar Golgi precursors can be visualized but not Golgi stacks (Fig. 8D), suggesting that these TFs are involved in de novo Golgi biogenesis.

Next, we tested whether the inhibitory effects of SP1, MITF, and CREB3L1 silencing on Golgi reconstitution were accompanied by suppression of *Golgi gene* induction. Using FISH in cells recovering Golgi after inactivation, we observed that depletion of CREB3L1 markedly reduced the induction of all tested *Golgi genes* (*UST*, *GALNT2*, *COG5*, and *GOLGB1*) (Fig. 8E). In contrast, silencing SP1 or MITF had no effect on the activation of these genes (Fig. 8E). These findings suggest that CREB3L1 is a key driver of *Golgi gene* activation during organelle biogenesis, while SP1 and MITF may play more limited or indirect roles.

The Golgi-specific role of CREB3L1 was further confirmed in cells maintained under steady-state conditions (i.e., without Golgi inactivation). First, we silenced all 3 TFs using specific siRNAs (Appendix Fig. S4A). Then, using a panel of *Golgi genes*, we found that CREB3L1 knockdown was able to reduce the expression of some of them, while many were unexpectedly induced by MITF and especially SP1 silencing (Appendix Fig. S4B). In parallel, EM analysis of Golgi ultrastructure revealed significant hypertrophy of pre-Golgi vesicular-tubular membranes at the ERES areas in CREB3L1- silenced cells (Appendix Fig. S4C) indicating possible impact on the transformation of ER-derived membranes into Golgi cisternae. In contrast, no such phenotype was observed in cells exposed to SP1- or MITF-specific siRNAs (Appendix Fig. S4C). Finally, we attempted to mimic Golgi gene induction during biogenesis by overexpressing TFs. We found that overexpression of CREB3L1 induced the expression of most tested *Golgi genes*, whereas SP1 and MITF overexpression induced only a few of them (Appendix Fig. S4D). Together, these data position CREB3L1 as the most compelling TF candidate responsible for *Golgi gene* activation during de novo Golgi biogenesis since CREB3L1 expression is both necessary and sufficient to induce expression of *Golgi genes* and biogenesis of the organelle.

**Figure 9:**
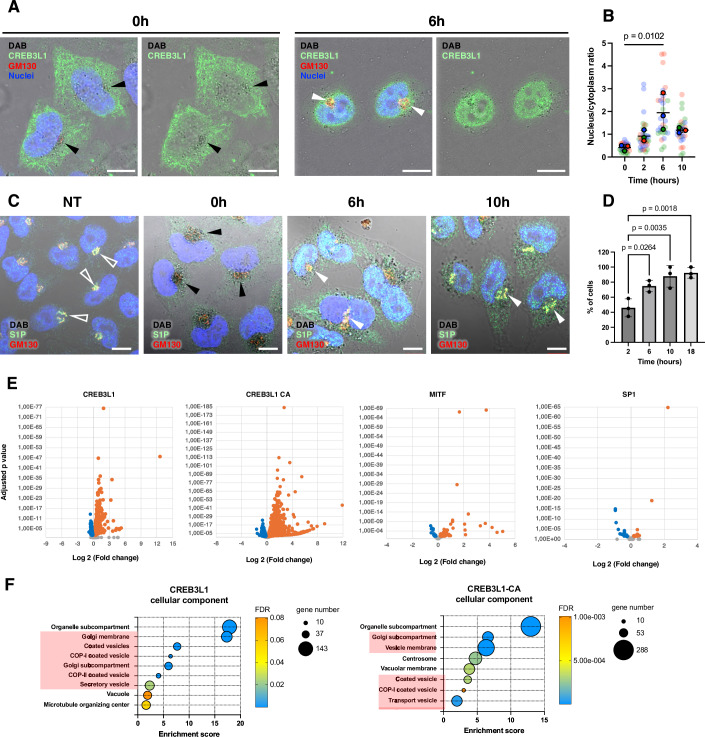
CREB3L1 activation and its impact on Golgi-related genes. (**A**, **B**) ManII-HRP cells were transfected with CREB3L1, subjected to Golgi inactivation and allowed to regenerate new Golgi. Cells were then fixed and immunostained for CREB3L1 and GM130. (**A**) Black arrows indicate DAB-labeled, crosslinked Golgi. A prominent translocation of CREB3L1 to the nucleus was observed at 6 h post-inactivation. White arrows in (**A**) indicate newly forming Golgi structures. Quantification in (**B**) shows an increase in the nuclear-to-cytoplasmic ratio of CREB3L1 signal, peaking at 6 h (mean ± SD, one-way ANOVA; *n* = 3 replicates). (**C**, **D**) ManII-HRP cells were subjected to Golgi inactivation and then allowed to regenerate new Golgi. The cells were fixed and immunostained for site-1 protease (S1P) and GM130. In (**C**), empty arrows indicate S1P localization within the Golgi in untreated cells, black arrows indicate S1P loss within DAB-labeled, crosslinked Golgi, white arrows indicate S1P signal recovery in the newly forming Golgi. Quantification in (**D**) shows the % of cells with S1P-positive Golgi at the different time point after incubation with DAB (mean ± SD, one-way ANOVA; *n *= 3 replicates). (**E**) HeLa cells were transfected with CREB3L1, its constitutively active form (CREB3L1-CA), MITF, or SP1, followed by bulk RNA-seq analysis. Volcano plots illustrate the overall transcriptional impact of each transcription factor, with significantly upregulated and downregulated genes highlighted in orange and blue, respectively. CREB3L1 and CREB3L1-CA induced significantly broader transcriptomic changes compared to the other factors. (**F**) Gene Ontology enrichment analysis of upregulated transcripts in CREB3L1- and CREB3L1-CA-expressing cells revealed significant enrichment in Golgi-related functional annotation terms (highlighted in pink). Scale bars: 10 µm (**A**, **C**). Source data are available online for this figure.

This conclusion was further supported by several lines of evidence. First, bioinformatic analysis of the Golgi gene set identified from the scRNA-seq experiment revealed that the promoter regions of a substantial fraction of these genes contain predicted CREB3L1-binding motifs (Fig. 8F,G). Second, we found that CREB3L1 was functionally activated during Golgi biogenesis. CREB3L1 activation has been proposed to involve trafficking to the Golgi, where proteolytic cleavage releases an N-terminal fragment that translocates to the nucleus and induces transcription of target genes (Murakami et al, 2009; Sampieri et al, 2019). Consistent with this model, during Golgi reconstitution, we observed a progressive increase in nuclear CREB3L1 that peaked at 6 h (Fig. 9A,B), preceding the large-scale induction of *Golgi genes*. This timing coincided with the reappearance of site-1 protease (S1P), which is required for CREB3L1 cleavage and subsequent activation, in the newly forming Golgi (Fig. 9C,D).

Finally, bulk RNA-seq of cells overexpressing either full-length CREB3L1 or its constitutively active N-terminal fragment (CREB3L1-CA) showed significant enrichment of upregulated *Golgi genes* (Fig. 9E,F; Datasets EV3 and EV4), including many genes induced during Golgi biogenesis (Dataset EV1). In contrast, no such enrichment was detected in cells overexpressing MITF or SP1 (Datasets EV5 and EV6), supporting the specificity of CREB3L1 for *Golgi gene* activation.

Importantly, we showed that CREBL1 activation is specifically induced by Golgi damage and biogenesis. We indeed did not observe CREB3L1 nuclear translocation after endosome inactivation (Fig. EV4E,F), in line with the absence of Golgi gene induction after endosome crosslinking (Fig. EV4C,D). By contrast, lysosome inactivation induced nuclear translocation of TFEB (Fig. EV4E,F), a master regulator of endo-lysosomal genes (Sardiello et al, 2009), whose activation has been documented after lysosome crosslinking (Fig. EV4D). Taken together, these results indicate that the biogenesis of distinct organelles is supported by organelle-specific transcriptional programs governed by master transcription factors, such as CREB3L1 for Golgi.

## Discussion

Organelle biogenesis is a fundamental cellular process that enables cells to adapt to environmental cues, developmental signals, and metabolic demands. In this context, Golgi biogenesis plays a central role in supporting increased biosynthetic traffic and expanding Golgi structures during processes such as cell differentiation (Lu et al, 2001; Kirk et al, 2009; Valenzuela and Perez, 2015; García et al, 2017). While transcriptional regulation of biogenesis has been well characterized for such organelles as lysosomes and mitochondria (Sardiello et al, 2009; Puertollano et al, 2018; Houten and Auwerx, 2004), the transcriptional control of Golgi biogenesis remains poorly defined.

Most prior studies have examined Golgi-related transcriptional changes in the context of stress responses (Taniguchi et al, 2016; Taniguchi and Yoshida, 2017; Reiling et al, 2013; Baumann et al, 2018). However, these models are often confounded by cytotoxicity of chemical stressors, making it difficult to isolate Golgi-specific transcriptional events. An ideal system would enable controlled, synchronous induction of de novo Golgi formation, thereby allowing direct interrogation of the associated transcriptional remodeling. Until now, such a model has been lacking. Here, we demonstrate that the assay we developed enables the investigation of Golgi biogenesis mechanisms, including those at the transcriptional level.

### Transcriptional activation of Golgi genes is required for de novo Golgi biogenesis

Using this system, we addressed the previously unanswered question of whether the induction of a comprehensive set of *Golgi genes* contributes to the morphogenesis of newly forming Golgi organelles. Our finding that massive activation of *Golgi genes* coincides with the structural and functional maturation of nascent Golgi units suggests this may indeed be the case. In line with this notion, we provide direct evidence that transcription is required for Golgi reformation. Inhibition of transcription prevented the formation of mature Golgi stacks, leading instead to irregular, immature Golgi-like structures (see Fig. 4). Similar Golgi precursors can be observed at the early stages of Golgi development, i.e. before massive activation of the *Golgi genes* (Fig. 1B). These precursors contain some Golgi proteins (like GM130, GRASP65, p115, and β-COP) whose significant pools reside in cytosol and/or ERGIC (Marra et al, 2001; Nelson et al, 1998; Scales et al, 1997) from where they can be rapidly recruited to support initial stage of Golgi morphogenesis. However, in the absence of active transcription, these nascent structures failed to mature into functionally competent and normally shaped Golgi complexes.

Silencing of CREB3L, which we identified as a key transactivator of *Golgi genes*, was sufficient to arrest Golgi development at the stage of immature precursors (Fig. 8D). These observations indicate that transcriptional activation of *Golgi genes* is not merely supportive but essential for the later stages of biogenesis—particularly those restoring Golgi stacking, trafficking, and enzymatic function. Indeed, we observed a strong correlation between transcriptional activation of *Golgi genes* at ~10 h post-inactivation and the emergence of functional, stacked Golgi structures. This timeline closely mirrors Golgi recovery kinetics following laser ablation (Tängemo et al, 2011; Ronchi et al, 2014), reinforcing the notion that loss of Golgi triggers a transcriptional program essential for its reconstruction.

Another key question we addressed was whether cells require a broad activation of *Golgi genes* to rebuild the organelle, or only the activation of a compartment- or function-specific subset, to rebuild the organelle. We also asked whether activation of such subsets occurs in a sequential manner. Previous studies suggest that Golgi assembly after chemical disruption proceeds in a *cis*-to-*trans* order (Alcalde et al, 1992; Puri and Linstedt, 2003). Therefore, we did not rule out the possibility that the cell might first activate *cis*-Golgi genes, followed by genes associated with the *trans*-Golgi compartment.

However, we observed that the activated genes spanned diverse Golgi compartments and functional classes, suggesting that Golgi regeneration is orchestrated as a coordinated transcriptional program rather than a stepwise activation of specific modules. Genes encoding *cis*- and *trans-*Golgi proteins, as well as those involved in glycosylation, trafficking, and ion transport, were all upregulated with similar kinetics.

This broad activation supports the idea of a unified Golgi regulon, enabling the simultaneous expression of components required for the organelle structural and functional integrity. Such coordination is likely critical for processes like glycosylation, which requires the synchronized activity of enzymes localized to distinct sub-Golgi regions, along with transporters supplying substrates and ion cofactors (Freeze and Ng, 2011; Foulquier and Legrand, 2020). In addition, these components may be critical for cisternal morphogenesis, as the absence of their expression prevents the transformation of vacuolar membranes in Golgi precursors into the flattened disks characteristic of mature cisternae. Thus, our findings suggest that Golgi biogenesis is driven by a highly integrated transcriptional mechanism, fine-tuned to ensure that the growing organelle acquires normal architecture and full functional capacity.

### Is CREB3L1 the master regulator of the Golgi gene network?

Organelle-specific gene networks are typically governed by master transcriptional regulators—for instance, TFEB and TFE3 for lysosomes (Sardiello et al, 2009; Puertollano et al, 2018), or PGC1α for mitochondria (Houten and Auwerx, 2004). The coordinated activation of Golgi-associated genes during de novo Golgi biogenesis suggests the existence of a similar regulatory mechanism for the Golgi apparatus.

We identified three transcription factors—SP1, MITF, and CREB3L1—whose silencing significantly impaired Golgi reassembly. CREB3L1 was particularly relevant as knockdown robustly suppressed the induction of all tested *Golgi genes* in cells undergoing Golgi reconstruction (see Fig. 8E). In contrast, silencing SP1 and MITF did not produce the same inhibitory effect. This points to a more selective and central role for CREB3L1 in Golgi gene activation during organelle biogenesis.

Golgi specificity was further supported by CREB3L1 gain- and loss-of-function experiments. Overexpression of CREB3L1 (or its constitutively active variant) induced *Golgi gene* expression, while its knockdown led to repression of some *Golgi genes*. Notably, SP1 or MITF did not produce comparable effects, as their silencing in some cases paradoxically increased expression of *Golgi genes*. These findings suggest that while SP1 and MITF may contribute to Golgi biogenesis, their roles are likely indirect or involve additional, yet undefined, mechanisms.

CREB3L1 is a member of the CREB3 transcription factor family, several of which have been implicated in transcriptional regulation of secretory pathway genes, including those involved in ER-to-Golgi trafficking and Golgi function (Sampieri et al, 2019; García et al, 2017; Barbosa et al, 2013). Previous studies have shown that CREB3L1 and its homolog CREB3L2 are activated in response to increased secretory load and are required for the transcriptional upregulation of genes supporting Golgi expansion (García et al, 2017; Pittari et al, 2022; Al-Maskari et al, 2018), further supporting our findings.

Promoter analysis revealed putative CREB3L1-binding sites in a substantial number of *Golgi genes* upregulated during biogenesis (Fig. 8F,G; Dataset EV1). Interestingly, some CREB3L1-responsive genes (e.g., *COG5*, *UST*) lacked predicted CREB3L1 motifs, yet their activation was still inhibited by CREB3L1 silencing. This suggests the possibility of indirect regulation or the involvement of non-canonical binding sites not yet annotated in current databases.

Taken together, our data position CREB3L1 as a key transcriptional driver and candidate master regulator of Golgi biogenesis, able to coordinately activate a broad set of Golgi-associated genes during organelle formation.

### How does CREB3L1 sense the cellular need to activate Golgi genes?

We found that biogenesis of a new Golgi apparatus is accompanied by increased nuclear translocation of CREB3L1 (Fig. 9A,B), indicating its activation (Kondo et al, 2005; Sampieri et al, 2019). CREB3L1, also known as OASIS, is a basic leucine zipper transcription factor of the CREB/ATF family that contains a transmembrane domain anchoring it to the ER (Kondo et al, 2005; Sampieri et al, 2019). Upon ER overload, CREB3L1 undergoes proteolytic cleavage, releasing an N-terminal fragment that translocates to the nucleus and activates target genes (Kondo et al, 2005; Sampieri et al, 2019). Because the proteases mediating this cleavage (e.g., S1P) reside in the Golgi, CREB3L1 activation likely requires trafficking from the ER to the Golgi, analogous to other ER-resident transcription factors activated by regulated intramembrane proteolysis, such as ATF6 and SREBPs (Murakami et al, 2006; Sampieri et al, 2019).

How might the newly forming Golgi communicate with ER-localized CREB3L1 to promote its activation remains to be understood. It may be due to cargo accumulation in reforming immature Golgi compartment. It has indeed been shown that the Golgi is able to sense cargo load and activate signaling circuits to handle it (Luini and Parashuraman, 2016). Our data indicate that at early stages the newly forming Golgi can receive cargo but cannot efficiently export it. It is therefore plausible that this overload activates signaling cascades that promote CREB3L1 trafficking, potentially via phosphorylation and/or conformational changes.

Another, non-mutually exclusive possibility is that CREB3L1 indirectly senses a trafficking bottleneck in the newly forming Golgi. Our data suggest that during the initial stages of Golgi regeneration (2–6 h), retrograde trafficking from newly formed Golgi units to the ER is impaired, leading to accumulation of the ER chaperone ERp44 in the Golgi (Fig. EV2E,F). The availability of ER chaperones has been shown to regulate ER retention of certain transcription factors (e.g., ATF6), whereas chaperone depletion promotes their export to the Golgi, where cleavage and activation occur (Shen et al, 2002). A similar mechanism may apply to CREB3L1: retention of chaperones in the newly forming Golgi may reduce their availability in the ER, thereby facilitating CREB3L1 export to the Golgi, cleavage by S1P, and release of the N-terminal fragment to activate target genes in the nucleus. Consistent with this model, ERp44 retention in the newly forming Golgi coincides temporally with both the peak of CREB3L1 nuclear translocation and the repopulation of the regenerating Golgi by S1P, which would enable CREB3L1 cleavage and activation.

The resulting CREB3L1-dependent transcriptional activation of *Golgi genes* would be expected to promote functional maturation of the new Golgi and thereby expand secretory capacity. Consistent with this model, CREB3L1 activation precedes structural and functional maturation of the organelle: nuclear translocation of CREB3L1 increases and peaks at ~6 h, whereas the large-scale induction of target *Golgi genes* is detected at ~10 h, coincident with the time at which the Golgi begins to acquire mature architecture and trafficking capacity.

### Transcriptional program of Golgi biogenesis mirrors physiological secretory remodeling

It is important to note that, in addition to CREB3L1/2, several TFs have been proposed to regulate Golgi-associated genes. These factors typically emerge from studies analyzing transcriptional responses under so-called Golgi stress, induced by chemical agents that disrupt Golgi architecture and function. However, the identity of the activated Golgi gene sets and their regulatory TFs vary significantly across studies, suggesting that Golgi stress responses are highly context-dependent and shaped by the nature of the stressor.

In this study, we sought to determine whether mechanisms implicated in the Golgi stress response also contribute to Golgi biogenesis. When comparing gene sets activated by three distinct Golgi stressors—Brefeldin A (BFA), Monensin, and Golgicide (Baumann et al, 2018)—to the set of genes activated during Golgi biogenesis in our model, we observed minimal overlap (Appendix Fig. S5A). A similarly low degree of overlap (Appendix Fig. S5B) was observed with genes activated in response to the Golgi stressor OSW-1 (Sasaki et al, 2025). These findings indicate that the transcriptional programs underlying Golgi biogenesis and Golgi stress responses are largely distinct. Similar to the ER, the Golgi harbors multiple stress-responsive pathways (Taniguchi and Yoshida, 2017). Indeed, Golgi stress has been shown to activate TFs such as MLX, TFE3, ELK1, and CREB3 (Taniguchi et al, 2016; Taniguchi and Yoshida, 2017; Reiling et al, 2013; Baumann et al, 2018). Among these, only CREB3 belongs to the same family as CREB3L1, which we identified as a specific regulator of the Golgi gene network during biogenesis. However, whether CREB3 activates the same network is unknown, as information about its Golgi gene targets under BFA-induced stress conditions remains limited to *ARF4* (Reiling et al, 2013).

Interestingly, the transcriptional program activated during de novo Golgi biogenesis appears to resemble the gene expression changes observed during cell differentiation, where there is a well-documented need for expanded Golgi capacity to support increased secretory activity. For instance, during differentiation of naïve B cells into antibody-secreting plasma cells, the Golgi drastically expands nearly sevenfold (Kirk et al, 2009), accompanied by a robust induction of Golgi-associated genes (Kassambara et al, 2021). Remarkably, nearly half of the *Golgi genes* we identified during Golgi biogenesis overlapped with those induced during B cell differentiation (Appendix Fig. S5C). A significant overlap was also observed with *Golgi genes* upregulated during differentiation of endometrial stromal cells (Appendix Fig. S5D), which remodel the Golgi to support secretion of implantation-related factors (Pittari et al, 2022). Notably, gene activation in both differentiating B cells and endometrial cells requires the activity of CREB3L1 and/or CREB3L2 (Al-Maskari et al, 2018; Pittari et al, 2022).

These findings suggest that the transcriptional program governing Golgi biogenesis during organelle reassembly is shared with physiological states requiring secretory adaptation. This is further supported by reports implicating CREB3L1 in Golgi expansion during differentiation of thyroid epithelial cells and neurons (García et al, 2017; Chung et al, 2017). Collectively, our results argue that Golgi biogenesis is regulated by a specific transcriptional program that operates not only during organelle regeneration but also in biologically relevant contexts of cellular differentiation and response to stimuli activating secretion.

## Methods

**Table:** Reagents and tools table

Reagent/resource	Reference or source	Identifier or catalog number
**Experimental models**		
WT HeLa	ATCC	CCL-2
HeLa ManII-HRP	Jollivet et al, 2007	
HeLa ManII-HRP GalT-EGFP	This study	
Lenti-X™ 293T Cell Line	Takara Bio	632180
**Recombinant DNA**		
Str-KDEL_SBP-EGFP-GPI	Boncompain et al, 2012	
Str-KDEL_IRES_SBP mCherry-CD-M6PR	Chen et al, 2017	
Str-KDEL_IRES_SBPmCherry-Lamp1	Chen et al, 2017	
pLVX-EF1a-GalT-IRES-Puromycin	Addgene	#134862
pEGFP-N1-MITF-M	Addgene	#38131
CMV_SP1_HA	VectorBuilder Inc	VB250121-1102aju
pcDNA3.1FLCREB3L1	Kindly provided by Dr Álvarez C. Universidad Nacional de Córdoba	
pcDNA3.1CREB3L1CA	Kindly provided by Dr Álvarez C. Universidad Nacional de Córdoba	
**Antibodies**		
BetaCop	Abcam	ab2899
COG5	Abnova	PAB21288
TGN46	Bio-Rad	AHP500
GM130	BD Biosciences	610823
GALNT2	Sinobiological	13764-T62
ARF1	Proteintech	10790-1-AP
p115	Proteintech	13509-1-AP
RAB6A	Proteintech	10187-2-AP
GRASP65	Thermo Fisher Scientific	PA3- 910
Giantin	Kindly provided by Dr. M.A. De Matteis, TIGEM, Pozzuoli, Italy	
GFP	Abcam	ab290
S1P	Novusbio	NBP3-05161
TFEB	Cell Signaling	#4240
ERP44	Kindly provided by Dr T. Anelli, I.R.C.C.S. Ospedale San Raffaele, Milano	
**Oligonucleotides and other sequence-based reagents**		
siRNA sequences	This study	Appendix Table S1
qPCR primers	This study	Appendix Table S2
RNAscope™ Probe COG5	Advanced Cell Diagnostics, part of Bio-Techne	#1297181
RNAscope™ Probe GALNT2	Advanced Cell Diagnostics, part of Bio-Techne	#1053611
RNAscope™ Probe GOLGB1	Advanced Cell Diagnostics, part of Bio-Techne	#1297191
RNAscope™ Probe UST	Advanced Cell Diagnostics, part of Bio-Techne	#851011
**Chemicals, enzymes and other reagents**		
DMEM	Euroclone	ECB7501L
FBS	Euroclone	ECS0186L
L-Glutamine	Euroclone	ECB3000D
Penicillin/Streptomicin	Euroclone	ECB3001
Polybrene	Sigma-Aldrich	TR-1003-50UL
Cytochalasin D	Sigma-Aldrich	C8273
DMEM/F-12, GlutaMAX™	Thermo Fisher Scientific	10565018
Lipofectamine™ RNAiMAX	Thermo Fisher Scientific	13778075
Opti-MEM™	Thermo Fisher Scientific	31985070
3,3’-Diaminobenzidine	Sigma-Aldrich	D8001
Mowiol	Sigma-Aldrich	D2522
1.4 nm gold-conjugated Fab’ fragment of anti-rabbit IgGs	Nanoprobes	2004-1 ML
GoldEnhance™ EM kit	Nanoprobes	2113-8 ML
LipoD293™	SignaGen Laboratories	50-478-2
Lipofectamine 3000	Thermo Fisher Scientific	L3000001
Biotin	Sigma-Aldrich	B4501
Pierce™ Horseradish Peroxidase	Thermo Fisher Scientific	31491
RNA Synthesis assay kit	Abcam	ab228561
GEL/MOUNT	Biomeda	G 0918
RNEasy Mini Kit	Qiagen	74104
QuantiTect Reverse Transcription	Qiagen	205311
Light Cycler 480 Sybr Green I Master Mix	Roche	04707516001
**Software**		
Prism 10.4.1	GraphPad	
Fiji 1.54	Open source	
Illumina NovaSeq X base call BCL Convert	Illumina	
BBDuk	JGI	
STAR Aligner 2.6.0a	Open Source	
NIS-Elements Software	Nikon	
ZEN 3.0	Zeiss	
iTEM	Soft Imaging Systems GmbH	
Tomography 5	Thermo Fisher Scientific	
iMOD	Open source	
**Other**		
Qubit 4.0 fluorimetric Assay	Thermo Fisher Scientific	For Library Preparation
NovaSeq X sequencing system using a single-end, 100-cycle strategy	(Illumina Inc.)	
FiberLite F21-8×50 rotor	Sorvall	
LSM 700	Zeiss	
LSM 800	Zeiss	
Leica EM UC7	Leica Microsystems	
Tecnai G2 Spirit BioTwin electron microscope	Thermo Fisher Scientific	
VELETTA CCD digital camera	Soft Imaging Systems GmbH	
Talos L120C electron microscope	Thermo Fisher Scientific	
Nikon TE2000-E2	Nikon	
LightCycler 480 II Real-Time System	Roche	

### Antibodies, plasmids, and other reagents

Primary antibodies against following proteins were used in the study: BetaCop (Abcam, ab2899), COG5 (Abnova, PAB21288), TGN46 (Bio-Rad, AHP500), GM130 (BD Biosciences, 610823), GALNT2 (Sinobiological, 13764-T62), Syntaxin5 (Synaptic System, 110 053), ARF1 (Proteintech, 10790-1-AP), p115 (Proteintech, 13509-1-AP), RAB6A (Proteintech, 10187-2-AP), GRASP65 (Thermo Fisher Scientific, PA3-910), Giantin (kindly provided by Dr. M.A. De Matteis, TIGEM, Pozzuoli, Italy), GFP (Abcam, ab290), S1P (NovusBio, NBP3-05161), ERP44 (Kindly provided by Dr T. Anelli, I.R.C.C.S. Ospedale San Raffaele, Milano), TFEB (Cell Signaling, #4240). Secondary anti-rabbit, anti-mouse, and anti-sheep antibodies conjugated with Alexa Fluor 488 or 568 were purchased from Life Technologies (Carlsbad, CA). GPI-GFP-RUSH (Str-KDEL_SBP-EGFP-GPI) DNA constructs for trafficking experiments was reported previously (Boncompain et al, 2012). LAMP1-mCherry-RUSH (Str-KDEL_IRES_SBP-mCherry-Lamp1) and M6PR-mCherry-RUSH (Str-KDEL_IRES_SBP-mCherry-CD-M6PR) constructs (Chen et al, 2017) were obtained from Addgene. CREB3L1 construct was kindly provided by Dr. Cecilia Alvarez (Universidad Nacional de Córdoba, Córdoba, Argentina). RNA Synthesis assay kit was obtained from Abcam (ab228561). pEGFP-N1-MITF-M (Addgene # 38131) and CMV_SP1_HA (VectorBuilder Inc, Vector ID VB250121-1102aju) were used to overexpress MITF and SP1 in HeLa cells.

### Cell culture and cytoplast generation

HeLa ManII-HRP cells were cultured in Dulbecco’s Modified Eagle Medium (DMEM) supplemented with 10% fetal bovine serum (FBS), penicillin/streptomycin, and 2 mM L-glutamine. The cells were kept in mycoplasma-free conditions and tested for mycoplasma contamination monthly.

HeLa ManII-HRP cell line stably expressing GalT-GFP were generated using transduction with lentiviral particles. pLVX-EF1a-GalT-IRES-Puromycin cDNA plasmid (Addgene #134862), was used to generate lentiviral particles using a second-generation packaging system in X293T (Takara Bio) cells. HeLa ManII-HRP cells were transduced with lentiviral particles at a multiplicity of infection (MOI) 30, in the presence of 8 μg/mL polybrene (Sigma-Aldrich) to enhance transduction efficiency. Cells were incubated with the virus-containing medium for 24 h, after which the medium was replaced with fresh culture medium. In all, 48–72 h post-infection, transduced cells were selected with puromycin.

Generation of nuclear-free cells (cytoplast) was performed by centrifugation of cells grown on glass coverslips (Prescott et al, 1972). Thirty minutes prior to centrifugation, cytochalasin D (Sigma-Aldrich, C8273) was added to the culture media at 5-mg/ml final concentration. Coverslips were centrifuged at 10,000 RPM in a FiberLite F21-8×50 rotor (Sorvall) for 30 min at 35 °C in media containing cytochalasin D. Immediately after centrifugation, the coverslips were rinsed four times with culture media and incubated in fresh DMEM/F12 GlutaMax medium supplemented with 10% FBS for 1 h at 37 °C in 5% CO_2_ prior to DAB-mediated inactivation of the Golgi.

### RNA interference

Gene silencing was performed using siRNAs delivered with Lipofectamine™ RNAiMAX Transfection Reagent (Thermo Fisher Scientific, Cat. No. 13778075), according to the manufacturer’s instructions. Cells were transfected with siRNA complexes prepared in Opti-MEM™ Reduced Serum Medium. For each target gene, transfections were carried out using a combination of two or three distinct siRNA sequences to maximize silencing efficiency and minimize off-target effects. Final siRNA concentrations and transfection conditions were optimized based on preliminary titration experiments. Cells were processed for downstream analyses 48–72 h post-transfection, depending on the assay. Silencing efficiency was assessed by qRT-PCR. All siRNA sequences used in this study are listed in Appendix Table S1.

### DAB-mediated Golgi inactivation

DAB-mediated Golgi inactivation was performed with modifications to previously published protocols. A 10× DAB stock solution was prepared by dissolving 50 mg of 3,3′-diaminobenzidine (DAB; D8001, Sigma-Aldrich) powder in 0.1 M HCl. The 1× working solution was obtained by diluting the stock solution in 0.1 M Tris-HCl (pH 7.4) immediately before use.

Cells expressing ManII-HRP were incubated with 1× DAB and 0.02% H₂O₂ at room temperature directly on the light microscope stage. Unlike previous protocols, cells were not incubated on ice to minimize cellular stress during the procedure. DAB polymerization was monitored in real time by bright-field microscopy, and the reaction was stopped as soon as a detectable light-brown precipitate appeared in the Golgi, indicating localized DAB deposition. This approach helps prevent excessive DAB accumulation in the Golgi, which can cause membrane rupture and increase cell damage. The reaction was terminated by washing the cells with phosphate-buffered saline (PBS). Following the reaction, cells were maintained in conditioned medium to stimulate formation of the new Golgi organelle and to analyze this process with different approaches (see below).

### Immunofluorescence (IF)

To assess the morphology and composition of the newly forming Golgi the cells were fixed at different time points after DAB-mediated inactivation with 4% paraformaldehyde (PFA) in PBS for 10 min, followed by a 30-min incubation in a blocking/permeabilization solution containing 1%BSA, 0.5% saponin and 50 mM NH₄Cl in PBS. Primary antibodies were diluted in the blocking/permeabilization solution and incubated with cells for 1 h or overnight. Secondary antibodies were similarly diluted and incubated for 45–60 min. The labelled cells were mounted in Mowiol (Sigma, D2522) and examined using a ZEISS LSM 700 or LSM 800 confocal microscope using a 63× 1.4 NA oil immersion objective and appropriate settings of excitation and emission for image capture. For each Golgi marker the percentage of cells with marker-positive new Golgi was calculated at each time point after DAB-mediated inactivation.

### Electron microscopy (EM)

Ultrastructure of the newly forming Golgi was analyzed in ManII-HRP HeLa cells at different time points after DAB-mediated inactivation. For immuno-electron microscopy analysis, the cells were prepared as described previously (Polishchuk et al, 2003). Briefly, after fixation with mixture of 4%PFA and 0.05% glutaraldehyde in 0.2 M HEPES for 15 min and with 4%PFA alone for 30 min, the cells were incubated with blocking/permeabilizing solution: 0.5% bovine serum albumin (BSA), 0.1% saponin, 50 mM NH_4_Cl in PBS for 20–30 min. Primary antibody against either GM130 (BD Biosciences, Franklin Lakes, NJ, USA) or GFP (Abcam, Cambridge, UK) were diluted in blocking/permeabilizing solution and added to the cells overnight, followed by 2 h incubation with 1.4 nm gold-conjugated Fab’ fragment of anti-rabbit IgGs (Nanoprobes, Yaphank, NY, USA). GoldEnhance™ EM kit (Nanoprobes, Yaphank, NY, USA) was used to enhance ultrasmall gold particles. Then cells were scraped, pelleted, post-fixed in OsO4 and uranyl acetate and embedded in Epon.

For routine EM, the samples were fixed in 1% glutaraldehyde in 0.2 M HEPES for 60 min and post-fixed and embedded as indicated above. From each Epon-embedded sample, thin 60-nm sections were cut using a Leica EM UC7 (Leica Microsystems, Wetzlar, Germany). EM images were acquired from thin sections under Tecnai G2 Spirit BioTwin electron microscope (Thermofisher, Eindhoven, The Netherlands) equipped with a VELETTA CCD digital camera (Soft Imaging Systems GmbH, Munster, Germany). Inactivated Golgi compartments and their remnants were identified based on the presence of electron-dense DAB polymer. Newly forming Golgi units were identified in immune-EM specimens based on (i) the presence of GM130-associated gold particles and (ii) absence of DAB. The structures with similar ultrastructural features in routine EM specimens were considered as newly forming Golgi membranes and were arbitrary divided into three main classes vacuolar clusters, irregular stacks and regular stacks for morphometric analysis.

### Electron tomography

Epon sections (250 nm thick) from cell recovering Golgi were collected on Formvar carbon-coated slot grids and analyzed using a Talos L120C electron microscope (Thermo Fisher Scientific) equipped with an automated tomography stage. The single tilt series of images were acquired in a range of −65° to +65° (at 1° intervals) using Tomography 5 Software (Thermo Fisher Scientific). Tilt series were used with the open-source IMOD software to generate tomograms.

### RUSH assay of membrane trafficking

Ability of the newly forming Golgi to support trafficking of different cargo proteins was evaluated with RUSH (Retention Using Selective Hooks) approach. LipoD293™ (SignaGen Laboratories, Frederick, MD, USA) or Lipofectamine 3000 (Thermo Fisher Scientific) were used to transfect RUSH cDNA constructs encoding reporters directed to either plasma membrane (GPI-GFP-RUSH) or endo-lysosomal compartments (M6PR-mCherry-RUSH, LAMP1-mCherry-RUSH). RUSH cargoes release from endoplasmic reticulum in control or DAB-inactivated ManII-HRP HeLa cells was induced by adding pre-warmed Leibovitz medium (Life Technologies) supplemented with 40 μM biotin (Sigma-Aldrich, Cat. No. B4501). The efficiency of RUSH cargo trafficking to different compartments of the biosynthetic route was assessed by IF or immuno-EM in cells fixed at the different intervals after release of the reporters from the ER. The analysis of M6PR-mCherry-RUSH or LAMP1-mCherry-RUSH delivery to target destination was conducted based on quantification of number of RUSH cargo-positive post-Golgi structures per cell.

### CATCHFIRE-based retrograde Golgi-to-ER trafficking assay

The CATCHFIRE system utilizes a fluorogenic proximity-control method founded on chemically induced dimerization, wherein two engineered components—FIREMate and FIREtag—selectively interact upon addition of a small-molecule ligand termed “match” (Bottone et al, 2023). In this investigation, HeLa cells were transfected with a construct encoding FIREMate-KDEL and ManII-mApple-FIREtag. To monitor retrograde trafficking, FIREMate-KDEL served as an ER-localized anchor to capture the ManII-mApple-FIREtag reporter as it recycles from the Golgi apparatus. Cells were maintained in match (10 µM) immediately following transfection to promote ER retention of ManII-mApple-FIREtag. Following Golgi inactivation, the match ligand was washed out for 1 h, allowing ManII-mApple-FIREtag to localize to the newly formed Golgi complex. Subsequently, the ligand was reintroduced for periods of 1 and 2 h to facilitate recapture of ManII-mApple-FIREtag within the ER, thereby enabling assessment of retrieval efficiency from the Golgi via confocal microscopy.

### Live-cell microscopy

Cells were grown on #1.5 glass coverslips in Petri dishes for 48–72 h. After DAB-mediated inactivation of Golgi, phenol-red free mixture of DMEM/F-12 containing 10% FBS was added to the cells, and coverslips were mounted on Rose chambers and placed on the microscope stage. The chambers were maintained at 37.0 ± 0.3 °C within a custom-built enclosure. Golgi regrowth and trafficking were acquired in multi-mode (differential interference contrast [DIC], bright-field, and wide-field fluorescence) time-lapse recordings on a Nikon TE2000-E2 microscope equipped with a PlanApo 60X NA 1.4 oil-immersion DIC H objective (Nikon) and LED illuminator (CoolLED *p*E-4000). Recordings were done at 10-15 min intervals at 1 mm z-steps and captured on an Andor Zyla (VSC-04182) camera at 0.22 mm XY pixel size. The system was controlled by NIS-Elements Software.

All time-lapse recordings acquired with Nikon TE2000-E2 microscope and used for quantification, were analyzed using Fiji. For the quantification of GalT-GFP, all time-lapse recordings were acquired using identical illumination settings. The mean pixel values of GalT-GFP were calculated from maximum-intensity projections (MIP) of z-stacks with a region-of-interest (ROI) drawn around the Golgi at each timepoint. The Golgi areas were identified based on DAB deposition observed in the bright-field channel at each timepoint. The mean pixel values were then corrected by subtracting out the background signal.

### Endocytic HRP uptake and endosome inactivation

HeLa cells were incubated for 90 min at 37 °C in DHB (DMEM + 20 mM HEPES + 0.1% BSA). Then 2 mg/mL HRP in DHB was added for 30 min at 37 °C, whereupon cells were washed 4 times with cold PBS to eliminate the Extracellular HRP. After HRP uptake, the cells were fixed to assess HRP colocalization with endocytic markers (EEA1 or LAMP1) by confocal microscopy or subjected to inactivation of HRP-loaded endosomes. For inactivation, the cells were incubated in DAB solution (0.1 M Tris HCL pH 7.4 + 0.25 mg/mL DAB + 0.02% H_2_O_2_) for 2 min at room temperature, then were washed in PBS and fixed or re-incubated with fresh medium. To check recovery of functional endosomes, at 18 h post-inactivation HRP was re-added to the cells for 30 min. The cells were then fixed, and the efficiency of HRP uptake was assessed by its IF labeling.

### RNA synthesis assay

Efficiency of RNA-synthesis was evaluated with RNA Synthesis assay kit (Abcam). ManII-HRP HeLa cells were incubated with RNA Label component of the kit for 1 h at 37 °C. Then the cells were fixed and permeabilized according to the manufacturer's protocol. RNA Reaction cocktail was added to the cells for 30 min at room temperature in dark. The cells were then washed three times with Wash Buffer and stained with DAPI for nuclei and antibody of interest (when needed). Red fluorescent signal associated with de novo synthesized RNA was detected using excitation and emission settings for AlexaFluor568 at ZEISS LSM800 confocal microscope. Transcription inhibitor Actinomycin D was used as a control to evaluate the specificity of the labelling.

### Fluorescent in situ hybridization (FISH)

To analyze mRNA levels of Golgi-associated genes, we customized the RNAscope™ FISH protocol (Advanced Cell Diagnostics, USA) based on transcript size, following the manufacturer’s guidelines. RNA in situ hybridization was performed using the RNAscope Multiplex Fluorescent v2 reagent kit (ACD, no. 323285). After Golgi inactivation formalin-fixed ManII-HRP cells were pre-treated with RNAscope Protease III (ACD, no. 322380), diluted in PBS at room temperature, according to a customized protocol designed to preserve the endogenous GFP signal. After pre-treatment, each well was incubated with different probes for corresponding gene (*COG5* #1297181-C3, *GALNT2* #1053611-C3, *GOLGB1* #1297191-C2, *UST* #851011-C3), followed by the amplification cascade as outlined in the manufacturer’s protocol. Detection was performed using TSA Vivid Fluorophore 570 (ACD, no. 323272). Finally, cells were counterstained with DAPI, mounted with GEL/MOUNT (Biomeda, Foster City, CA, USA), and analyzed using a ZEISS LSM900 confocal microscope. Intensity of the FISH labelling per cell was quantified using Fiji software.

### RT-qPCR

To evaluate gene expression levels in silencing and overexpression experiments total RNA was isolated using the RNEasy Mini Kit (Qiagen, Germantown, MD, USA), from treated and control cells. After that, reverse transcription of RNAs was performed using primers indicated in Appendix Table S2 and QuantiTect Reverse Transcription kits (Qiagen). RT-qPCR experiments were performed using Light Cycler 480 Syber Green I Master Mix (Roche, Basel, Switzerland) using a LightCycler 480 II Real-Time System (Roche) in 96-well plates. RT-PCR results were analyzed using the 2^−ΔΔCt^ method, normalized against the housekeeping *ACTB* gene encoding β-actin.

### Single cell RNA sequencing (scRNA-seq)

#### Sample collection

Cells were subjected to DAB-mediated Golgi inactivation as described above. After removal of the DAB solution, the samples were washed in PBS and collected for RNA-seq immediately, or after different incubation intervals (2, 6, 10, 14, 18 or 24 h). For each time point ~100,000 cells were collected and their viability was checked by trypan blue staining to ensure that the percentage of dead cells does not exceed 5%. In parallel from each time point the cells were prepared for EM and IF analysis to assess the degree of morphogenesis of the newly forming Golgi.

#### Sequencing

Libraries were prepared by using the NEGEDIA NGD0050R_single-cell RNA-seq (10X Genomics) Sequencing service (Negedia s.r.l). In brief, single-cells were suspended in phosphate-buffered saline containing 0.04% bovine serum albumin, filtered using 40 µm cell strainer (Biologix), and their concentration was evaluated at LUNA-II™ Automated Cell Counter (Logos Biosystems). The cell suspension was loaded onto the Chromium Single Cell G Chip Kit (10x Genomics) and run on the Chromium Single Cell Controller (10x Genomics) to generate single-cell gel beads emulsion, according to the manufacturer’s protocol. The single-cell 3′ Library and Gel Bead Kit V3.1 (10x Genomics) was used to generate cDNA and the final libraries. The cDNA quality was assessed using High sensitivity D5000 screen tape on the Agilent 4200 TapeStation system (Agilent Technologies). The quality of libraries was assessed by using screen tape High sensitivity DNA D1000 (Agilent Technologies). Finally, the libraries were sequenced on a Novaseq6000 sequencer (Illumina) according to the manufacturer’s specifications.

#### Data pre-processing

Demultiplexing was performed with Cell Ranger (Zheng et al, 2017) (v. 7.1.0), with default settings. Then, a customized reference genome was generated to identify ManII-HRP positive cells by merging the human GRCh38 genome from Ensembl and the ManII-HRP sequence. A customized .gtf file was also generated. Mapping and counting were performed using the Cell Ranger (Zheng et al, 2017) counting function (v. 7.1.0) with predefined parameters and using the customized genome as a reference. Then, 3 files (matrix.mtx.gz, features.tsv.gz and barcodes.tsv.gz) were generated for each sample, which formed the input for the Seurat pipeline (Zheng et al, 2017).

#### Data analysis

Data were analyzed in Rstudio (R version 4.3.0) using the Seurat package [2] (version 4.4). A Seurat object, including only ManII-HRP positive cells was generated (cells containing at least 1 count for ManII-HRP). Cells that expressed more than 500 genes, had more than 1000 counts and less than 15% of mitochondrial gene expression were filtered in. A post-filtered dataset of 13,092 genes expressed in 35,866 cells was used for downstream processing. Count data was then processed using SCTransform (Lause et al, 2021), which normalizes and stabilizes variance through a regularized negative binomial regression and regressed out against the percentage of mitochondrial genes. Using FindVariableFeatures, the 2000 most variable features were identified and then the Principal Component Analysis (PCA) was performed on these features using the runPCA function. Cell clusters were determined via Shared Nearest Neighbor (SNN) and k-Nearest Neighbor (KNN) analyses using the FindNeighbors function with the first 6 Principal Components and FindClusters function with resolution 0.5, respectively. Uniform Manifold Approximation and Projection (UMAP) was performed by using the RunUMAP function on the first 6 PCs to visualize cell clusters. Doublets were removed using the DoubletFinder R package (McGinnis et al, 2019). Finally, the Differential Gene Expression analysis between clusters was performed by using the non-parametric Wilcoxon Rank Sum test through the FindAllMarkers function. A gene was defined as differentially expressed (DEGs) when the Bonferroni-adjusted *P* value  < 0.01 and the absolute value of avg_log2FC > 0.25.

Gene Ontology enrichment analysis (GOEA) was performed on induced and inhibited genes, separately, for each Cluster, by using the DAVID Bioinformatic tool (Huang et al, 2009a; Dennis et al, 2003) restricting the output to Cellular Compartments (CC) terms. The threshold for statistical significance of GOEA was FDR < 0.1 and Enrichment Score >1.5. The top 10 significant CCs for clusters 2 and 10 are shown in Fig. 5C.

To infer TFs from single-cell data, we used the pySCENIC tool (version 0.12.1) (Aibar et al, 2017). The pySCENIC output was integrated into the Scanpy object (Wolf et al, 2018), and the most active regulons were identified for cells belonging to clusters 2 and 10. Subsequently, using the FIMO program (Grant et al, 2011) from the MEME Suite, we searched for the most active TFs obtained from the pySCENIC analysis that bound to the promoters of the differentially expressed genes in clusters 2 and 10. TF matrix for each gene was selected when the *P* value < 0.05.

#### Data visualization

Heatmaps (Fig. 5C,E,F) were generated using the pheatmap package (Kolde, 2019) from R on the transformed expression data and setting scale = ‘row’ to scale the data to Z-scores (by gene). For the UMAP figures, the Seurat object was imported to Scanpy (Wolf et al, 2018) using Seurat’s Convert function. UMAPs (Fig. 5A,B) were plotted using seaborn.scatterplot (Waskom, 2021) function and gene expression distribution along the UMAP (Fig. 5E) was plotted using scanpy.pl.umap function (Wolf et al, 2018).

### Bulk RNA-seq

Bulk RNA-seq analysis was performed on two separate experiments: (1) HeLa cells were transfected with CREB3L1, CREB3L1-CA, MITF, SP1 cDNAs or a control plasmid; (2) HeLa cells underwent endosome inactivation by incubation with DAB and H_2_O_2_, followed by recovery periods of 6 h and 18 h, along with corresponding controls that were incubated with only H_2_O_2_.

#### Library preparation

Total mRNA was extracted from the cells and quantified using the Qubit 4.0 fluorometric Assay (Thermo Fisher Scientific). Libraries were prepared from 125 ng of total RNA using the NEGEDIA Digital mRNA-seq research grade sequencing service v2.0 (Next Generation Diagnostic srl) (Xiong et al, 2017), which included library preparation, quality assessment and sequencing on a NovaSeq 6000 sequencing system using a single-end, 100 cycle strategy (Illumina Inc.).

#### Bioinformatics workflow

The raw data were analyzed by Next Generation Diagnostic srl proprietary NEGEDIA Digital mRNA-seq pipeline (v2.0) which involves a cleaning step by quality filtering and trimming, alignment to the reference genome and counting by gene (Dobin et al, 2013; Anders et al, 2015). The raw expression data were normalized, analyzed by NEGEDIA degsanalysis pipeline (v1.2.0) (Love et al, 2014).

#### Functional analysis on transcriptomics data

The threshold for the statistical significance of gene expression was FDR < 0,05. To dissect which biological pathways are activated following the overexpression of the transcription factors (MITF, CREB3L1 and SP1), Gene Ontology enrichment analysis (GOEA) was performed on induced and inhibited genes, separately, by using the DAVID Bioinformatic tool (Huang et al, 2009b) restricting the output to Biological Process (BP), Cellular Compartments (CC) terms. The threshold for statistical significance of GOEA was FDR < 0,1 and Enrichment Score ≥1.5.

### Statistical analyses

Statistical analyses for both single-cell and bulk RNA-seq datasets were performed as detailed in the corresponding sections above. Analyses for other experiments utilized GraphPad Prism software. Unless otherwise noted: (i) all experiments were conducted a minimum of three times; (ii) figure data are presented as Mean ± SD; (iii) *n* denotes the number of biological replicates; (iv) each color in super-plots represents the mean value alongside individual values from a single replicate. Statistical significance was assessed using either Student’s *t* test or ANOVA (one-way or two-way). Time-series data of Golgi marker recovery (Fig. 2C) were subjected to hierarchical clustering (Fig. 2D) using by Orange Data Mining software (version 3.39.0). No explicit sample size calculation was carried out. Sample sizes were determined based on the observed consistency and statistical significance between groups or conditions. Specimens were blinded during microscopy analyses.

## Supplementary information

Appendix

Peer Review File

Dataset EV1

Dataset EV2

Dataset EV3

Dataset EV4

Dataset EV5

Dataset EV6

Movie EV1

Movie EV2

Movie EV3

Movie EV4

Movie EV5

Movie EV6

Movie EV7

Movie EV8

Movie EV9

Movie EV10

Movie EV11

Source data Fig. 1

Source data Fig. 2

Source data Fig. 3

Source data Fig. 4

Source data Fig. 5

Source data Fig. 6

Source data Fig. 7

Source data Fig. 8

Source data Fig. 9

Expanded View Figures

## Data Availability

scRNA-seq and bulk RNA-seq data have been deposited in the Gene Expression Omnibus (GEO) under accession codes: GSE288395, GSE299965, GSE325448. All other data are available in the manuscript and the corresponding Source Data files. Additional supporting datasets in their original formats have been deposited in the publicly accessible Zenodo repository (10.5281/zenodo.19550457). Newly generated tools (plasmids and cell lines) are available upon request under a material transfer agreement. The source data of this paper are collected in the following database record: biostudies:S-SCDT-10_1038-S44318-026-00828-7.
