# Supplementary material for: A coordinated transcriptional program controls de novo Golgi biogenesis
Source: EMBO J. 2026 Jun 6;45(16):5851–83. doi: 10.1038/s44318-026-00828-7 (PMC13483034; doi:10.1038/s44318-026-00828-7)
Supplement: Supplementary file 1 [file 44318_2026_828_MOESM10_ESM.zip › Movie EV10/Movie EV10 legend.docx]

**Movie EV10. Golgi recovery is impaired upon transcription inhibition.**

The Golgi complex was inactivated in ManII-HRP cells expressing GalT-GFP, followed by treatment with the transcription inhibitor actinomycin D (Act-D). These cells failed to recover the Golgi, as indicated by the absence of recovery of the compact GalT-GFP fluorescent signal in the perinuclear region. Note: the LUT was set to the global minimum and maximum of the signal within the sequence to visualize the minimal residual GalT-GFP fluorescence remaining after DAB quenching. The time is shown in hh:mm format.
