# Supplementary material for: A coordinated transcriptional program controls de novo Golgi biogenesis
Source: EMBO J. 2026 Jun 6;45(16):5851–83. doi: 10.1038/s44318-026-00828-7 (PMC13483034; doi:10.1038/s44318-026-00828-7)
Supplement: Supplementary file 2 [file 44318_2026_828_MOESM11_ESM.zip › Movie EV11/Movie EV11 legend.docx]

**Movie EV11. Golgi recovery is impaired upon cell enucleation.**

ManII-HRP cells expressing GalT-GFP were enucleated prior to inactivation of the pre-existing Golgi. Nucleus-free cytoplasts, which lack transcriptional activity, failed to rebuild the Golgi apparatus. Note: the LUT was set to the global minimum and maximum of the signal within the sequence to visualize the minimal residual GalT-GFP fluorescence after DAB quenching. A residual GalT-GFP signal is visible in one cytoplast (on the right), but it does not increase over time. The time is shown in hh:mm format.
