# Supplementary material for: A coordinated transcriptional program controls de novo Golgi biogenesis
Source: EMBO J. 2026 Jun 6;45(16):5851–83. doi: 10.1038/s44318-026-00828-7 (PMC13483034; doi:10.1038/s44318-026-00828-7)
Supplement: Supplementary file 3 [file 44318_2026_828_MOESM12_ESM.zip › Movie EV2/Movie EV2 legend.docx]

**Movie EV2. Rapid DAB accumulation in HeLa cells following Golgi cross-linking.**

The movie begins immediately after HeLa cells expressing ManII-HRP and GalT-GFP were incubated with diaminobenzidine (DAB) and hydrogen peroxide (H₂O₂) to cross-link the pre-existing Golgi. DAB accumulation within the Golgi region is observed within 30 seconds and is accompanied by quenching of GalT-GFP fluorescence.
