# Supplementary material for: A coordinated transcriptional program controls de novo Golgi biogenesis
Source: EMBO J. 2026 Jun 6;45(16):5851–83. doi: 10.1038/s44318-026-00828-7 (PMC13483034; doi:10.1038/s44318-026-00828-7)
Supplement: Supplementary file 4 [file 44318_2026_828_MOESM13_ESM.zip › Movie EV3/Movie EV3 legend.docx]

**Movie EV3. Golgi recovery after inactivation.**

The movie begins immediately after ManII-HRP HeLa cells expressing GalT-GFP were incubated with DAB and H₂O₂, resulting in quenching of the GalT-GFP signal in the pre-existing Golgi. Fluorescence reappears in the newly forming Golgi a few hours after inactivation and reaches a plateau at around 10–12 hours. The time is shown in hh:mm format.
