# Supplementary material for: A coordinated transcriptional program controls de novo Golgi biogenesis
Source: EMBO J. 2026 Jun 6;45(16):5851–83. doi: 10.1038/s44318-026-00828-7 (PMC13483034; doi:10.1038/s44318-026-00828-7)
Supplement: Supplementary file 5 [file 44318_2026_828_MOESM14_ESM.zip › Movie EV4/Movie EV4 legend.docx]

**Movie EV4. ER-Golgi trafficking of GPI-RUSH in control cells.**

This time-lapse sequence shows the early stages of trafficking of GPI-RUSH released from the ER in control ManII-HRP HeLa cells upon biotin addition. Bright-field images at the beginning confirm the absence of DAB deposition in the Golgi. GPI-RUSH concentrates in transport carriers, which appear as bright spots emerging from the ER and then moving toward the Golgi. The time is shown in hh:mm:ss format.
