# Supplementary material for: A coordinated transcriptional program controls de novo Golgi biogenesis
Source: EMBO J. 2026 Jun 6;45(16):5851–83. doi: 10.1038/s44318-026-00828-7 (PMC13483034; doi:10.1038/s44318-026-00828-7)
Supplement: Supplementary file 6 [file 44318_2026_828_MOESM15_ESM.zip › Movie EV5/Movie EV5 legend.docx]

**Movie EV5. ER-Golgi trafficking of GPI-RUSH during Golgi recovery after inactivation.**

This time-lapse sequence shows the early stages of trafficking of GPI-RUSH released from the ER in ManII-HRP HeLa cells treated with DAB to cross-link the Golgi. Bright-field images at the beginning confirm the presence of DAB deposition in the Golgi. ER-to-Golgi transport occurs similarly to that in control cells and is mediated by GPI-RUSH transport carriers with similar features and dynamics. The time is shown in hh:mm:ss format.
