# Supplementary material for: A coordinated transcriptional program controls de novo Golgi biogenesis
Source: EMBO J. 2026 Jun 6;45(16):5851–83. doi: 10.1038/s44318-026-00828-7 (PMC13483034; doi:10.1038/s44318-026-00828-7)
Supplement: Supplementary file 7 [file 44318_2026_828_MOESM16_ESM.zip › Movie EV6/Movie EV6 legend.docx]

**Movie EV6. Trafficking of GPI-RUSH in control cells.**

This time-lapse sequence shows trafficking of GPI-RUSH released from the ER in control ManII-HRP HeLa cells upon biotin addition. Following ER release, GPI-RUSH rapidly traffics to the Golgi and subsequently to the cell surface. The movie is a composite generated by combining two time-lapse recordings acquired with different imaging intervals. During the first 40 min after biotin addition, images were acquired with shorter time intervals to capture the details of rapid ER-to-Golgi transport. After cargo arrival at the Golgi, images were acquired with longer time intervals to minimize phototoxicity during prolonged imaging. The time is shown in hh:mm format.
