# Supplementary material for: A coordinated transcriptional program controls de novo Golgi biogenesis
Source: EMBO J. 2026 Jun 6;45(16):5851–83. doi: 10.1038/s44318-026-00828-7 (PMC13483034; doi:10.1038/s44318-026-00828-7)
Supplement: Supplementary file 8 [file 44318_2026_828_MOESM17_ESM.zip › Movie EV7/Movie EV7 legend.docx]

**Movie EV7. Trafficking of GPI-RUSH during Golgi recovery after inactivation.**

This time-lapse sequence shows trafficking of GPI-RUSH released from the ER in ManII-HRP HeLa cells treated with DAB to cross-link the Golgi. Biotin addition triggers GPI-RUSH trafficking immediately after Golgi inactivation. While ER-to-Golgi transport occurs at speeds comparable to control cells, subsequent export from the Golgi and delivery to the plasma membrane is markedly slowed in DAB-treated cells. The movie is a composite generated by combining two time-lapse recordings acquired with different imaging intervals. During the first 30 min after biotin addition, images were acquired with shorter time intervals to capture the details of rapid ER-to-Golgi transport. After cargo arrival at the Golgi, images were acquired with longer time intervals to minimize phototoxicity during prolonged imaging. The time is shown in hh:mm format.
