# Supplementary material for: A coordinated transcriptional program controls de novo Golgi biogenesis
Source: EMBO J. 2026 Jun 6;45(16):5851–83. doi: 10.1038/s44318-026-00828-7 (PMC13483034; doi:10.1038/s44318-026-00828-7)
Supplement: Supplementary file 9 [file 44318_2026_828_MOESM18_ESM.zip › Movie EV8/Movie EV8 legend.docx]

**Movie EV8. Trafficking of M6PR-RUSH in control cells.**

This movie shows trafficking of M6PR-RUSH released from the ER in control ManII-HRP HeLa cells. Following ER release, M6PR-RUSH (red) rapidly traffics to the Golgi, highlighted by GalT-GFP (green), and then moves from the Golgi to scattered post-Golgi endosomal structures. The time is shown in hh:mm format.
