# Supplementary material for: A coordinated transcriptional program controls de novo Golgi biogenesis
Source: EMBO J. 2026 Jun 6;45(16):5851–83. doi: 10.1038/s44318-026-00828-7 (PMC13483034; doi:10.1038/s44318-026-00828-7)
Supplement: Supplementary file 10 [file 44318_2026_828_MOESM19_ESM.zip › Movie EV9/Movie EV9 legend.docx]

**Movie EV9. Trafficking of M6PR-RUSH during Golgi recovery after inactivation.**

This movie shows trafficking of M6PR-RUSH released from the ER in ManII-HRP HeLa cells treated with DAB to cross-link the Golgi. Biotin addition initiates M6PR-RUSH trafficking immediately after Golgi inactivation (DAB deposition is visible in the bright-field panel). After ER exit, M6PR-RUSH (red) gradually accumulates within the newly forming Golgi (green); however, its export to endosomal compartments is substantially delayed compared to control cells. The time is shown in hh:mm format.
