# Supplementary material for: A coordinated transcriptional program controls de novo Golgi biogenesis
Source: EMBO J. 2026 Jun 6;45(16):5851–83. doi: 10.1038/s44318-026-00828-7 (PMC13483034; doi:10.1038/s44318-026-00828-7)
Supplement: Supplementary file 20 [file 44318_2026_828_MOESM9_ESM.zip › Movie EV1/Movie EV1 legend.docx]

**Movie EV1. EM tomography of newly forming Golgi.**

This movie shows the three-dimensional ultrastructure of the newly forming Golgi two hours after inactivation of the pre-existing organelle. Arrows indicate COP-II buds at ER exit sites flanking the cluster of Golgi membranes, which comprises vacuolar elements as well as swollen and flat cisternae.
