# Supplementary material for: A coordinated transcriptional program controls de novo Golgi biogenesis
Source: EMBO J. 2026 Jun 6;45(16):5851–83. doi: 10.1038/s44318-026-00828-7 (PMC13483034; doi:10.1038/s44318-026-00828-7)
Supplement: Supplementary file 1 [file 44318_2026_828_MOESM1_ESM.pdf]

Appendix Materials for

De novo Golgi biogenesis requires coordinated activation of a Golgi regulon

CONTENTS:

Appendix Figures S1-S5 (pages 2-6)
Appendix Supplementary Methods (page 7)
Appendix Tables S1, 2 (pages 8-9)

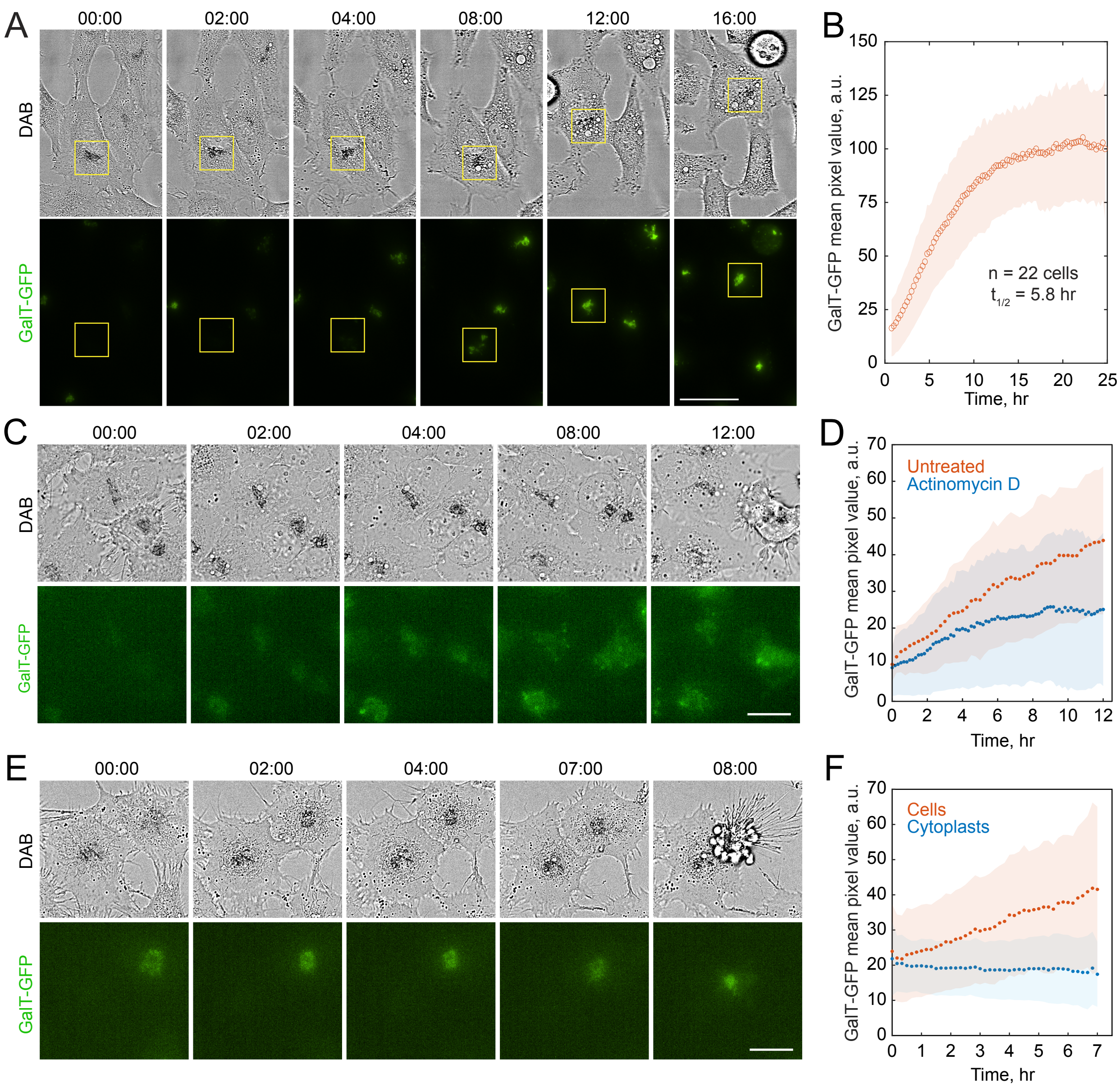

Appendix Figure S1. Live cell imaging of Golgi recovery.

A. Time frames extracted from a time-lapse sequence showing Golgi recovery in ManII-HRP HeLa cells expressing GalT-GFP. Immediately after incubation with DAB and H_2O_2 (time 0), the cells display a DAB signal and no GalT-GFP fluorescence, indicating inactivation of the Golgi. The cells were then incubated with fresh medium to allow Golgi reconstitution. The square outlines the perinuclear area of the cell, which gradually forms new GalT-GFP–positive Golgi structures, while the old DAB-positive Golgi undergoes fragmentation (see Movie 3). **B.** Quantification of GalT-GFP signal over time. The fluorescence intensity reaches a plateau approximately 10–12 hours post-inactivation. **C, D.** Cells treated with DAB as in panel A were then incubated with medium containing Actinomycin D. Time-lapse imaging (C; see Movie 10) shows a significant impairment in new Golgi formation, as confirmed by quantification of GalT-GFP fluorescence (D). **E, F.** Cytoplasts were obtained by enucleation of cells expressing ManII-HRP and GalT-GFP (see Methods). After Golgi inactivation with DAB, cytoplasts fail to recover GalT-GFP signal (E; see Movie 11), as confirmed by quantification of GalT-GFP fluorescence (F). **Scale bars:** 40 μm (A), 20 μm (C, E).

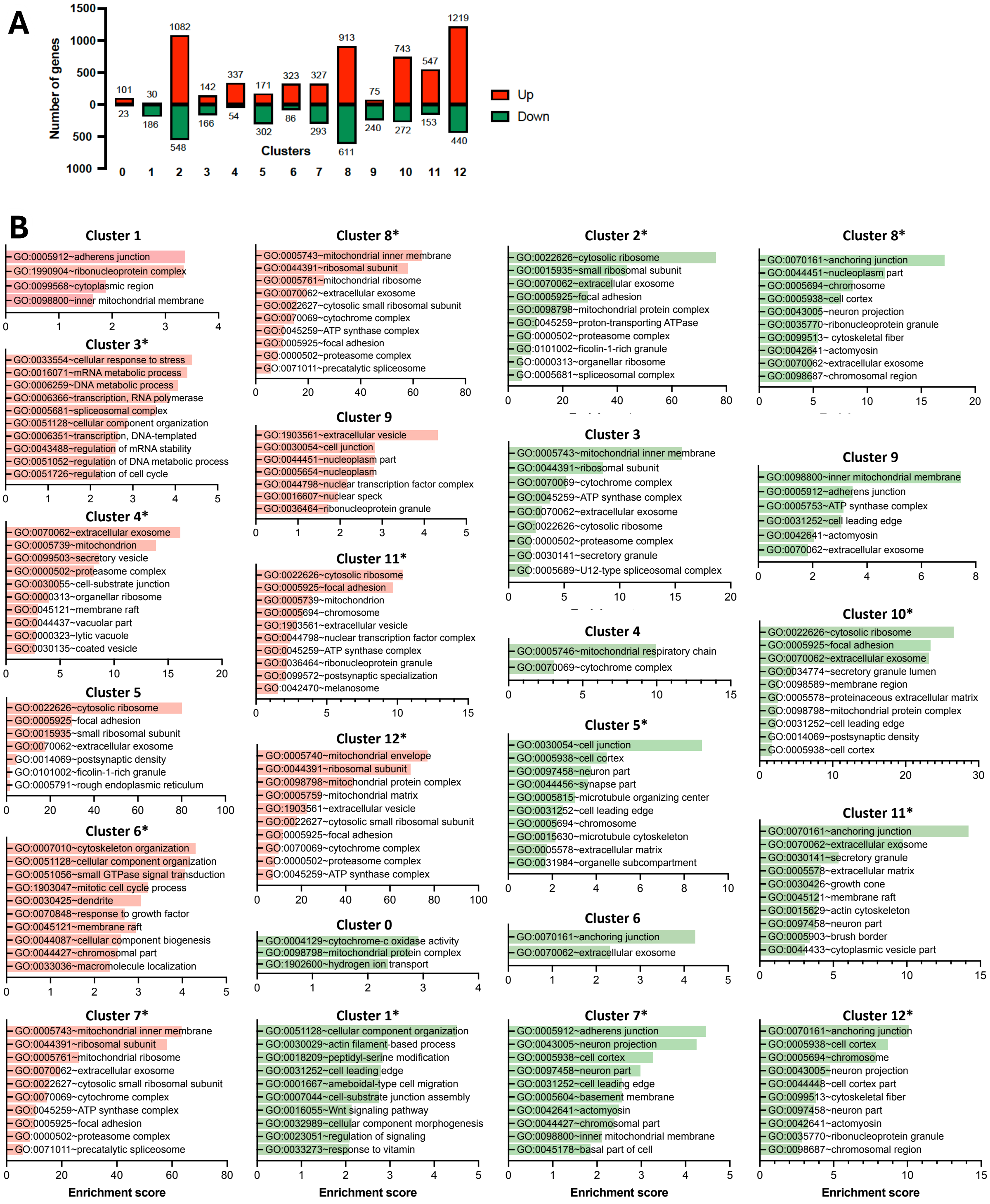

Appendix Figure S2. Gene ontology (GO) enrichment analysis of differently expressed genes (DEGs) in each cluster of scRNA-sec dataset.

DEGs were identified (as described in Material and Methods) in each cluster of cells (see Fig 5A), which were subjected to Golgi inactivation and subsequent RNA-seq analysis. **A.** The graph shows number of DEGs for each cluster. **B.** Differently expressed genes in each cluster were subjected to GO enrichment analysis. The graphs show enriched annotation terms for up- (red) or down-regulated (green) transcripts in each cluster. Enrichment scores for upregulated genes in clusters 2 and 10 are shown in main Fig 5C. No significant enrichments were detected for upregulated genes in cluster 0. Asterisk indicate clusters for which only top 10 terms are shown.

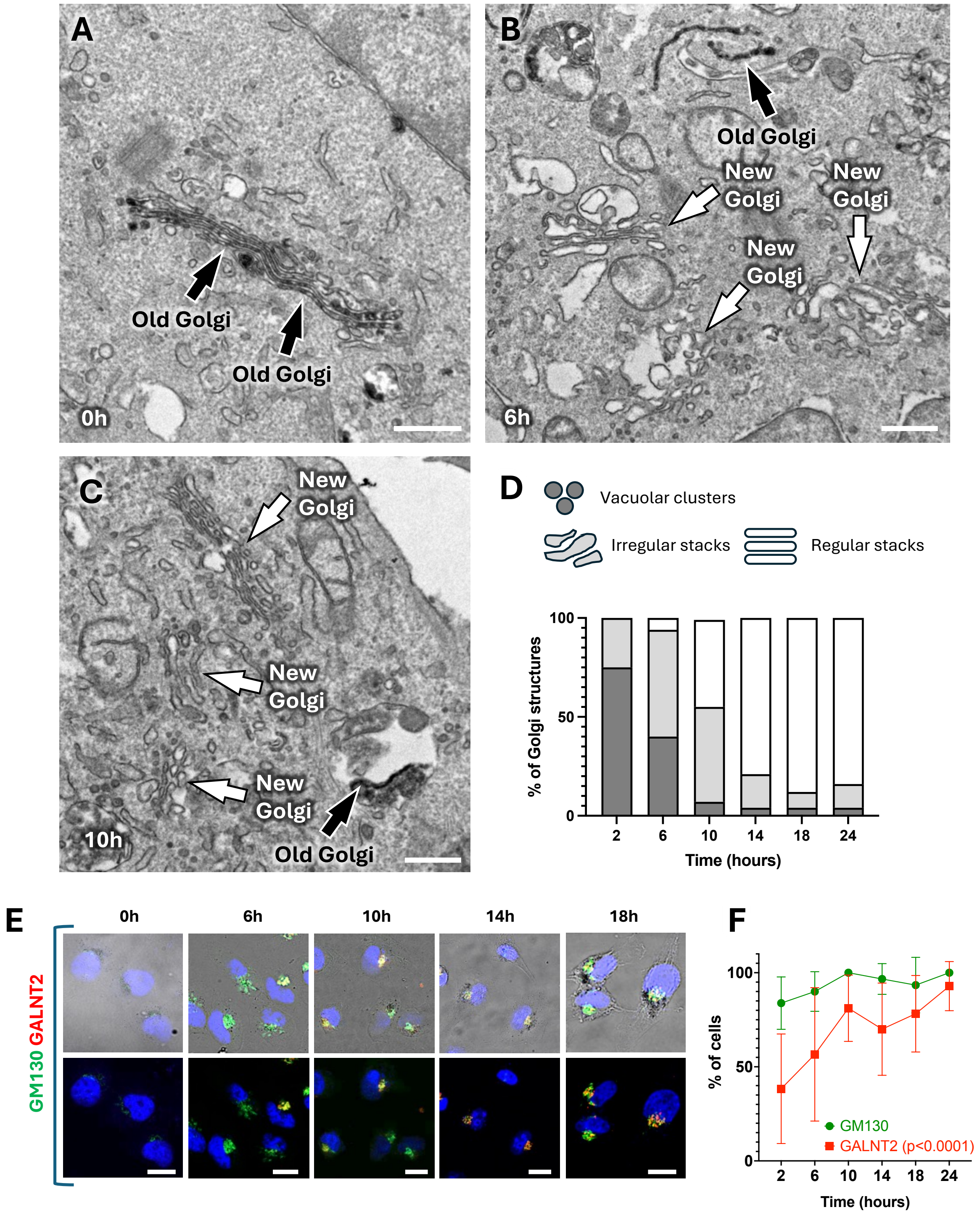

Appendix Figure S3. Golgi biogenesis in cells processed in parallel with scRNAseq experiment.
ManII-HRP-expressing HeLa cells were incubated with DAB and H_2O_2 to cross-link the preexisting (old) Golgi. The cells were collected immediately after Golgi inactivation or at 2, 6, 10, 14, 18, and 24-hour intervals of incubation with fresh medium. At each time point, the collected cells were processed for scRNAseq (see Figures 5 and 6), EM (A–D), and IF (E, F) analyses. **A–D.** EM images (A–C) and quantification (D) show that a substantial proportion of newly formed Golgi units acquire regular stack architecture starting from 10 hours post-inactivation. Quantification in D was done from a single biological replicate. **E, F.** Confocal images (E) and quantification (F) show that the majority of cells recover GALNT2 in the newly forming Golgi units at the 10-hour interval after inactivation of the old Golgi ($p < 0.0001$, two-way ANOVA; n = 6 view fields, single biological replicate). Scale bars: 360 nm (A–C), 17 µm (E).

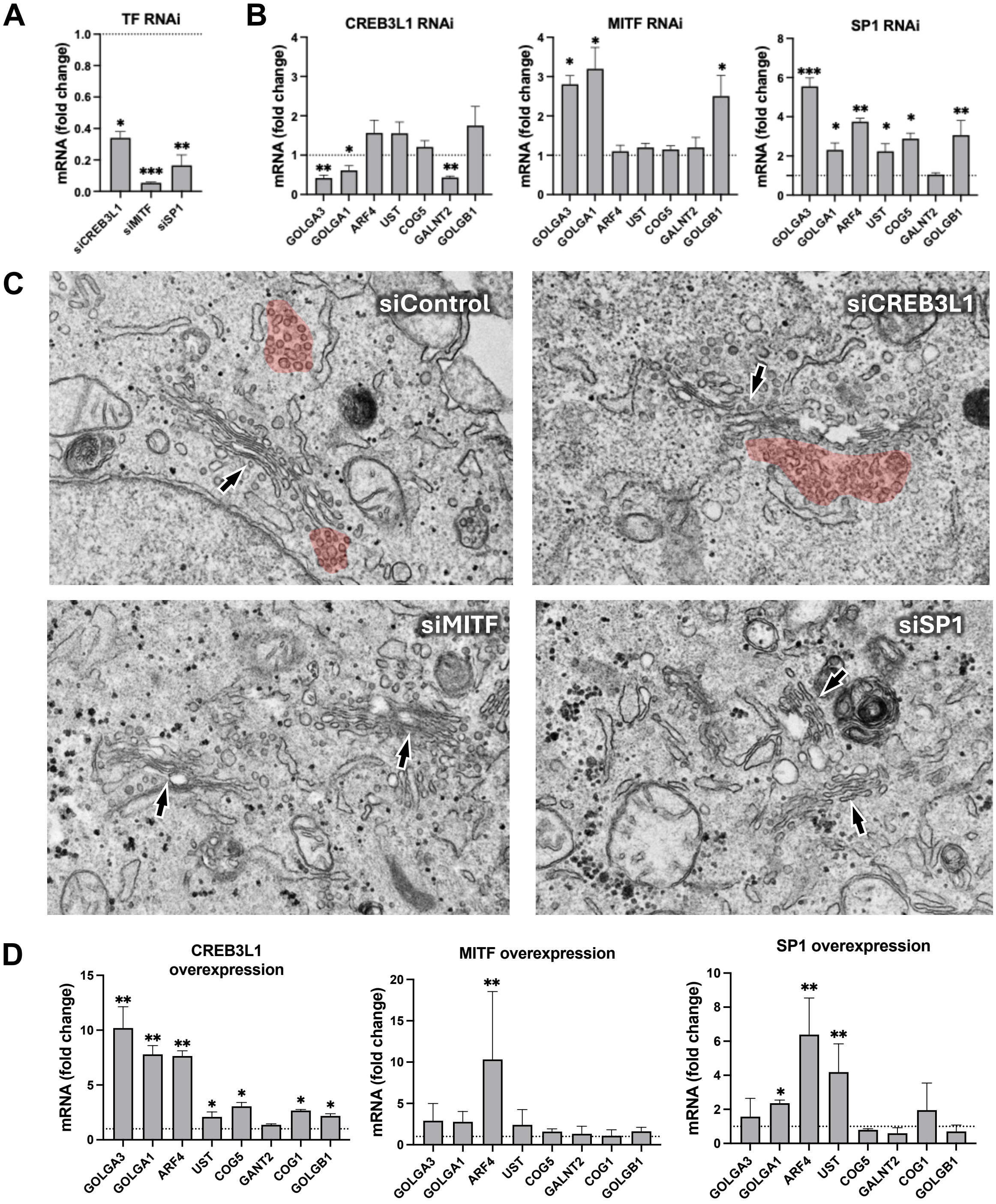

Appendix Figure S4. Impact of silencing and overexpression of TF candidates.

A, B. CREB3L1, MITF, or SP1 were silenced in HeLa cells, and changes in TFs' (A) or Golgi gene (B) expression were evaluated by qRT-PCR relative to control cells (dashed line). Statistical significance: ***p<0.001, **p<0.01, *p<0.05 (one-way ANOVA; n=3 experiments). **C.** Golgi ultrastructure was assessed by electron microscopy (EM) in silenced cells. Arrows indicate Golgi stacks. Red highlights pre-Golgi vesicular-tubular clusters (VTCs), indicating significant VTC expansion in CREB3L1-silenced cells. **D.** CREB3L1, MITF, or SP1 were overexpressed in HeLa cells, and changes in Golgi gene expression were evaluated by qRT-PCR relative to control cells (dashed line). Statistical significance: **p<0.01, *p<0.05 (one-way ANOVA; n=3 experiments).

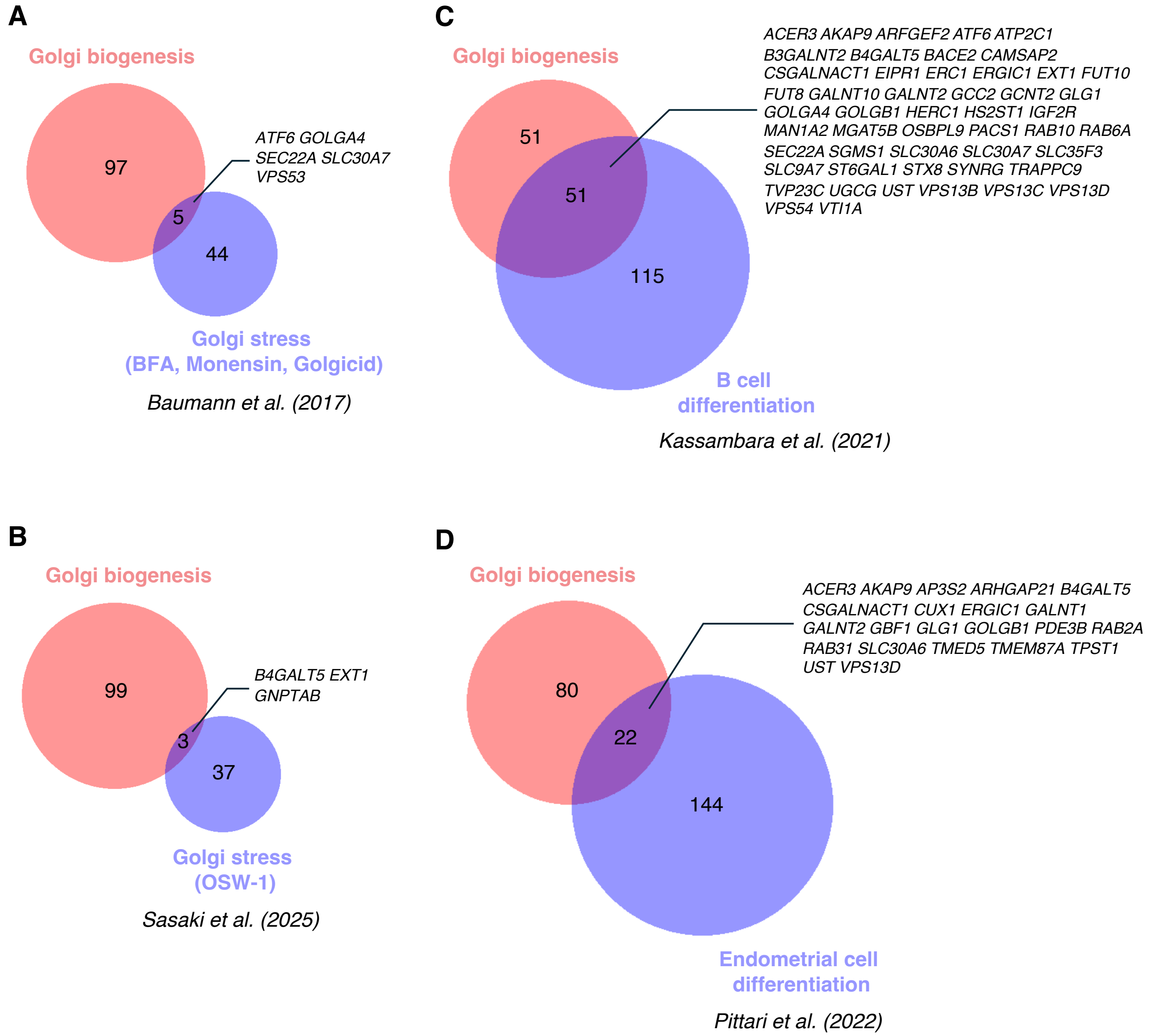

Appendix Figure S5. Comparison of Golgi gene sets activated during Golgi regeneration, Golgi stress, or cell differentiation.

VENN diagrams show the overlap between set of Golgi genes activated during Golgi biogenesis (this study) and Golgi gene sets reported to be induced by Golgi stress (A, B) or during cell differentiation (C, D). For each panel, the number of overlapping and non-overlapping genes is indicated. Common gene names are listed, and the source of each compared gene set is referenced.

APPENDIX MATERIALS AND METHODS

DAB-Mediated Golgi Inactivation

The Golgi apparatus was inactivated by inducing DAB polymer deposition within Golgi cisternae in HeLa cells stably expressing ManII-HRP.

1) Prepare the 10x stock solution, dissolve 50 mg of 3,3′-diaminobenzidine (DAB; D8001, Sigma-Aldrich) powder in 10 mL of HCl 0.1 M. Prepare several aliquots of the stock solution and store them at -80°C.
2) Incubate ManII-HRP expressing cells (70-80% confluency) at room temperature with the DAB solution (1x diluted DAB stock solution in 0,1 M Tris-HCl pH 7.4 + 0,02% H_2O_2).
3) Monitor under the bright-field microscope the DAB-treated cells, stopping the reaction as a light brown coloration appears in the perinuclear area of the cells, indicating DAB deposition within the Golgi.
4) Wash the cells with PBS to remove the excess DAB.
5) Collect the cells immediately or re-incubate them in conditioned medium to assess the Golgi recovery using methods outlined below.

CATCHFIRE-Based Retrograde Golgi-to-ER Trafficking Assay

The CATCHFIRE system utilizes a fluorogenic proximity-control method founded on chemically induced dimerization, wherein two engineered components—FIREMate and FIREtag—selectively interact upon addition of a small-molecule ligand termed “match”. In this investigation, ManII-HRP HeLa cells were transfected with a construct encoding FIREMate-KDEL and ManII-mApple-FIREtag. To monitor retrograde trafficking, FIREMate-KDEL served as an ER-localized anchor to capture the ManII-mApple-FIREtag reporter as it recycles from the Golgi apparatus.

1) Transfect ManII-HRP HeLa cells using Lipofectamine 3000 according to the manufacturer’s instructions with a plasmid encoding FIREMate–KDEL and ManII–mApple–FIREtag.

2) Immediately after transfection, supplement the culture medium with “match” at a final concentration of 10 µM to accumulate ManII–mApple–FIREtag in the ER.

3) Maintain cells continuously in match-containing medium from the time of transfection until the experiment.

4) Conduct Golgi inactivation (see above).

5) Perform “match” washout to allow ManII–mApple–FIREtag delivery to the Golgi. Remove the match-containing medium, wash cells three times with PBS, and replace with match-free medium for 1 hour.

6) Reintroduce match for 1 and 2 hour intervals to promote ManII–mApple–FIREtag capture by FIREMate–KDEL in the ER.

7) Analyze the cells by confocal microscopy to assess the efficiency of ManII–mApple–FIREtag delivery from the Golgi to the ER.

8) Perform the same treatment without subjecting cells to Golgi inactivation and use these as controls.

Appendix Table S1. List of the siRNAs used in the study

Target gene	Forward Strand	Reverse Strand
**Human**		
NR3C1 siRNA1	CACUCUCAAUGGGACUGUATT	TTUACAGUCCCAUUGAGAGUG
NR3C1 siRNA2	GCAGGAUCAGAAGCCUAUUTT	TTAAUAGGCUUCUGAUCCUGC
MLLT10 siRNA1	GAACGGCUUCAGUUAUUGATT	TTUCAAUAACUGAAGCCGUUC
MLLT10 siRNA2	GCAGCUUACAGAUUCGCUATT	TTUAGCGAAUCUGUAAGCUGC
HDAC1 siRNA1	CCGGUCAUGUCCAAAGUAATT	TTUUACUUUGGACAUGACCGG
HDAC1 siRNA2	CCAAUAUGACUAACCAGAATT	TTUUCUGGUUAGUCAUAUUGG
CUX1 siRNA1	GAAAGCGGCUUAUCGAACATT	TTUGUUCGAUAAGCCGCUUUC
CUX1 siRNA2	GCAUAAGGUUCAGAGCCUATT	TTUAGGCUCUGAACCUUAUGC
GATAD2A siRNA1	AACUCGUGUUGUUGAAAAATT	TTUUUUUCAACAACACGAGUU
GATAD2A siRNA2	CGUUCAGUCCGUCACCCAATT	TTUUGGGUGACGGACUGAACG
CREB1 siRNA1	GGCUAACAAUGGUACCGAUTT	TTAUCGGUACCAUUGUUAGCC
CREB1 siRNA2	CAUUAGCCCAGGUAUCUAUTT	TTAUAGAUACCUGGGCUAAUG
CREB3 siRNA1	GAGUGAGAGCUGUAGAAAATT	TTUUUUCUACAGCUCUCACUC
CREB3 siRNA2	GGCUAGUACUGACAGAUGATT	TTUCAUCUGUCAGUACUAGCC
CREB3L3 siRNA1	UGAAGCCUCUGUGACCAUATT	TTUAUGGUCACAGAGGCUUCA
CREB3L3 siRNA2	CGAGGAGCGAGUGCUGAAATT	TTUUUCAGCACUCGCUCCUCG
CREB3L4 siRNA1	CCACAAGGACGUAACAGAATT	TTUUCUGUUACGUCCUUGUGG
CREB3L4 siRNA2	GAAGAUUUCUUGAAGCUUUTT	TTAAAGCUUCAAGAAAUCUUC
CREB3L1 siRNA1	UCCAGACUCUGGUCACCAATT	TTUUGGUGACCAGAGUCUGGA
CREB3L1 siRNA2	GGUGGAGACAUUUACAUCUTT	TTAGAUGUAAAUGUCUCCACC
CREB3L2 siRNA1	GAAUGAUCCUUUCCUCUCATT	TTUGAGAGGAAAGGAUCAUUC
CREB3L2 siRNA2	GAAGAUCAAGAAUAAGAUUTT	TTAAUCUUAUUCUUGAUCUUC
SP1 siRNA1	GGCAGACCUUUACAACUCATT	
SP1 siRNA2	CCACAAGCCCAAACAAUCATT	
SP1 siRNA3	GCAACAUGGGAAUUAUGAATT	
MITF siRNA1	CCGUGGACUAUAUCCGAAA	
MITF siRNA2	GAAACUUGAUUGAUCUUUA	
MITF siRNA3	GUACCUUUCUACCACUUUA	
YY1 siRNA1	AGCUUUUGUUGAGAGUUCATT	
YY1 siRNA2	AGAACUCACCUCCUGAUUATT	
YY1 siRNA3	CAGAAUAUAUGACAGGAAATT	

Appendix table S2. List of primers used in the study

Human gene	Forward	Reverse
NR3C1	ATAGCTCTGTTCCAGACTCAAC	TCCTGAAACCTGGTATTGCCT
MLLT10	TTTCCATTGCGTGTGTTGTT	CCATTGCTTTTCTGTGCTGA
HDAC1	CCGCATGACTCATAATTTGCTG	ATTGGCTTTGTGAGGGCGATA
CUX1	GAAGAACCAAGCCGAAACCAT	AGGCTCTGAACCTTATGCTCA
GATAD2A	CTCGTGTTGTTGAAAAAGTTGCG	GGTCTGGAGTGATGGCTTGC
CREB1	TTAACCATGACCAATGCAGCA	TGGTATGTTTGTACGTCTCCAGA
CREB3	ATGGAGCTGGAATTGGATGCT	TACTTCCCAGTCGCTCGGT
CREB3L3	ATGAATACGGATTTAGCTGCTGG	AGGAAGTCGTCAGAGTCGGG
CREB3L4	CAGACGCTAATTGCTCAAACTTC	CCACTTGGGTCTCCAGATTTTCT
CREB3L1	CCTCCCGAAGCCTCCTATTCT	GGGGTTGATTTCCCAGCCA
CREB3L2	CAGAGAAGAGTGTGTCAATGGAG	CTGGTGGTAATGTGGGTGAAG
MITF	GGGCTTGATGGATCCTGCTT	GCTGGACAGGAGTTGCTGAT
SP1	TCCAGACCATTAACCTCAGTGC	TGTATTCCATCACCACCAGCC
ACTB	AAGAGCTACGAGCTGCCTGA	GACTCCATGCCCAGGAAGG
ARF4	TGCAAACAAACAGGATTTGCCA	ACCAGTTCCTTGTGTTGCAC
COG1	CAAGCCAGACTCCAGAATTG	CGATGAAGGTTGCTGTTGAG
COG5	GCAAGTGGAACTACAGACCTCTA	GCAGCCTCATAGGGTTGTAGTG
GALNT2	AAGGAGAAGTCGGTGAAGCA	TCCCATTTCTGTCTGCTGTCA
GOLGA3	AGAAGGTGCTGGAGCTGGA	ACTGACCGAGGCCCGTAA
GOLGB1	TAAGCCAGATCCTGTGTTGC	GACCGTGGGATCCTAGTAGC
UST	GGCGTGCTCAGTATCTACAA	TCGTATCTCATCCGCTGGTA
