# Supplementary material for: A coordinated transcriptional program controls de novo Golgi biogenesis
Source: EMBO J. 2026 Jun 6;45(16):5851–83. doi: 10.1038/s44318-026-00828-7 (PMC13483034; doi:10.1038/s44318-026-00828-7)
Supplement: Supplementary file 2 [file 44318_2026_828_MOESM29_ESM.pdf]

Expanded View Figures

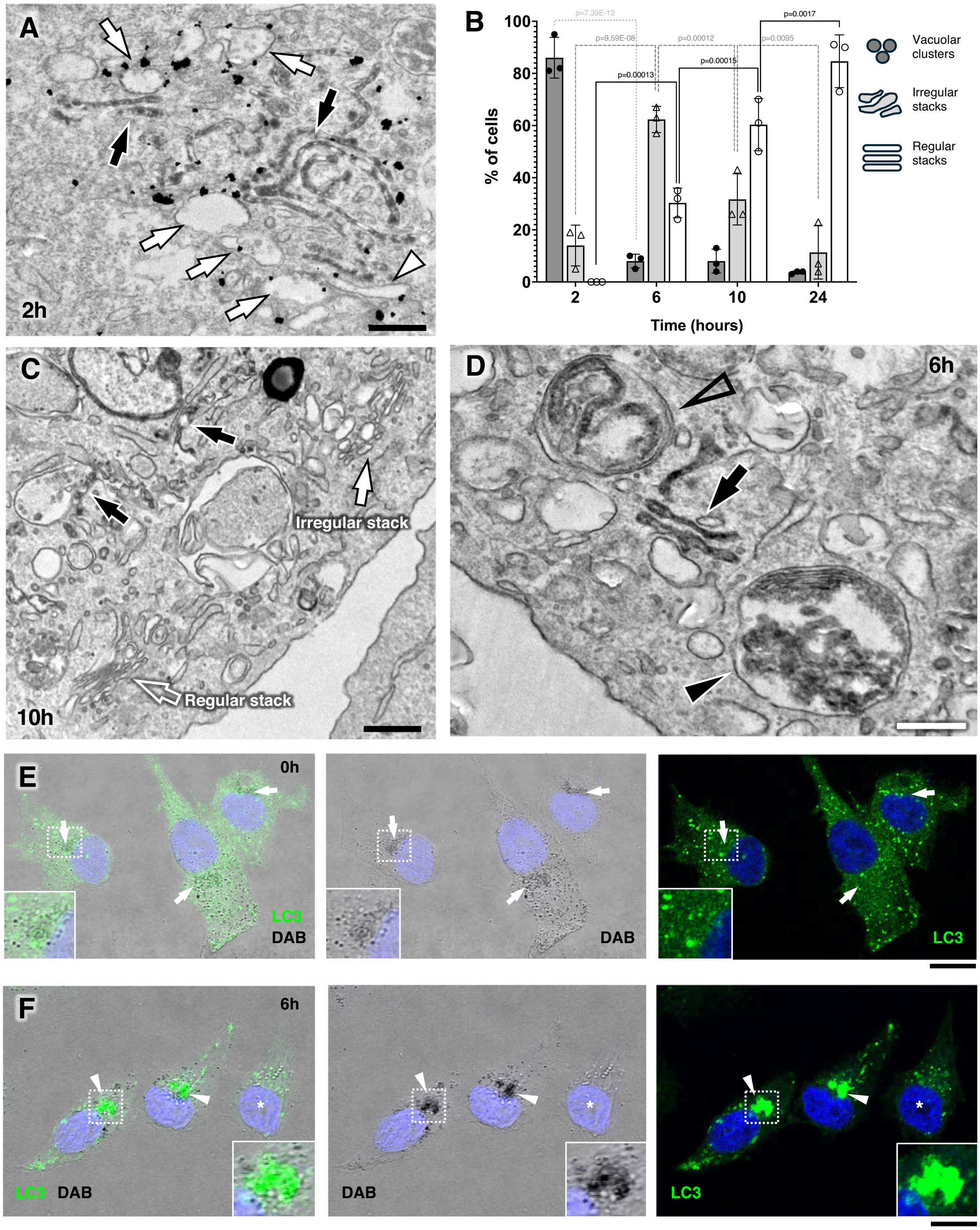

Figure EV1. Morphodynamics of newly forming Golgi and inactivated Golgi structures in ManII-HRP-expressing cells.

(**A**) Immuno-EM shows GM130-associated gold particles at the newly forming Golgi (white arrows) 2 h after inactivation of the old Golgi (black arrows). (**B**) Morphometric analysis shows cell fractions with different phenotypes of new Golgi at different time points post-inactivation (mean ± SD, two-way ANOVA; $n = 3$ replicates). (**C**) White and empty arrows show different phenotypes of the new Golgi within the same cell 10 h after inactivation of the old Golgi (black arrows). (**D**) DAB-positive remnants of the old Golgi were detected in the cytosol (arrow), in the autophagosome (empty arrowhead) and autolysosome (arrowhead). (**E**, **F**) Representative images show that inactivated DAB-positive Golgi (**E**, arrowheads) recruits LC3 by 6 h post-inactivation (**F**, arrowheads). Asterisk indicates DAB-negative cell without crosslinked Golgi. Scale bars: 320 nm (**A**, **C**, **D**), 18 μm (**E**, **F**).

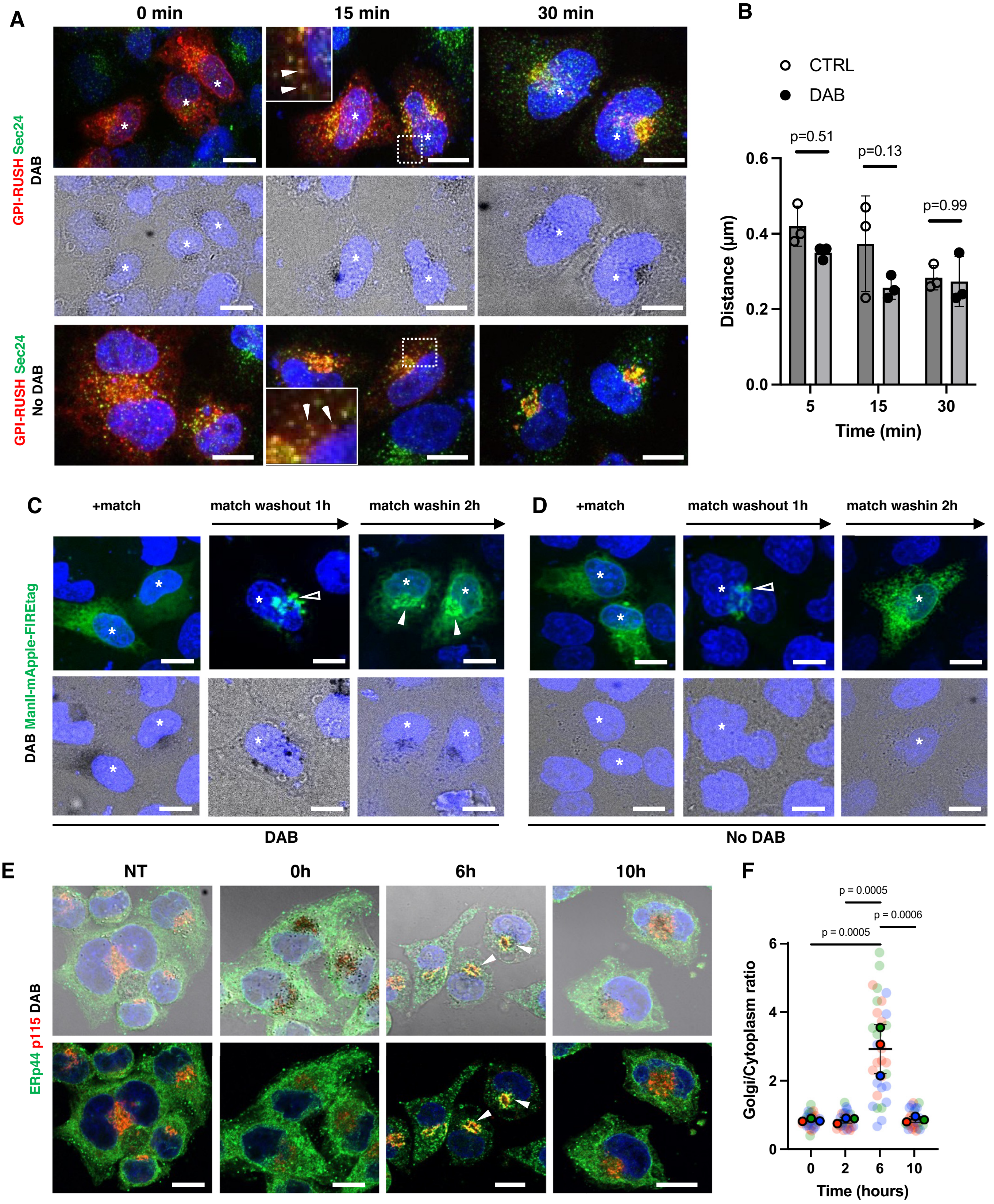
A
0 min
15 min
30 min
GPI-RUSH Sec24 DAB
GPI-RUSH Sec24 No DAB
B
CTRL
DAB
p=0.51
p=0.13
p=0.99
Distance (µm)
0.6
0.4
0.2
0.0
5
15
30
Time (min)
C
+match
match washout 1h
match washin 2h
D
+match
match washout 1h
match washin 2h
DAB ManII-mApple-FIREtag
DAB
No DAB
E
NT
0h
6h
10h
ERp44 p115 DAB
F
p = 0.0005
p = 0.0006
p = 0.0005
Golgi/Cytoplasm ratio
6
4
2
0
0
2
6
10
Time (hours)

Figure EV2. Trafficking at the ER/Golgi interface after Golgi inactivation in ManII-HRP-expressing HeLa cells.

(**A**) GPI-RUSH trafficking was activated by biotin in Golgi-inactivated (DAB) and control (No DAB) cells, with GPI-RUSH localizing to Sec24-positive spots (arrowheads). Asterisks indicate GPI-RUSH-expressing cells. (**B**) Coincidence between GPI-RUSH and Sec24 spots remained similar in both conditions (mean ± SD, two-way ANOVA, $n = 3$ replicates). (**C**, **D**) ManII-HRP cells expressing FIREMate-KDEL and ManII-mApple-FIREtag (asterisks) were treated with match to block ER exit of ManII-mApple-FIREtag reporter (see "Methods"). After Golgi inactivation, match was removed in DAB-treated and control cells to allow Golgi delivery (**C**, **D**, empty arrowheads) and then re-added to capture recycling to the ER (**D**). ManII-mApple-FIREtag was retained in the newly forming Golgi (**C**, arrowheads) but efficiently recycled to the ER in controls. (**E**) ERp44 showed ER-like distribution but accumulated in the Golgi 6 h post-inactivation. (**F**) Quantification confirmed significant Golgi retention of ERp44 at 6 h (mean ± SD; one-way ANOVA; $n = 3$ replicates). Scale bars: 16 µm (**A**, **C**–**E**).

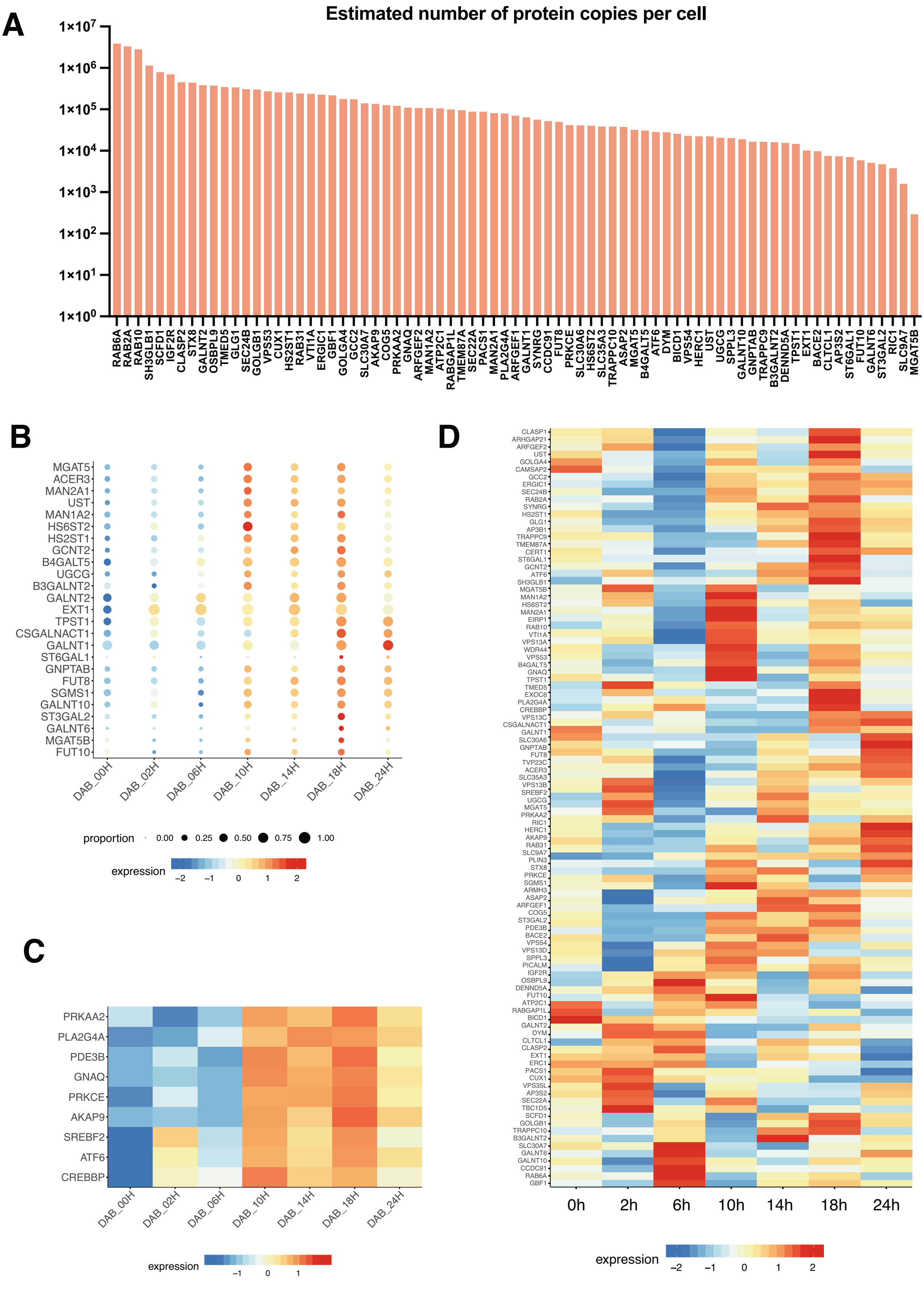
A
Estimated number of protein copies per cell
1×10^7
1×10^6
1×10^5
1×10^4
1×10^3
1×10^2
1×10^1
1×10^0
RAB6A RAB2A RAB10 SH3GLB1 SCFD1 IGF2R CLASP2 STX8 GALNT2 OSBPL9 TMED5 GLG1 SEC24B GOLGB1 VPS53 CUX1 HS2ST1 RAB31 VTI1A ERGIC1 GBF1 GOLGA4 GCC2 SLC30A7 AKAP9 COG5 PRKAA2 GNAQ ARFGEF2 MAN1A2 ATP2C1 RABGAP1L TMEM87A SEC22A PACS1 MAN2A1 PLA2G4A ARFGEF1 GALNT1 SYNRG CCDC91 FUT8 PRKCE SLC30A6 HS6ST2 SLC35A3 TRAPPC10 ASAP2 MGAT5 B4GALT5 ATF6 DYM BICD1 VPS54 HERC1 UST UGCG SPPL3 GALNT10 GNPTAB TRAPPC9 B3GALNT2 DENND5A TPST1 EXT1 BACE2 CLTCL1 AP3S2 ST6GAL1 FUT10 GALNT6 ST3GAL2 RIC1 SLC9A7 MGAT5B
B
MGAT5 ACER3 MAN2A1 UST MAN1A2 HS6ST2 HS2ST1 GCNT2 B4GALT5 UGCG B3GALNT2 GALNT2 EXT1 TPST1 CSGALNACT1 GALNT1 ST6GAL1 GNPTAB FUT8 SGMS1 GALNT10 ST3GAL2 GALNT6 MGAT5B FUT10
DAB_00H DAB_02H DAB_06H DAB_10H DAB_14H DAB_18H DAB_24H
proportion 0.00 0.25 0.50 0.75 1.00
expression −2 −1 0 1 2
C
PRKAA2 PLA2G4A PDE3B GNAQ PRKCE AKAP9 SREBF2 ATF6 CREBBP
DAB_00H DAB_02H DAB_06H DAB_10H DAB_14H DAB_18H DAB_24H
expression −1 0 1
D
CLASP1 ARHGAP21 ARFGEF2 UST GOLGA4 CAMSAP2 GCC2 ERGIC1 SEC24B RAB2A SYNRG HS2ST1 GLG1 AP3B1 TRAPPC9 TMEM87A CERT1 ST6GAL1 GCNT2 ATF6 SH3GLB1 MGAT5B MAN1A2 HS6ST2 MAN2A1 EIRP1 RAB10 VTI1A VPS13A WDR44 VPS53 B4GALT5 GNAQ TPST1 TMED5 EXOC6 PLA2G4A CREBBP VPS13C CSGALNACT1 GALNT1 SLC30A6 GNPTAB FUT8 TVP23C ACER3 SLC35A3 VPS13B SREBF2 UGCG MGAT5 PRKAA2 RIC1 HERC1 AKAP9 RAB31 SLC9A7 PLIN3 STX8 PRKCE SGMS1 ARMH3 ASAP2 ARFGEF1 COG5 ST3GAL2 PDE3B BACE2 VPS54 VPS13D SPPL3 PICALM IGF2R OSBPL9 DENND5A FUT10 ATP2C1 RABGAP1L BICD1 GALNT2 DYM CLTCL1 CLASP2 EXT1 ERC1 PACS1 CUX1 VPS35L AP3S2 SEC22A TBC1D5 SCFD1 GOLGB1 TRAPPC10 B3GALNT2 SLC30A7 GALNT6 GALNT10 CCDC91 RAB6A GBF1
0h 2h 6h 10h 14h 18h 24h
expression −2 −1 0 1 2

 © The Author(s)

Figure EV3. Specific features of activated Golgi genes.

(**A**) The estimated numbers of copies of Golgi proteins per cell were recovered from published proteomics data (Fasimoye et al, 2023) and plotted for corresponding genes, whose activation was observed during biogenesis of the new Golgi. (**B**) The bubble plot shows a subset of the Golgi genes (encoding glycosylation enzymes). Both the expression levels and number of cells expressing these genes increase with after inactivation of the old Golgi, especially at 10 h timepoint. (**C**) The heatmap shows expression dynamics of genes encoding Golgi-associated signaling proteins. Transcription factor associated genes ATF6, CREBBP, and SREBF2 exhibit earlier transactivation and are clustered together. (**D**) The expression of the Golgi genes (same as in Fig. 6C) was evaluated at different time points after incubation with DAB in cells without detectable ManII-HRP expression (83,000 cells in total). Majority of the genes exhibit fluctuating expression without specific activation time point.

© The Author(s)

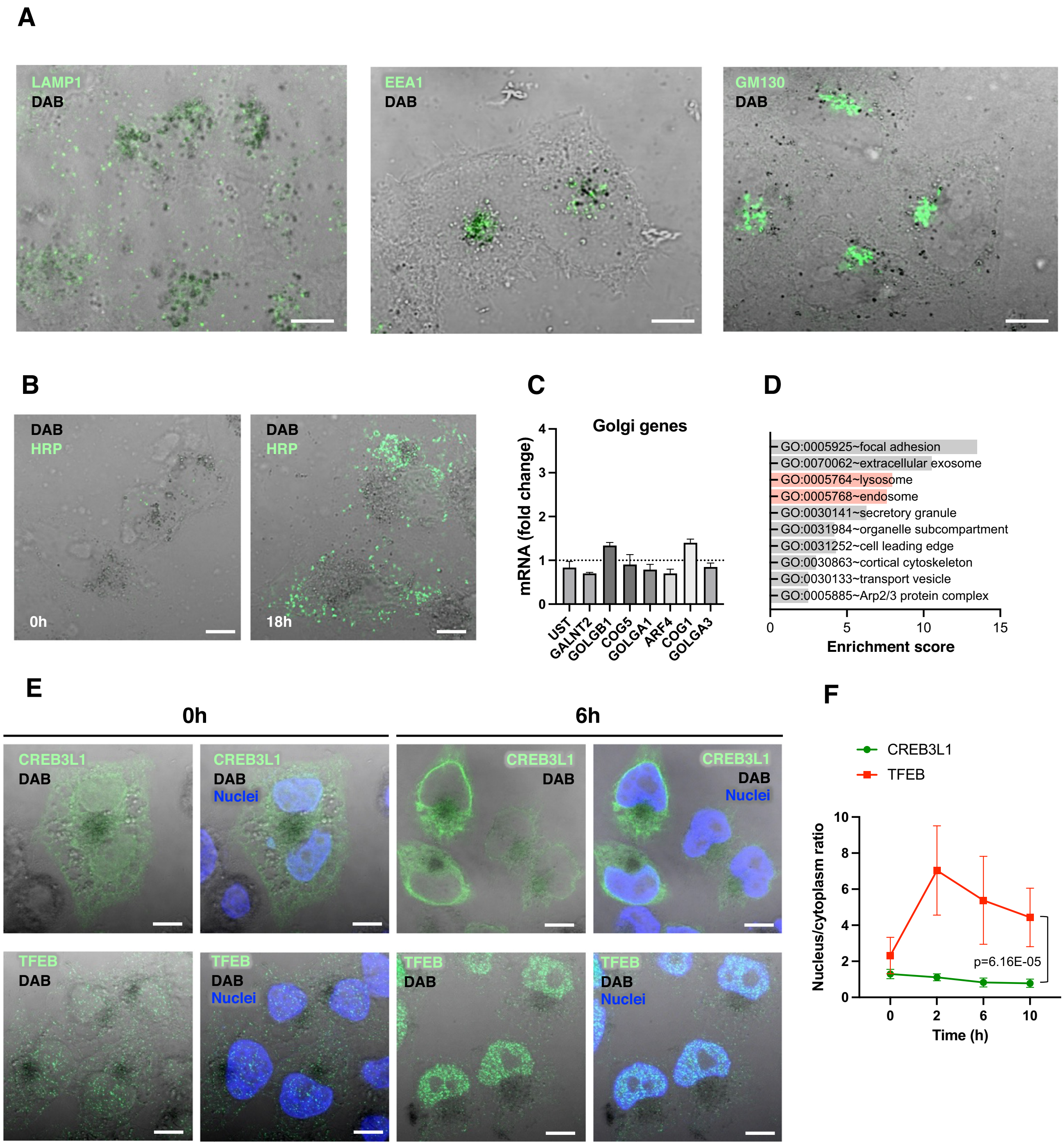
A
LAMP1
DAB
EEA1
DAB
GM130
DAB
B
DAB
HRP
0h
DAB
HRP
18h
C
Golgi genes
mRNA (fold change)
UST
GALNT2
GOLGB1
COG5
GOLGA1
ARF4
COG1
GOLGA3
D
GO:0005925~focal adhesion
GO:0070062~extracellular exosome
GO:0005764~lysosome
GO:0005768~endosome
GO:0030141~secretory granule
GO:0031984~organelle subcompartment
GO:0031252~cell leading edge
GO:0030863~cortical cytoskeleton
GO:0030133~transport vesicle
GO:0005885~Arp2/3 protein complex
Enrichment score
E
0h
6h
CREB3L1
DAB
Nuclei
TFEB
F
CREB3L1
TFEB
Nucleus/cytoplasm ratio
p=6.16E-05
Time (h)

 © The Author(s)

Figure EV4. Endosome inactivation does not induce Golgi gene expression.

(**A**) HeLa cells were incubated with HRP for 30 min. After HRP loading, endosomes were inactivated using DAB and H_2O_2; DAB colocalized with LAMP1 and EEA1 but not with GM130, indicating the Golgi apparatus was unaffected. (**B**) HRP uptake was minimal immediately after endosome inactivation (0 h) but was restored by 18 h, demonstrating functional and structural recovery of endosomes. (**C**) qRT-PCR analysis showed no significant changes in Golgi gene expression at 18 h post-inactivation compared to 0 h, despite endosome regeneration (mean ± SD; *t* test; $n = 3$ replicates). (**D**) Transcriptional profiles of cells subjected to endosome inactivation (treated with DAB and H_2O_2) and control cells (treated with H_2O_2 only) were compared by RNA-seq. The graph displays the top 10 GO enriched terms for genes upregulated in endosome-inactivated cells, with "Lysosome" and "Endosome" terms (highlighted in red) present but not Golgi-related terms. (**E**) Images show no nuclear translocation of CREB3L1 after endosome inactivation, whereas clear nuclear translocation of the lysosome-specific transcription factor TFEB was observed. (**F**) Quantification of the nuclear/cytoplasmic ratio shows no increase for CREB3L1 and a strong increase for TFEB after endosome inactivation (mean ± SD, two-way ANOVA; $n = 3$ replicates). Scale bars: 10 µm (**A**, **B**, **E**).

© The Author(s)
