# Supplementary material for: A coordinated transcriptional program controls de novo Golgi biogenesis
Source: EMBO J. 2026 Jun 6;45(16):5851–83. doi: 10.1038/s44318-026-00828-7 (PMC13483034; doi:10.1038/s44318-026-00828-7)
Supplement: Supplementary file 3 [file 44318_2026_828_MOESM2_ESM.pdf]

THE EMBO JOURNAL

# A coordinated transcriptional program controls de novo Golgi biogenesis

Francesca Forno, Domenico Abete, Elena Polishchuk, Xabier Bujanda Cundin, Fioranna Renda, Roberta Crispino, Josephine Salzano, Raffaella Petruzzelli, Rossella De Cegli, Martina Sofia, Nicolina Sorrentino, Lorenzo Vaccaro, Davide Cacchiarelli, Juul Verbakel, Jan De Boer, Bruno Goud, Alexey Khodjakov, Franck Perez, and Roman Polishchuk

*Corresponding author(s): Roman Polishchuk (polish@tigem.it) , Franck Perez (franck.perez@curie.fr)*

## Review Timeline:

| | |
|---|---|
| Submission Date: | 17th Nov 25 |
| Editorial Decision: | 19th Dec 25 |
| Revision Received: | 14th Apr 26 |
| Editorial Decision: | 8th May 26 |
| Revision Received: | 14th May 26 |
| Accepted: | 17th May 26 |

*Editor: William Teale*

## Transaction Report:

(Note: With the exception of the correction of typographical or spelling errors that could be a source of ambiguity, letters and reports are not edited. Depending on transfer agreements, referee reports obtained elsewhere may or may not be included in this compilation. Referee reports are anonymous unless the Referee chooses to sign their reports.)

Dear Roman, dear Franck,

Thank you again for the submission of your manuscript entitled "De novo Golgi biogenesis requires coordinated transactivation of a Golgi regulon". We have now received reports from three referees, which I copy below.

As you can see from their comments, all thought that your manuscript could represent an important contribution to our understanding of Golgi biogenesis. That said, all of them make suggestions that you might consider addressing with a small extra investment of lab time.

Based on the overall interest expressed in the reports, I would like to invite you to address the comments of all referees in a revised version of the manuscript. I should add that it is The EMBO Journal policy to allow only a single major round of revision and that it is therefore important to resolve the main concerns at this stage. I believe the concerns of the referees are reasonable and addressable, but please contact me if you have any questions, need further input on the referee comments or if you anticipate any problems in addressing any of their points. Please, follow the instructions below when preparing your manuscript for resubmission.

I would also like to point out that as a matter of policy, competing manuscripts published during this period will not be taken into consideration in our assessment of the novelty presented by your study ("scooping" protection). We have extended this 'scooping protection policy' beyond the usual 3 month revision timeline to cover the period required for a full revision to address the essential experimental issues. Please contact me if you see a paper with related content published elsewhere to discuss the appropriate course of action.

When preparing your letter of response to the referees' comments, please bear in mind that this will form part of the Review Process File, and will therefore be available online to the community. For more details on our Transparent Editorial Process, please visit our website: https://link.springer.com/journal/44318/submission-guidelines#cms-Revised-submissions

Again, please contact me at any time during revision if you need any help or have further questions.

Thank you very much again for the opportunity to consider your work for publication. I look forward to your revision.

Best regards,

William

------------------------------
William Teale, Ph.D.
Editor
The EMBO Journal

When submitting your revised manuscript, please carefully review the instructions below and include the following items:

1) a .docx formatted version of the manuscript text (including legends for main figures, EV figures and tables). Please make sure that the changes are highlighted to be clearly visible.

2) individual production quality figure files as .eps, .tif, .jpg (one file per figure).

3) a .docx formatted letter INCLUDING the reviewers' reports and your detailed point-by-point response to their comments. As part of the EMBO Press transparent editorial process, the point-by-point response is part of the Review Process File (RPF), which will be published alongside your paper.

4) a complete author checklist, which you can download from our author guidelines (https://link.springer.com/journal/44318/submission-guidelines#cms-Revised-submissions). Please insert information in the checklist that is also reflected in the manuscript. The completed author checklist will also be part of the RPF.

5) Please note that all corresponding authors are required to supply an ORCID ID for their name upon submission of a revised manuscript.

6) We require a 'Data Availability' section after the Materials and Methods. Before submitting your revision, primary datasets produced in this study need to be deposited in an appropriate public database, and the accession numbers and database listed under 'Data Availability'. Please remember to provide a reviewer password if the datasets are not yet public (see https://link.springer.com/partners/embo-press/editorial-policies#Data%20deposition). If no data deposition in external databases

is needed for this paper, please then state in this section: This study includes no data deposited in external repositories. Note that the Data Availability Section is restricted to new primary data that are part of this study.

Note - All links should resolve to a page where the data can be accessed.

7) When assembling figures, please refer to our figure preparation guideline in order to ensure proper formatting and readability in print as well as on screen:
https://link.springer.com/journal/44318/submission-guidelines#cms-Figure-and-data-presentation

Please remember: Digital image enhancement is acceptable practice, as long as it accurately represents the original data and conforms to community standards. If a figure has been subjected to significant electronic manipulation, this must be noted in the figure legend or in the 'Materials and Methods' section. The editors reserve the right to request original versions of figures and the original images that were used to assemble the figure.

8) For data quantification: please specify the name of the statistical test used to generate error bars and P values, the number (n) of independent experiments (specify technical or biological replicates) underlying each data point and the test used to calculate p-values in each figure legend. The figure legends should contain a basic description of n, P and the test applied. Graphs must include a description of the bars and the error bars (s.d., s.e.m.).

9) We would also encourage you to include the source data for figure panels that show essential data. Numerical data can be provided as individual .xls or .csv files (including a tab describing the data). For 'blots' or microscopy, uncropped images should be submitted (using a zip archive or a single pdf per main figure if multiple images need to be supplied for one panel). Additional information on source data and instruction on how to label the files are available at
https://link.springer.com/journal/44318/submission-guidelines#cms-Source-data

10) We replaced Supplementary Information with Expanded View (EV) Figures and Tables that are collapsible/expandable online (see examples in https://www.embopress.org/doi/10.15252/embj.201695874). A maximum of 5 EV Figures can be typeset. EV Figures should be cited as 'Figure EV1, Figure EV2" etc. in the text and their respective legends should be included in the main text after the legends of regular figures.

- For the figures that you do NOT wish to display as Expanded View figures, they should be bundled together with their legends in a single PDF file called *Appendix*, which should start with a short Table of Content. Appendix figures should be referred to in the main text as: "Appendix Figure S1, Appendix Figure S2" etc. See detailed instructions regarding expanded view here: < https://link.springer.com/journal/44318/submission-guidelines#cms-Expanded-View-data >.

- Additional Tables/Datasets should be labelled and referred to as Table EV1, Dataset EV1, etc. Legends have to be provided in a separate tab in case of .xls files. Alternatively, the legend can be supplied as a separate text file (README) and zipped together with the Table/Dataset file.

11) At EMBO Press we ask authors to provide source data for the main manuscript figures. Our source data coordinator will contact you to discuss which figure panels we would need source data for and will also provide you with helpful tips on how to upload and organize the files.

12) Our journal encourages inclusion of *data citations in the reference list* to directly cite datasets that were re-used and obtained from public databases. Data citations in the article text are distinct from normal bibliographical citations and should directly link to the database records from which the data can be accessed. In the main text, data citations are formatted as follows: "Data ref: Smith et al, 2001" or "Data ref: NCBI Sequence Read Archive PRJNA342805, 2017". In the Reference list, data citations must be labeled with "[DATASET]". A data reference must provide the database name, accession number/identifiers and a resolvable link to the landing page from which the data can be accessed at the end of the reference. Further instructions are available at < https://link.springer.com/journal/44318/submission-guidelines#cms-Reference-guidelines >.

13) In order to increase the reproducibility and reach of your work, The EMBO Journal includes a table of reagents that were used in the study. Please provide this along with your revisions.

--------------------------------------------------

Further instructions for preparing your revised manuscript:

Read our guidance for manuscript revisions and related editorial policies: https://link.springer.com/journal/44318/submission-guidelines#cms-Revised-submissions

Please make sure you upload a letter of response to the referees' comments together with the revised manuscript.

Please also check that the title and abstract of the manuscript are brief, yet explicit, even to non-specialists.

When assembling figures, please refer to our figure preparation guideline in order to ensure proper formatting and readability in print as well as on screen:
https://media.springernature.com/original/springer-cms/rest/v1/content/27825798/data/v1

At EMBO Press we ask authors to provide source data for the main manuscript figures. You will receive a separate email with instructions for providing source data with your revised manuscript, including how to upload and organize the files.

IMPORTANT: When you send the revision we will require
- a point-by-point response to the referees' comments, with a detailed description of the changes made (as a word file).
- a word file of the manuscript text.
- individual production quality figure files (one file per figure)
- a complete author checklist
- Expanded View files (replacing Supplementary Information)
- a Reagents and Tools Table as part of the Methods section

Please remember: Digital image enhancement is acceptable practice, as long as it accurately represents the original data and conforms to community standards. If a figure has been subjected to significant electronic manipulation, this must be noted in the figure legend or in the 'Materials and Methods' section. The editors reserve the right to request original versions of figures and the original images that were used to assemble the figure.

We realize that it is difficult to revise to a specific deadline. In the interest of protecting the conceptual advance provided by the work, we recommend a revision within 3 months (19th Mar 2026). Please discuss the revision progress ahead of this time with the editor if you require more time to complete the revisions. Use the link below to submit your revision:

https://emboj.msubmit.net/cgi-bin/main.plex

------------------------------------------------

Referee #1:

This paper explores the transcriptional coordination of Golgi biogenesis. By optimizing a method to selectively ablate the Golgi using enzyme-mediated inactivation, the authors were able to use single-cell RNA sequencing to identify a regulon that controls expression of a variety of Golgi proteins. They went on to identify CREB3L1 as a key transcriptional regulator in this pathway. The interpretation is that cells coordinately regulate expression of Golgi proteins when the organelle is damaged, and that this process likely reflects a physiological mechanism by which cells expand the Golgi in response to specific needs.

This work is novel and interesting, the narrative is presented well, and the analysis seems to be thorough. I have no specific issues with the methods used, although the transcriptomic and bioinformatic techniques are outside my expertise. However, I do have the following significant concern, which could be addressed experimentally if needed or else by a suitable explanation if appropriate.

The transcriptomic analysis yielded 12 clusters, each of which had upregulation of genes from multiple categories. For each of the two clusters that was scored positive, "Golgi apparatus" was not the most abundant category-indeed, it was the least abundant category for cluster 10. I have the impression that with such a rich and diverse data set, the authors were bound to find what they were looking for. Would any other type of cellular or organellar damage also have produced a similarly diverse response? As a control, after endosome inactivation (Figure S8), a few specific Golgi genes were not found to be transcriptionally activated, but what general categories of transcriptional upregulation would be seen with cluster analysis of those samples? Would "Golgi apparatus" have emerged in some of the clusters from the endosome treatment as well?

A minor point: the authors claim to have improved and optimized the DAB-mediated Golgi inactivation method, but I didn't see an explanation of the specific changes that were made. Please clarify.

Referee #2:

The manuscript by Forno, Abete et al. presents a highly compelling and original study identifying a Golgi "regulon" that governs

transcriptional responses during de novo Golgi biogenesis. Using a previously established enzyme-mediated Golgi inactivation approach, the authors extend earlier work by exploring later recovery time points and demonstrate that fully functional Golgi stacks can reform approximately 10 hours after inactivation. Single-cell RNA sequencing reveals a substantial remodeling of the transcriptome following Golgi inactivation, including coordinated upregulation of numerous Golgi-associated genes. Through an elegant and broad palette of experimental tools (cell biology, electron microscopy, live imaging, scRNA-seq, RNAi mini-screening, FISH, bioinformatics, etc.), the authors identify CREB3L1 as a key transcriptional regulator orchestrating this Golgi-specific transcriptional program.
This is the type of study that introduces a genuinely novel conceptual advance and has the potential to become a foundational reference in the field. As such, it naturally opens many new questions that will stimulate future work. The comments below are therefore intended to be constructive, aimed at improving clarity, rigor, and accessibility for a broad readership, and in some cases suggesting additional discussion or improved analysis rather than extensive new experimental work.

Major points

1. Quantification and statistical support for qualitative statements: Throughout the manuscript, qualitative descriptors are frequently used to describe observed effects (e.g., "significant", "substantial number of cells", "considerable variability", "frequently lacked", etc). I strongly encourage the authors to either provide quantitative measurements and proper statistical testing when possible, or explicitly state when observations are intended to be qualitative. Some specific examples (non-exhaustive):
- Page 3: "The proportion of such irregular stacks significantly increased...": Please indicate whether this increase was statistically tested. More generally, it would be helpful to clarify how the Golgi morphology fraction plots (e.g. Fig. S1F) were generated: (i) Are these data derived from a single biological replicate or pooled across multiple independent experiments? (ii) If multiple replicates exist, please include error bars and statistical analysis across biological replicates (similar comments apply to Fig. 4G).
- Figure 2C: The number of biological replicates is not indicated in the legend. If more than one replicate was performed, error bars and statistical analysis should be included. If only a single experiment was performed, I am not sure that firm conclusions about timing differences between Golgi markers can be drawn.
- Page 3, second-to-last paragraph: "can vary considerably from one cell to another"
Can the authors provide numerical measures of this variability (e.g. range, CV, or fraction of cells in different categories)?
- Section "De Novo Golgi Biogenesis Requires Transcription":
Phrases such as "substantial number of cells" and "frequently lacked" would benefit from quantification or clarification that these are qualitative assessments.

2. Link between ER export and Golgi biogenesis: On page 3, the authors state that Golgi formation "might be linked to the export of material from the ER." Have the authors considered experimentally perturbing ER export (e.g. partial inhibition as full inactivation could not be possible for such long period) to test whether ER export is required for the observed Golgi recovery?

3. Clarification of the DAB-based Golgi inactivation strategy: I think that, for non-expert readers, it would be helpful if the authors briefly explain early in the Results section the rationale and principle of the DAB-based Golgi inactivation method, even if it has been described in earlier work.

4. Representation of single-cell data and biological replication: In Fig. 7B, C (and related supplementary figures), each dot appears to represent a single cell. Please clarify (i) How many biological replicates contribute to these plots? (ii) Were cells pooled across experiments? I suggest considering SuperPlots (e.g. Lord et al., JCB 2020, https://doi.org/10.1083/jcb.202001064) or an equivalent approach, where individual cells are shown but biological replicates are clearly distinguished and used for statistical testing.
That said, I would like to emphasize that the control shown in Fig. 7C is a very important one.

5. Validation of siRNA knockdowns: I could not find validation data for the efficiency of siRNA-mediated knockdowns. While not all targets may be needed, I suggest including validation at least for CREB3L1, and ideally also SP1 and MITF.

6. Figure 8B: It might be informative to include panels showing siCREB3L1 conditions at 0 h ({plus minus} DAB), to clarify baseline Golgi status and facilitate interpretation of recovery defects.

7. Physiological relevance in differentiated cell types: This may be very well beyond the scope of the current manuscript, but the final part of the Discussion is particularly interesting. Are the authors considering validating aspects of this Golgi transcriptional program in physiologically relevant models such as B cells or endometrial stromal cells?

Minor points

- Introduction, page 1: The opening sentence refers to "Biogenesis" of the Golgi. Given the later discussion on Golgi expansion (e.g. in B cells and endometrial cells), would "Expansion" be more accurate, or should both terms be used (at the end, the authors' assay is indeed to study Golgi biogenesis)?

- Figure S3C-F: Including higher-magnification insets of selected regions would help clarify the message.

- Consider using color-blind-friendly palettes (e.g. replacing red/green with magenta/green in microscopy images).

- "Newly-formed Golgi supports..." section: The description of the experimental timeline (first paragraph) is somewhat unclear. Is biotin always added at time 0? Please clarify in the text, figure legends, or Methods.

- Movies 3-6: These movies are very informative, but the time scale is confusing. Please specify in the captions that the time is hh:mm (I guess this is so, right?). Also, playback does not appear temporally uniform (possibly due to variable frame rates). If possible, please correct and/or clarify this.

- Figure 5A: Does this plot include all cells from all time points? If so, please state this explicitly. Also, due to the number of colors, it is difficult to visually associate clusters with specific time points. Maybe adding cluster numbers in the actual PCA plot or arrows might help.

- Figure S6D (page 7): This is an excellent control. To fully interpret it, please indicate how many cells were analyzed here compared to Fig. 6C, and whether these numbers are comparable.
Typos and minor corrections

- Page 2: Add "cells" after "... (ManII-HRP) expressing ..."

- Page 2, section title: "Cell" → "Cells"; remove the "a" from "a 'new', but ..."

- Figure S4B: "GalT-GGF" likely should be "GalT-GFP". If not, please clarify what GGF refers to. Also, what do the authors think the vacuolar-like structures appearing over time are?

- Page 7: 6E → 6D; 6F → 6E
- Page 9: "CREBL1" appears in different places. Should this be "CREB3L1"?

As said, I think this is overall an exceptionally strong and conceptually exciting manuscript. With improved quantification, clearer presentation of replication and statistics, and minor clarifications, it could be a major contribution to our understanding of Golgi biogenesis and its transcriptional regulation.

Referee #3:

The manuscript by Forno et al reports on the identification of a genetic program that governs the biogenesis of the Golgi apparatus. They used DAB-based inactivation of Golgi structures followed by several hours of follow-up to monitor the dynamics of Golgi re-formation. The identified CREB3L1 as a key transcriptional regulator that is responsible for the induction of the Golgi transcriptional program.
Overall, the work is of high quality, novel and investigates an important topic. The results address a major gap in our understanding of Golgi biology and are of wide interest of scientists working in the field of membrane trafficking and organelle biology. I am very supportive of the publication of this work, but there are a number of points that should be addressed before. I organized my comments in the order they occurred while reading the text and therefore they are a blend of major and minor points.

1- It would help the readers who are not very familiar with electron microscopy of the authors explained how the identified ERES. Given recent data from the Lippincott-Schwartz and Zanetti labs, I think it is important that the authors carefully explain what they believe a structure is an ERES (e.g., in Figure 1B).

2- The authors claim that DAB selectively disrupts the Golgi, but does not affect ERES. It would be great to see functional evidence supporting this claim. This could (for example) be achieved using the RUSH assay showing that directly after inactivation of the Golgi, cargo can still concentrate into ERES or ERGIC (i.e., for puncta after adding biotin).

3- The experiment in Figure 3A indicates that ERES are not functional. There is no concentration of cargo in ERES or the ERGIC. Both structures should be preserved according to the interpretation of the authors that DAB selectively inactivates the Golgi.

4- Figure 5B: I don't understand what it actually means that different clusters are forming. The authors write: "....indicating that they might be associated with specific cell states/fates after inactivation of the Golgi apparatus". What are these cell states/fates? What is the functional significance.

5- What induces CREB3L1 expression or activates its function in cells recovering from DAB-induced Golgi inactivation? How does CREB3L1 sense that it is needed to do something? Or is this protein simply constitutively active?

**Referee #1:**

This paper explores the transcriptional coordination of Golgi biogenesis. By optimizing a method to selectively ablate the Golgi using enzyme-mediated inactivation, the authors were able to use single-cell RNA sequencing to identify a regulon that controls expression of a variety of Golgi proteins. They went on to identify CREB3L1 as a key transcriptional regulator in this pathway. The interpretation is that cells coordinately regulate expression of Golgi proteins when the organelle is damaged, and that this process likely reflects a physiological mechanism by which cells expand the Golgi in response to specific needs. This work is novel and interesting, the narrative is presented well, and the analysis seems to be thorough.

We thank the reviewer for the positive evaluation of our study.

I have no specific issues with the methods used, although the transcriptomic and bioinformatic techniques are outside my expertise. However, I do have the following significant concern, which could be addressed experimentally if needed or else by a suitable explanation if appropriate.

The transcriptomic analysis yielded 12 clusters, each of which had upregulation of genes from multiple categories. For each of the two clusters that was scored positive, "Golgi apparatus" was not the most abundant category-indeed, it was the least abundant category for cluster 10.

We would like to clarify that, for the sake of space in the figure, we displayed only the top 10 enriched GO terms for each cluster (as indicated in the figure legend). Including all statistically significant GO terms would make the graphs overly crowded. As a result, this may give the false impression that the term “Golgi apparatus” is the less abundant category in the ranking (especially in cluster 10), whereas it is simply the last among the top 10 terms, with additional enriched terms ranking below it.

I have the impression that with such a rich and diverse data set, the authors were bound to find what they were looking for. Would any other type of cellular or organellar damage also have produced a similarly diverse response? As a control, after endosome inactivation (Figure S8), a few specific Golgi genes were not found to be transcriptionally activated, but what general categories of transcriptional upregulation would be seen with cluster analysis of those samples? Would "Golgi apparatus" have emerged in some of the clusters from the endosome treatment as well?

This is a reasonable concern. The reviewer asks whether single-cell (scRNA-seq) analysis of cells with inactivated endosomes might also produce clusters enriched in upregulated Golgi genes.

We believe that scRNA-seq analysis is not necessary in this case due to a fundamental difference between the two experimental systems. Golgi inactivation in our study relies on expression of ManII-HRP, which varies substantially across the cell population. As a result, the degree of Golgi inactivation is highly heterogeneous between cells. This heterogeneity necessitated the use of scRNA-seq in order to resolve transcriptional signatures associated with different levels of Golgi damage.

In contrast, endosome inactivation is achieved through endocytic uptake of HRP, a process that occurs uniformly across the cell population. Consequently, the degree of endosome inactivation is much more homogeneous between cells, and bulk RNA-seq analysis is sufficient to capture the transcriptional response.

Using bulk RNA-seq, we found no significant enrichment of Golgi-related GO terms among the upregulated transcripts after endosome inactivation. The only partially related categories were “transport vesicle” and “membrane coat,” which included only five Golgi-relevant genes (COPB2, COPA, COPB1, CLTA, SEC31A). This number is very small compared with the more than 100 Golgi genes that were upregulated after Golgi inactivation. These results are consistent with our qRT-PCR data, which also show no induction of Golgi genes following endosome inactivation.

Instead, cells with inactivated endosomes showed enrichment of “endosome” and “lysosome” GO terms among the upregulated transcripts (Fig. EV4D), indicating activation of a distinct transcriptional program in response to endosomal dysfunction.

This conclusion is further supported by the behavior of transcription factors controlling these programs. CREB3L1, which drives Golgi gene expression, did not undergo nuclear translocation upon endosome

inactivation (Fig. EV4E, F), consistent with the absence of Golgi gene activation. In contrast, TFEB, a transcription factor that regulates endosomal and lysosomal genes (Sardiello et al., 2009), showed clear nuclear translocation under these conditions (Fig. EV4E, F), correlating with the observed transcriptional upregulation of endosomal and lysosomal genes.

Taken together, these results indicate that selective dysfunction of different organelles activates distinct, organelle-specific transcriptional programs.

A minor point: the authors claim to have improved and optimized the DAB-mediated Golgi inactivation method, but I didn't see an explanation of the specific changes that were made. Please clarify.

We agree that this may be an important point for researchers who would like to use our method to stimulate de novo Golgi biogenesis. In the revised manuscript, we clarify that three main modifications were introduced compared with the previously published protocol. First, incubation with DAB was performed at room temperature rather than on ice in order to minimize cellular stress during the procedure. Second, conditioned medium was used after inactivation to better support cell recovery following Golgi loss. Finally, the duration of incubation with DAB was determined based on the intensity of the DAB signal in the Golgi region rather than using a fixed incubation time. This approach helps avoid excessive DAB accumulation in the Golgi, which can lead to membrane rupture and consequently increased cell damage. All of these key modifications to the protocol are now explicitly described and emphasized in the Methods section.

**Referee #2:**

The manuscript by Forno, Abete et al. presents a highly compelling and original study identifying a Golgi "regulon" that governs transcriptional responses during de novo Golgi biogenesis. Using a previously established enzyme-mediated Golgi inactivation approach, the authors extend earlier work by exploring later recovery time points and demonstrate that fully functional Golgi stacks can reform approximately 10 hours after inactivation. Single-cell RNA sequencing reveals a substantial remodeling of the transcriptome following Golgi inactivation, including coordinated upregulation of numerous Golgi-associated genes. Through an elegant and broad palette of experimental tools (cell biology, electron microscopy, live imaging, scRNA-seq, RNAi mini-screening, FISH, bioinformatics, etc.), the authors identify CREB3L1 as a key transcriptional regulator orchestrating this Golgi-specific transcriptional program.
This is the type of study that introduces a genuinely novel conceptual advance and has the potential to become a foundational reference in the field. As such, it naturally opens many new questions that will stimulate future work. The comments below are therefore intended to be constructive, aimed at improving clarity, rigor, and accessibility for a broad readership, and in some cases suggesting additional discussion or improved analysis rather than extensive new experimental work.

We thank the reviewer for very positive feedback.

Major points

1. Quantification and statistical support for qualitative statements: Throughout the manuscript, qualitative descriptors are frequently used to describe observed effects (e.g., "significant", "substantial number of cells", "considerable variability", "frequently lacked", etc). I strongly encourage the authors to either provide quantitative measurements and proper statistical testing when possible, or explicitly state when observations are intended to be qualitative.

According to the reviewer's suggestion we provided quantification and statistical support where it was applicable. This includes addition of new quantifications in Figures 2D, 4E, 9D; Extended View Figures 2B,G, 4F and Appendix Supplementary Figure 4A. Furthermore, in line with reviewer's suggestions we also modified Figures 2C, 3G, 4H, 7B,C, 8E, 9B and Appendix Supplementary Figure 3F.

Some specific examples (non-exhaustive):
- Page 3: "The proportion of such irregular stacks significantly increased...": Please indicate whether this increase was statistically tested. More generally, it would be helpful to clarify how the Golgi morphology fraction plots (e.g. Fig. S1F) were generated: (i) Are these data derived from a single biological replicate or pooled across multiple independent experiments? (ii) If multiple replicates exist, please include error bars and statistical analysis across biological replicates (similar comments apply to Fig. 4G).

We replaced the pie charts (former Fig. S1F) with grouped bar plots showing the fraction of cells in each Golgi morphology category, including biological replicates and statistical testing for differences between time points (Fig. EV1B). We implemented the same changes for the former Fig. 4G, which is now presented as Fig. 4H in the revised manuscript.

- Figure 2C: The number of biological replicates is not indicated in the legend. If more than one replicate was performed, error bars and statistical analysis should be included. If only a single experiment was performed, I am not sure that firm conclusions about timing differences between Golgi markers can be drawn.

Figure 2C was originally presented without SD bars or statistical testing to avoid an overly busy plot. To address the reviewer's concern, we revised Figure 2C by adding SD bars and the results of ANOVA, providing statistical support for differences in recovery dynamics among Golgi markers during organelle biogenesis. The number of biological replicates is now indicated in the figure legend. Furthermore, we added a hierarchical clustering analysis that summarizes the similarity among markers based on their recovery kinetics in the newly forming Golgi (new Fig. 2D).

- Page 3, second-to-last paragraph: "can vary considerably from one cell to another"
Can the authors provide numerical measures of this variability (e.g. range, CV, or fraction of cells in different categories)?

The text was modified to address the reviewer's comment. Statistical support for cell-to-cell variability was included (Fig. EV1B) and is based on the data showing the fractions of cells exhibiting different Golgi phenotypes at the examined time points. The revised sentence now reads: "However, this process requires a significant amount of time (usually ≥10 hours) and can vary considerably from one cell to another, as evidenced by the substantial fractions of cells displaying different Golgi phenotypes even at the 10- and 24-hour time points (Fig. EV1B)."

- Section "De Novo Golgi Biogenesis Requires Transcription":
Phrases such as "substantial number of cells" and "frequently lacked" would benefit from quantification or clarification that these are qualitative assessments.

We addressed this point by adding quantification in Fig. 4E, which supports these statements and clarifies the frequency of the reported phenotypes.

2. Link between ER export and Golgi biogenesis: On page 3, the authors state that Golgi formation "might be linked to the export of material from the ER." Have the authors considered experimentally perturbing ER export (e.g. partial inhibition as full inactivation could not be possible for such long period) to test whether ER export is required for the observed Golgi recovery?

We thank the reviewer for this insightful suggestion. We have made every possible effort within the limits of our experimental system to address this point.

Several of our existing observations already support a functional link between ER export and the formation of new Golgi units. First, our electron microscopy and tomography data show that newly forming Golgi structures appear in close proximity to ER exit sites (arrowheads in Fig. 1B and Movie 1), indicating a clear spatial coupling between ER export and Golgi biogenesis.

Second, our Actinomycin D experiments indicate that the formation of a new Golgi requires de novo transcription and subsequent biosynthesis of Golgi-resident transmembrane proteins (e.g., GalT; Fig. 4D,

E). As these proteins are synthesized and inserted into the ER membrane before being transported forward, their requirement strongly implies that ER export is an essential step in the recovery process.

In addition, we have experimentally attempted to perturb ER export, to the extent that is technically feasible in this system. We would like to emphasize that direct and specific inhibition of ER export over the long time frame required for Golgi recovery is inherently challenging. Genetic approaches (e.g., expression of SAR1 mutants) lead to rapid accumulation of Golgi enzymes in the ER (Ward et al., 2001), which in our system would compromise the specificity of the ManII-HRP–based Golgi inactivation by effectively extending it to the ER, likely resulting in severe cellular stress or loss of viability. For this reason, such approaches are not compatible with our experimental setup.

As an alternative, we tested the effect of Brefeldin A (BFA), which remains the only widely used pharmacological inhibitor of ER export (Fujiwara et al. 1988; Lippincott-Schwartz et al. 1989). When applied immediately after Golgi inactivation, BFA strongly impaired the formation of new Golgi structures (see figure below), further supporting the requirement of ER export for Golgi reassembly. However, we chose not to include the BFA experiment in the manuscript because (i) the manuscript is already densely packed with experimental data, and (ii) BFA has broad effects on the secretory pathway beyond ER export, which could complicate the interpretation. Nevertheless, we are happy to include these data if the reviewer and editor feel that they would strengthen the manuscript.

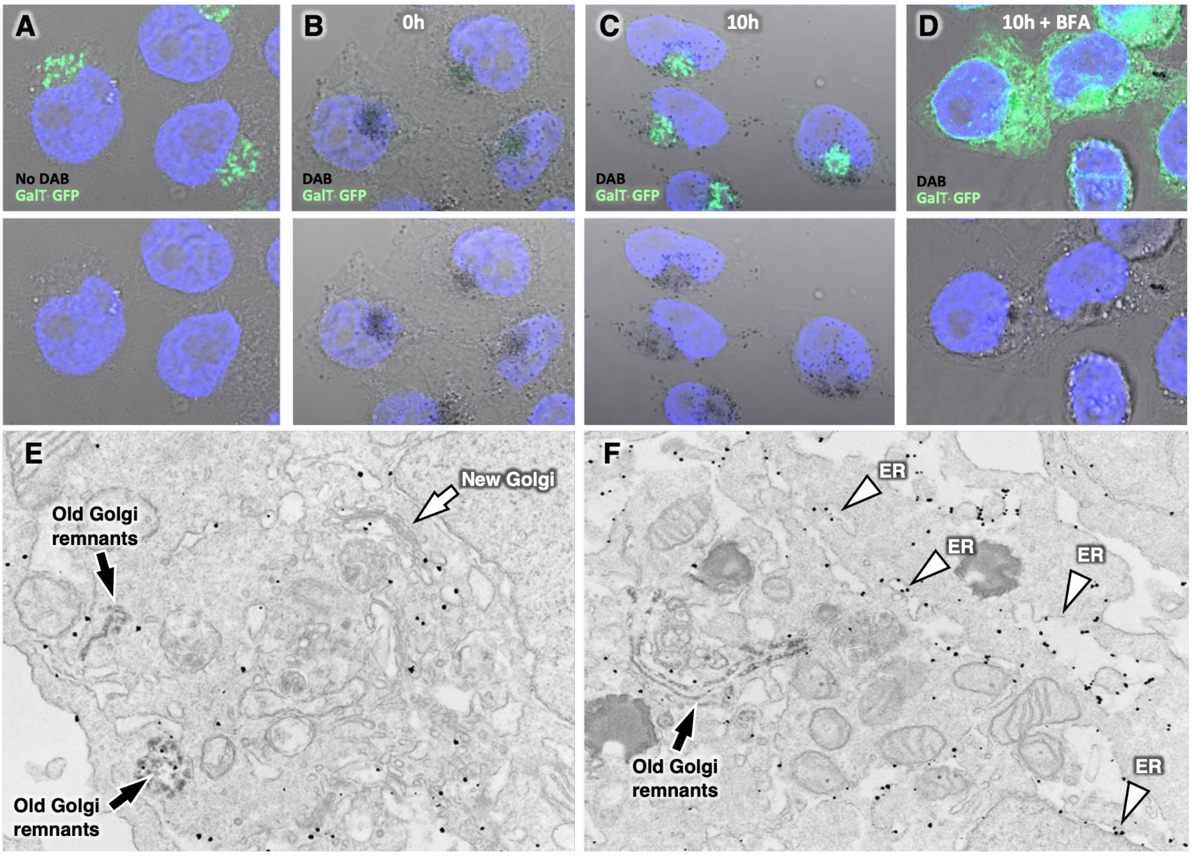


**BFA inhibits de novo Golgi biogenesis.**
HeLa cells expressing ManII-HRP and GalT-GFP were incubated with DAB and $H_2O_2$ to cross-link the preexisting (old) Golgi and then fixed for IF (A-D) or immuno-gold EM (E, F) analyses either immediately (B) or 10 hours after incubation with fresh medium in the absence (C, E) or presence (D, F) of BFA. The GalT-GFP signal visible in intact cells (A) is quenched by DAB deposition (B). Newly synthesised GalT-GFP was detected in the newly forming Golgi 10 h after inactivation of the old organelle in control cells, but remained arrested in the ER in BFA-treated cells. Immuno-gold EM revealed GalT-GFP in the new Golgi stacks (E, white arrow) 10h after Golgi inactivation. In contrast, only the old DAB-positive Golgi was detected in BFA-treated cells (F, black arrow). No newly forming Golgi stacks were observed, while GalT-GFP was retained in the ER (white arrowheads).

3. Clarification of the DAB-based Golgi inactivation strategy: I think that, for non-expert readers, it would

be helpful if the authors briefly explain early in the Results section the rationale and principle of the DAB-based Golgi inactivation method, even if it has been described in earlier work.

In the revised Results section, we now briefly explain the principle of DAB-mediated Golgi inactivation for clarity, as requested. This description emphasizes that the DAB-based approach permits the selective inactivation of the Golgi apparatus in numerous cells simultaneously, enabling robust population-level analysis. Other aspects, including the rationale for choosing DAB-mediated inactivation over alternative methods such as BFA or laser ablation, are already outlined in the Introduction. There, we highlight that, unlike BFA—which broadly disrupts the secretory pathway—DAB crosslinks Golgi components and makes them unavailable for de novo Golgi biogenesis, requiring cells to synthesize new Golgi-resident proteins. The method also offers advantages over laser ablation, which is limited to only a few cells at a time. Thus, the Results section focuses on the principle of the method, while the rationale is discussed in the Introduction.

4. Representation of single-cell data and biological replication: In Fig. 7B, C (and related supplementary figures), each dot appears to represent a single cell. Please clarify (i) How many biological replicates contribute to these plots? (ii) Were cells pooled across experiments? I suggest considering SuperPlots (e.g. Lord et al., JCB 2020,https://doi.org/10.1083/jcb.202001064) or an equivalent approach, where individual cells are shown but biological replicates are clearly distinguished and used for statistical testing. That said, I would like to emphasize that the control shown in Fig. 7C is a very important one.

In response to the reviewer's suggestion, we reorganized the data in Fig. 7B and 7C into a SuperPlot format (as described by Lord et al., JCB 2020), in which individual cells are displayed while biological replicates are clearly distinguished and used as the basis for statistical testing. The number of biological replicates contributing to each dataset is now indicated in the figure legends. Cells were not pooled for statistical analysis; instead, replicate-level values were used for statistical comparisons. We have applied the same SuperPlot format to other applicable experiments (Figures 3G, 8E, 9B, EV2G). Figure 8B, which presents the RNAi-based mini-screening experiment, was performed in a single biological replicate and therefore could not be represented in SuperPlot format. This is now clearly indicated in the legend.

5. Validation of siRNA knockdowns: I could not find validation data for the efficiency of siRNA-mediated knockdowns. While not all targets may be needed, I suggest including validation at least for CREB3L1, and ideally also SP1 and MITF.

We now provide qRT-PCR data demonstrating the degree of silencing for CREB3L1, MITF, and SP1 (Appendix Figure S4A). Additionally, the efficiency of silencing for other transcription factors included in the RNAi mini-screening was also confirmed by qRT-PCR. Following the reviewer's suggestion, validation for targets beyond CREB3L1, MITF, and SP1 are not shown in the figures but mentioned in the Methods section.

6. Figure 8B: It might be informative to include panels showing siCREB3L1 conditions at 0 h ({plus minus} DAB), to clarify baseline Golgi status and facilitate interpretation of recovery defects.

Two additional panels showing CREB3L1-silenced cells at 0 h (±DAB) were included in Figure 8B.

7. Physiological relevance in differentiated cell types: This may be very well beyond the scope of the current manuscript, but the final part of the Discussion is particularly interesting. Are the authors considering validating aspects of this Golgi transcriptional program in physiologically relevant models such as B cells or endometrial stromal cells?

We agree with the reviewer that validation of our findings in physiologically relevant contexts is important to establish their physiological relevance. We believe, however, that already published data provide at least partial validation of the Golgi-associated transcriptional program described in our study.

Specifically, silencing of CREB3L1, either alone or in combination with CREB3L2, was shown to inhibit the expression of Golgi-related genes in differentiating endometrial stromal cells, thereby preventing Golgi

expansion and activation of secretory activity (Pittari et al., 2022). In addition, less direct but nonetheless significant evidence supporting a CREB3L1-mediated mechanism comes from studies in B cells, where differentiation-associated reprogramming toward secretory activity was suppressed by inhibition of site-1 protease, which is required for CREB3L1 cleavage and nuclear translocation (Al-Maskari et al., 2018).

We have therefore expanded the Discussion to emphasize that these studies provide experimental evidence for the physiological relevance of the Golgi-associated transcriptional mechanism described in our work. In future studies, we plan to directly test specific aspects of this mechanism—such as CREB3L1 nuclear translocation—in cells that activate secretory programs during differentiation or in response to physiological stimuli. However, as the reviewer notes, such experiments would require substantial additional effort and are beyond the scope of the current manuscript.

Minor points

- Introduction, page 1: The opening sentence refers to "Biogenesis" of the Golgi. Given the later discussion on Golgi expansion (e.g. in B cells and endometrial cells), would "Expansion" be more accurate, or should both terms be used (at the end, the authors' assay is indeed to study Golgi biogenesis)?

We agree that “Golgi expansion” is an important concept, particularly in differentiated secretory cells. However, we believe that “biogenesis” is the accurate term in the specific context of our manuscript. In our view, “expansion” is a broader process that can, in principle, occur by increased flux and remodeling within the secretory pathway without necessarily requiring de novo synthesis of Golgi building blocks. By contrast, “biogenesis” emphasizes the active addition of Golgi elements through de novo transcription/translation and membrane trafficking. Importantly, Golgi expansion can be achieved through Golgi biogenesis, but is not synonymous with it. As the reviewer correctly notes, our experimental system and the central focus of this study are on Golgi biogenesis.

- Figure S3C-F: Including higher-magnification insets of selected regions would help clarify the message.

We have included higher-magnification insets for the LC3 panels (Figure S3C,D in the original manuscript; Figure EV1E,F in the revised version). Due to space constraints, we removed the LAMP1 panels (Figure S3E,F in the original manuscript), as DAB-positive cisternae derived from the old Golgi can be clearly observed within lysosomes in the EM images (e.g., Figure EV1D in the revised version).

- Consider using color-blind-friendly palettes (e.g. replacing red/green with magenta/green in microscopy images).

We initially considered using magenta instead of red to make the images more color-blind-friendly. However, this approach created a significant issue in our case. When green and magenta channels overlap, they produce a white signal in regions of colocalization. When these channels were further overlaid with the bright-field channel (which is required to visualize the DAB signal), the resulting images became difficult to interpret because the white colocalization signal was poorly distinguishable from the bright-field background in many cases.

In contrast, colocalization between red and green produces a yellow signal that remains clearly visible even when the fluorescence channels are combined with the bright-field image. For this reason, we retained the red channel, as switching to magenta would not provide a substantial advantage for color-blind readers but would significantly compromise the interpretability of the images.

Where possible (such as in Figure EV1E), red was replaced with green in microscopy images to improve accessibility for color-blind readers.

- "Newly formed Golgi supports..." section: The description of the experimental timeline (first paragraph) is somewhat unclear. Is biotin always added at time 0? Please clarify in the text, figure legends, or Methods.

We confirm that biotin was added immediately after Golgi inactivation, at time 0. To enhance clarity, we have explicitly stated this in the result section of revised manuscript, as recommended by the reviewer.

- Movies 3-6: These movies are very informative, but the time scale is confusing. Please specify in the captions that the time is hh:mm (I guess this is so, right?). Also, playback does not appear temporally uniform (possibly due to variable frame rates). If possible, please correct and/or clarify this.
As suggested by the reviewer, we have updated the movie captions to specify the time format as hh:mm. Regarding the playback rate, the movies intentionally display variable temporal resolution for a specific reason. During the initial stage following biotin addition, images were acquired at shorter intervals to capture the rapid ER-to-Golgi transport. Once the cargo reached the Golgi (approximately 30–40 minutes after biotin addition), the imaging intervals were extended substantially in order to minimize phototoxicity during prolonged observation. This information is now clearly reported in the movie legend. We found that higher frame rates caused pronounced cell stress, as the cells were already under strain from transfection, cargo accumulation in the ER, and Golgi inactivation. Therefore, our time-lapse strategy was carefully chosen to balance the need for temporal detail with the necessity to preserve cell viability.

- Figure 5A: Does this plot include all cells from all time points? If so, please state this explicitly. Also, due to the number of colors, it is difficult to visually associate clusters with specific time points. Maybe adding cluster numbers in the actual PCA plot or arrows might help.

We have revised the figure to address reviewer's comments. Specifically, we now clearly state that the UMAP in Figure 5A includes cells from all time points. In addition, we have added cluster numbers directly onto the UMAP plot, which should make it easier to associate each color with its respective cluster. We believe these changes will facilitate the visual association of clusters with specific time points, as shown in the individual UMAPs in Figure 5B, and improve the overall clarity of the figure.

- Figure S6D (page 7): This is an excellent control. To fully interpret it, please indicate how many cells were analyzed here compared to Fig. 6C, and whether these numbers are comparable.
In this control, we analyzed individual transcriptomes from approximately 83,000 cells lacking detectable ManII-HRP expression, as now indicated in the figure legend (Fig. EV3D in the revised manuscript). This sample size is directly comparable to the roughly 35,000 cells with detectable ManII-HRP transcripts used to generate the data shown in Fig. 6C. Therefore, the numbers of cells analyzed in both conditions are similar, allowing for a meaningful comparison between the control and experimental groups.

Typos and minor corrections

- Page 2: Add "cells" after "... (ManII-HRP) expressing ..."

- Page 2, section title: "Cell" → "Cells"; remove the "a" from "a 'new', but ..."

- Figure S4B: "GalT-GGF" likely should be "GalT-GFP". If not, please clarify what GGF refers to. Also, what do the authors think the vacuolar-like structures appearing over time are?

- Page 7: 6E → 6D; 6F → 6E
- Page 9: "CREBL1" appears in different places. Should this be "CREB3L1"?

All corrections were made.

As said, I think this is overall an exceptionally strong and conceptually exciting manuscript. With improved quantification, clearer presentation of replication and statistics, and minor clarifications, it could be a major contribution to our understanding of Golgi biogenesis and its transcriptional regulation.

We sincerely appreciate the reviewer's feedback and fully share his/her perspective that this study represents a significant advance in understanding the role of transcription in Golgi biogenesis and the expansion of its functional capabilities.

Referee #3:

The manuscript by Forno et al reports on the identification of a genetic program that governs the biogenesis of the Golgi apparatus. They used DAB-based inactivation of Golgi structures followed by several hours of follow-up to monitor the dynamics of Golgi re-formation. The identified CREB3L1 as a key transcriptional regulator that is responsible for the induction of the Golgi transcriptional program. Overall, the work is of high quality, novel and investigates an important topic. The results address a major gap in our understanding of Golgi biology and are of wide interest of scientists working in the field of membrane trafficking and organelle biology. I am very supportive of the publication of this work, but there are a number of points that should be addressed before. I organized my comments in the order they occurred while reading the text and therefore they are a blend of major and minor points.

We thank the reviewer for the overall positive assessment of our study.

1- It would help the readers who are not very familiar with electron microscopy of the authors explained how they identified ERES. Given recent data from the Lippincott-Schwartz and Zanetti labs, I think it is important that the authors carefully explain what they believe a structure is an ERES (e.g., in Figure 1B).
In response to the reviewer's suggestion, we revised the text (page 3) to emphasize the ultrastructural features we used to identify ER exit sites (ERES), including exclusion of ribosomes from these ER surface domains, the presence of multiple COPII-coated buds, and adjacent clusters of tubular–vesicular membranes (Bannykh and Balch, 1996; Mironov et al., 2003; Weigel et al., 2021). We also added EM tomography (Movie 1) to further support our conclusion that ERES are closely associated with newly forming Golgi structures.

2- The authors claim that DAB selectively disrupts the Golgi, but does not affect ERES. It would be great to see functional evidence supporting this claim. This could (for example) be achieved using the RUSH assay showing that directly after inactivation of the Golgi, cargo can still concentrate into ERES or ERGIC (i.e., for puncta after adding biotin).
As suggested by the reviewer, we performed live cell imaging to test whether the GPI-RUSH cargo can still concentrate at ERES after Golgi inactivation. We observed efficient accumulation of GPI-RUSH at ERES with kinetics comparable to control cells (see Movies 4 and 5). In addition, newly forming GPI-RUSH carriers were positive for the ERES marker Sec24 in both DAB-treated and control cells (Extended View Figure 2A,B), supporting the conclusion that ERES remain functional after Golgi inactivation.

3- The experiment in Figure 3A indicates that ERES are not functional. There is no concentration of cargo in ERES or the ERGIC. Both structures should be preserved according to the interpretation of the authors that DAB selectively inactivates the Golgi.
We cannot fully agree with this interpretation. Figure 3A does not include time points at which GPI-RUSH accumulation at ERES/ERGIC can be appreciated immediately after Golgi inactivation. The figure starts at 0 h, when GPI-RUSH is retained in the ER, and the next time point shown is 2 h, when GPI-RUSH has already exited the ER and is either trapped in newly forming Golgi structures in cells with crosslinked old Golgi or, in cells where DAB-mediated crosslinking did not occur, has largely reached the cell surface. Thus, Figure 3A does not report the trafficking steps occurring between 0 and 2 h post-inactivation. To address this gap, in the revised manuscript we now provide (as outlined above) live-cell imaging and Sec24 colocalization data demonstrating that cargo export through ERES proceeds efficiently after Golgi inactivation, indicating that ERES remain functional under these conditions.

4- Figure 5B: I don't understand what it actually means that different clusters are forming. The authors write: "....indicating that they might be associated with specific cell states/fates after inactivation of the Golgi apparatus". What are these cell states/fates? What is the functional significance.
We would like to clarify that the persisting transcriptional clusters (which may represent specific cell states or fates) emerge only starting at 10 hours after Golgi inactivation. These clusters are consistently

present at the 10, 14, 18, and 24 hour time points. In contrast, before 10 hours (i.e., at 0, 2, and 6 hours post-inactivation), each time point displays its own distinct clusters, reflecting a time-dependent transcriptional response to Golgi inactivation (Fig. 6B). After 10 hours, the appearance of persistent clusters suggests that the transcriptomes of individual cells are no longer strictly defined by time after inactivation, but rather by the **specific cellular response or fate** following Golgi disruption.

The notable heterogeneity in responses across cellular population cab be exemplified by below image showing 10h time point after Golgi inactivation: (1) some cells fully recover the Golgi, as indicated by the presence of both GM130 and GALNT2; (2) others recover only GM130 and thus regenerate the Golgi more slowly; (3) some cells do not recover the Golgi but remain viable; (4) there are dying cells, likely damaged by excessive DAB deposition due to higher ManII-HRP expression; (5) and finally, some cells show no evidence of DAB inactivation, presumably because of low ManII-HRP expression, and thus their transcriptional response is not related to Golgi loss.

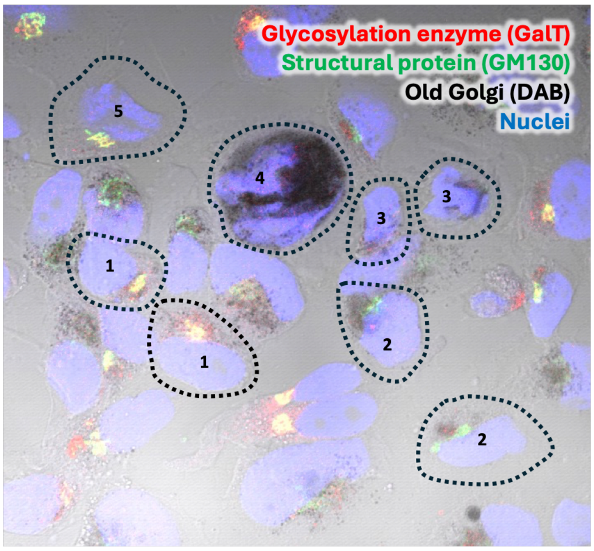


1) Complete recovery

2) Partial recovery

3) No recovery

4) Cell death

5) No ManII-HRP expression (no DAB), no inactivation of the Golgi

**Heterogeneity of cell phenotypes 10 hours after Golgi inactivation**
HeLa cells expressing ManII-HRP were incubated with DAB and H2O2 to cross-link the preexisting (old) Golgi, then fixed 10 hours after incubation with fresh medium and stained for GM130 and GalT. Each number indicates a cell with a specific phenotype, as listed near the figure.

We believe that this heterogeneity underlies the formation of the different cell clusters in transcriptomics analysis. For example, clusters 2 and 10 are characterized by upregulation of Golgi genes, which, as confirmed by subsequent FISH experiments, occurs in cells actively rebuilding the Golgi apparatus. Therefore, these clusters likely represent cells engaged in Golgi biogenesis. In contrast, cluster 4 is enriched in apoptotic genes, suggesting it corresponds to dying cells observed after Golgi inactivation. While our study focused on clusters with upregulated Golgi genes, a comprehensive characterization and validation of all clusters would require significant additional effort and was beyond the scope of this work.

5- What induces CREB3L1 expression or activates its function in cells recovering from DAB-induced Golgi inactivation? How does CREB3L1 sense that it is needed to do something? Or is this protein simply constitutively active?

We agree with reviewer that understanding how CREB3L1 is induced and activated during Golgi recovery represents an important mechanistic issue. However, we would like to note that dissecting the precise sensing and signaling pathways that couple Golgi reassembly to CREB3L1 activation would require a substantial, dedicated effort well beyond the scope of the present study. Based on our experience and by analogy to other systems (e.g., the elucidation of TFEB regulatory pathways for lysosome biogenesis), defining the upstream machinery controlling CREB3L1 activation would likely constitute a separate research program requiring several years of work.

Within the scope of the current manuscript, we have instead focused on establishing (i) that CREB3L1 is functionally involved in Golgi biogenesis and (ii) that it is dynamically activated during this process. Importantly, our data directly argue against the idea that CREB3L1 is constitutively active: we observe a clear and transient increase in its nuclear translocation during Golgi reformation (Fig. 9A,B), which is a well-established indication of CREB3L1 activation (Kondo et al., 2005).

Regarding the upstream trigger, we agree that this remains an open question. In the revised manuscript, we have expanded the Discussion to plausible mechanisms, while explicitly stating that these remain hypotheses. Briefly, our data support a model in which CREB3L1 activation is linked to transient trafficking imbalances during early Golgi reassembly. At early stages after DAB washout (2–6 h), newly forming Golgi membranes are capable of receiving cargo but are inefficient in exporting it, resulting in cargo accumulation and swelling of Golgi compartments. Such a "traffic jam" at the ER–Golgi interface could generate signals that promote CREB3L1 activation.

Mechanistically, we outline two non-mutually exclusive scenarios. First, cargo overload at the nascent Golgi may activate signaling pathways that promote CREB3L1 trafficking from the ER to the Golgi, where it undergoes S1P-mediated cleavage, analogous to other ER-resident transcription factors such as ATF6 and SREBPs. Second, our data indicate that retrograde trafficking from the newly forming Golgi to the ER is impaired, leading to retention of ER chaperones (e.g., ERp44) within Golgi membranes. Given that ER chaperones are known to control ER retention of certain transcription factors (e.g. ATF6), their depletion from the ER could facilitate CREB3L1 export to the Golgi and subsequent activation. Notably, the temporal coincidence of ERp44 retention, the reappearance of S1P protease in the regenerating Golgi, and the peak of CREB3L1 nuclear translocation is consistent with this model.

Finally, the temporal sequence we observe—CREB3L1 nuclear translocation peaking at ~6 h followed by induction of Golgi genes at ~10 h—supports a functional role for CREB3L1 as a regulated, rather than constitutive, transcriptional activator that promotes Golgi functional and structural maturation.

Thus, to address the reviewer's concern, we have now (i) clarified in the text that CREB3L1 is not constitutively active, (ii) explicitly discussed the current limits of mechanistic insight, and (iii) expanded the Discussion to present experimentally grounded hypotheses that can guide future work.

Dear Roman,

We have now received re-review reports from three referees, which I have included below. As you will see, you have addressed their concerns satisfactorily. Before I can finally accept the manuscript, there are some remaining editorial points which need to be addressed. In this regard would you please:

- declare employment in a biotech company in the disclosure and competing interests statement,
- rename the "Data and materials availability" section as "Data Availability"
- rename the conflict of interest statement as the "Disclosure and competing interests statement",
- remove the author credit section from the text,
- ensure figures are referred to in the main text sequentially; callouts for Fig. 9E-G, Appendix Fig. S2, Appendix Tables S1-S2 are currently missing,
- remove main and EV figure legends from the figures and place below the reference section,
- rename source file names, titles, legends and manuscript callouts as Dataset EV1-EV6 instead of Supplementary Dataset 1-6, legends should be uploaded as a separate tab/sheet in each Excel file,
- include in the appendix a title page called "Appendix for De novo Golgi biogenesis requires coordinated activation of a Golgi regulon" (remove author and affiliation list) and a table of contents with the page numbers for the listed items; nomenclature should be Appendix Figure Sx and Appendix Table Sx throughout ms and Appendix PDF; duplicated methods in the appendix should be removed,
- state the exact p values in the legends of figures 4E, H; 7B, C; 8C, E; 9B, D; EV1 B, EV2 F, EV4 F,
- indicate the statistical test used for data analysis in the legends of figures 7B, C,
- define 'n' in the legends of figures 7B, C; EV4 C,
- define the error bars in the legends of figures 7B, C; 8C, E; 9B, D; EV1 B, EV2 F, EV4 F,
- rename movies as Movie EV1-EV11 with the corresponding callouts, zipping the legends with each movie file,
- use the following section order: Title page - Abstract - Introduction - Results - Discussion - Methods - Data Availability - Acknowledgements - Disclosure and Competing Interests Statement - References - Figure Legends - Table(s) - Expanded View Figure Legends.

We include a synopsis of the paper (see http://emboj.embopress.org/). Please provide me with a general summary image (measuring 550x300 pixels), a two sentence statement and 3-5 bullet points that capture the key findings of the paper.

I am looking forward to receiving your revised manuscript.

EMBO Press is an editorially independent publishing platform for the development of EMBO scientific publications.

Best wishes,

William

William Teale, PhD
Editor
The EMBO Journal
w.teale@embojournal.org

Instructions for preparing your revised manuscript:

Read our guidance for manuscript revisions and related editorial policies: https://link.springer.com/journal/44318/submission-guidelines#cms-Revised-submissions

Please also check that the title and abstract of the manuscript are brief, yet explicit, even to non-specialists.

When assembling figures, please refer to our figure preparation guideline in order to ensure proper formatting and readability in print as well as on screen:
https://media.springernature.com/original/springer-cms/rest/v1/content/27825798/data/v1

IMPORTANT: When you send the revision we will require
- a point-by-point response to the referees' comments, with a detailed description of the changes made (as a word file).
- a word file of the manuscript text.
- individual production quality figure files (one file per figure)

- a complete author checklist
- Expanded View files (replacing Supplementary Information)
- a Reagents and Tools Table as part of the Methods section

Please remember: Digital image enhancement is acceptable practice, as long as it accurately represents the original data and conforms to community standards. If a figure has been subjected to significant electronic manipulation, this must be noted in the figure legend or in the 'Methods' section. The editors reserve the right to request original versions of figures and the original images that were used to assemble the figure.

We realize that it is difficult to revise to a specific deadline. In the interest of protecting the conceptual advance provided by the work, we recommend a revision within 3 months (6th Aug 2026). Please discuss the revision progress ahead of this time with the editor if you require more time to complete the revisions. Use the link below to submit your revision:

https://emboj.msubmit.net/cgi-bin/main.plex

------------------------------------------------

Referee #1:

The responses to my comments were satisfactory, and as far as I can tell, the comments from the other reviewers were also adequately addressed. I support publication of this compelling and innovative study.

Referee #2:

The manuscript by Forno, Abete et al. presents a highly compelling and original study identifying a Golgi "regulon" that governs transcriptional responses during de novo Golgi biogenesis. Using a previously established enzyme-mediated Golgi inactivation approach, the authors extend earlier work by exploring later recovery time points and demonstrate that fully functional Golgi stacks can reform approximately 10 hours after inactivation. Single-cell RNA sequencing reveals a substantial remodeling of the transcriptome following Golgi inactivation, including coordinated upregulation of numerous Golgi-associated genes. Through an elegant and broad palette of experimental tools (cell biology, electron microscopy, live imaging, scRNA-seq, RNAi mini-screening, FISH, bioinformatics, etc.), the authors identify CREB3L1 as a key transcriptional regulator orchestrating this Golgi-specific transcriptional program.
This is the type of study that introduces a genuinely novel conceptual advance and has the potential to become a foundational reference in the field. As such, it naturally opens many new questions that will stimulate future work.

The revised version clearly improved to my opinion in terms of clarity, rigor, and accessibility for a broad readership. Importantly, with the additional dat, improved analyses and written clarifications, I can now fully support that this manuscript is accepted for publication at the EMBO Journal.

I have only noticed a minor thing that the authors may want to correct in the final version:
- Fig. 2C: legend, it says "mean ! SD", change to +/-.

Referee #3:

The authors responded well to all my initial comments. Although no new data were added for CREB3L1, I agree with the response of the authors that addressing this experimentally is certainly beyond the scope of the current work and I think that the changes to the text are fully sufficient at the current stage. I have no further comments or suggestions.

**Referee #1:**

The responses to my comments were satisfactory, and as far as I can tell, the comments from the other reviewers were also adequately addressed. I support publication of this compelling and innovative study.

We are pleased that we were able to address all of the reviewer's concerns, and we would like to thank the reviewer for the positive evaluation of our study.

**Referee #2:**

The manuscript by Forno, Abete et al. presents a highly compelling and original study identifying a Golgi "regulon" that governs transcriptional responses during de novo Golgi biogenesis. Using a previously established enzyme-mediated Golgi inactivation approach, the authors extend earlier work by exploring later recovery time points and demonstrate that fully functional Golgi stacks can reform approximately 10 hours after inactivation. Single-cell RNA sequencing reveals a substantial remodeling of the transcriptome following Golgi inactivation, including coordinated upregulation of numerous Golgi-associated genes. Through an elegant and broad palette of experimental tools (cell biology, electron microscopy, live imaging, scRNA-seq, RNAi mini-screening, FISH, bioinformatics, etc.), the authors identify CREB3L1 as a key transcriptional regulator orchestrating this Golgi-specific transcriptional program.
This is the type of study that introduces a genuinely novel conceptual advance and has the potential to become a foundational reference in the field. As such, it naturally opens many new questions that will stimulate future work.

The revised version clearly improved to my opinion in terms of clarity, rigor, and accessibility for a broad readership. Importantly, with the additional dat, improved analyses and written clarifications, I can now fully support that this manuscript is accepted for publication at the EMBO Journal.

I have only noticed a minor thing that the authors may want to correct in the final version:
- Fig. 2C: legend, it says "mean ! SD", change to +/-.

We thank the reviewer for very positive feedback. Small correction was done according to the reviewer's indications.

**Referee #3:**

The authors responded well to all my initial comments. Although no new data were added for CREB3L1, I agree with the response of the authors that addressing this experimentally is certainly beyond the scope of the current work and I think that the changes to the text are fully sufficient at the current stage. I have no further comments or suggestions.

We are pleased that we were able to respond to the reviewer's comments effectively. We will definitely address the reviewer's question regarding the mechanisms of CREB3L1 activation in future studies.

## 2nd Revision - Editorial Decision 17th May 2026

Dear Roman, dear Franck,

I am pleased to inform you that your manuscript has been accepted for publication in the EMBO Journal.

Congratulations to both of you and to all involved!

Your manuscript will be processed for publication by EMBO Press. It will be copy edited and you will receive page proofs prior to publication. Please note that you will be contacted by Springer Nature Author Services to complete licensing and payment information.

You may qualify for financial assistance for your publication charges - either via a Springer Nature fully open access agreement or an EMBO initiative. Check your eligibility: https://link.springer.com/journal/44318/how-to-publish-with-us

Should you be planning a Press Release on your article, please get in contact with embo_production@springernature.com as early as possible in order to coordinate publication and release dates.

If you have any questions, please do not hesitate to contact the Editorial Office. Thank you for your contribution to The EMBO Journal.

Best wishes,

William

William Teale, PhD
Editor
The EMBO Journal
w.teale@embojournal.org

------------------------------------------------

*****
Please note that it is The EMBO Journal policy for the transcript of the editorial process (containing referee reports and your response letters) to be published as an online supplement to each paper. If you should prefer removal of any referee-only figures included in the point-by-point response(s), e.g. because they may still be used for future publication or because they have been reproduced from published work by others, please do let us know immediately via response email.
More information is available here: https://link.springer.com/partners/embo-press/editorial-policies#Peer%20review

** Click here to be directed to your login page: https://emboj.msubmit.net

## EMBO Press Author Checklist

| | |
|---|---|
| Corresponding Author Name: Roman Polishchuk | |
| Journal Submitted to: The EMBO Journal | |
| Manuscript Number: 2025-123060 | |

**USEFUL LINKS FOR COMPLETING THIS FORM**
The EMBO Journal - Author Guidelines
EMBO Reports - Author Guidelines
Molecular Systems Biology - Author Guidelines
EMBO Molecular Medicine - Author Guidelines

**Reporting Checklist for Life Science Articles (updated January**
This checklist is adapted from Materials Design Analysis Reporting (MDAR) Checklist for Authors. MDAR establishes a minimum set of requirements in transparent reporting in the life sciences (see Statement of Task: 10.31222/osf.io/9sm4x). Please follow the journal's guidelines in preparing your
**Please note that a copy of this checklist will be published alongside your article.**

**Abridged guidelines for figures**
**1. Data**
The data shown in figures should satisfy the following conditions:
- the data were obtained and processed according to the field's best practice and are presented to reflect the results of the experiments in an accurate and unbiased manner.
- ideally, figure panels should include only measurements that are directly comparable to each other and obtained with the same assay.
- plots include clearly labeled error bars for independent experiments and sample sizes. Unless justified, error bars should not be shown for technical
- if n<5, the individual data points from each experiment should be plotted. Any statistical test employed should be justified.
- Source Data should be included to report the data underlying figures according to the guidelines set out in the authorship guidelines on Data

**2. Captions**
Each figure caption should contain the following information, for each panel where they are relevant:
- a specification of the experimental system investigated (eg cell line, species name).
- the assay(s) and method(s) used to carry out the reported observations and measurements.
- an explicit mention of the biological and chemical entity(ies) that are being measured.
- an explicit mention of the biological and chemical entity(ies) that are altered/varied/perturbed in a controlled manner.
- the exact sample size (n) for each experimental group/condition, given as a number, not a range;
- a description of the sample collection allowing the reader to understand whether the samples represent technical or biological replicates (including how many animals, litters, cultures, etc.).
- a statement of how many times the experiment shown was independently replicated in the laboratory.
- definitions of statistical methods and measures:
  - common tests, such as t-test (please specify whether paired vs. unpaired), simple χ2 tests, Wilcoxon and Mann-Whitney tests, can be unambiguously identified by name only, but more complex techniques should be described in the methods section;
  - are tests one-sided or two-sided?
  - are there adjustments for multiple comparisons?
  - exact statistical test results, e.g., P values = x but not P values < x;
  - definition of 'center values' as median or average;
  - definition of error bars as s.d. or s.e.m.

**Please complete ALL of the questions below.**
**Select "Not Applicable" only when the requested information is not relevant for your study.**

### Materials

| Newly Created Materials | Information included in the manuscript? | In which section is the information available? (Reagents and Tools Table, Materials and Methods, Figures, Data Availability Section) |
|---|---|---|
| New materials and reagents need to be available; do any restrictions apply? | Yes | Data Availability Section |

| Antibodies | Information included in the manuscript? | In which section is the information available? (Reagents and Tools Table, Materials and Methods, Figures, Data Availability Section) |
|---|---|---|
| For **antibodies** provide the following information:<br>- Commercial antibodies: RRID (if possible) or supplier name, catalogue number and or/clone number<br>- Non-commercial: RRID or citation | Yes | Reagents and Tools Table, Materials and Methods |

| DNA and RNA sequences | Information included in the manuscript? | In which section is the information available? (Reagents and Tools Table, Materials and Methods, Figures, Data Availability Section) |
|---|---|---|
| **Short novel DNA or RNA including primers, probes**: provide the sequences. | Yes | Apendix Tables S1, 2 |

| Cell materials | Information included in the manuscript? | In which section is the information available? (Reagents and Tools Table, Materials and Methods, Figures, Data Availability Section) |
|---|---|---|
| **Cell lines:** Provide species information, strain. Provide accession number in repository **OR** supplier name, catalog number, clone number, and/**OR** RRID. | Yes | Reagents and Tools Table, Materials and Methods |
| **Primary cultures:** Provide species, strain, sex of origin, genetic modification status. | Not Applicable | |
| Report if the cell lines were recently **authenticated** (e.g., by STR profiling) and tested for mycoplasma contamination. | Yes | Matrials and Mehods |

| Experimental animals | Information included in the manuscript? | In which section is the information available? (Reagents and Tools Table, Materials and Methods, Figures, Data Availability Section) |
|---|---|---|
| **Laboratory animals or Model organisms:** Provide species, strain, sex, age, genetic modification status. Provide accession number in repository **OR** supplier name, catalog number, clone number, **OR** RRID. | Not Applicable | |
| **Animal observed in or captured from the field:** Provide species, sex, and age where possible. | Not Applicable | |
| Please detail **housing and husbandry conditions**. | Not Applicable | |

| Plants and microbes | Information included in the manuscript? | In which section is the information available? (Reagents and Tools Table, Materials and Methods, Figures, Data Availability Section) |
|---|---|---|
| **Plants:** provide species and strain, ecotype and cultivar where relevant, unique accession number if available, and source (including location for collected wild specimens). | Not Applicable | |
| **Microbes:** provide species and strain, unique accession number if available, and source. | Not Applicable | |

| Human research participants | Information included in the manuscript? | In which section is the information available? (Reagents and Tools Table, Materials and Methods, Figures, Data Availability Section) |
|---|---|---|
| If collected and within the bounds of privacy constraints report on age, sex and gender or ethnicity for all study participants. | Not Applicable | |

| Core facilities | Information included in the manuscript? | In which section is the information available? (Reagents and Tools Table, Materials and Methods, Figures, Data Availability Section) |
|---|---|---|
| If your work benefited from core facilities, was their service mentioned in the acknowledgments section? | Yes | Acknowledgements section |

### Design

| Study protocol | Information included in the manuscript? | In which section is the information available? (Reagents and Tools Table, Materials and Methods, Figures, Data Availability Section) |
|---|---|---|
| If study protocol has been **pre-registered, provide DOI in the manuscript**. For clinical trials, provide the trial registration number **OR** cite DOI. | Not Applicable | |
| Report the **clinical trial registration number** (at ClinicalTrials.gov or equivalent), where applicable. | Not Applicable | |

| Laboratory protocol | Information included in the manuscript? | In which section is the information available? (Reagents and Tools Table, Materials and Methods, Figures, Data Availability Section) |
|---|---|---|
| Provide DOI OR other citation details if **external detailed step-by-step protocols** are available. | Not Applicable | Spet by step protocols are available in Appendix (Materials and Methods section) |

| Experimental study design and statistics | Information included in the manuscript? | In which section is the information available? (Reagents and Tools Table, Materials and Methods, Figures, Data Availability Section) |
|---|---|---|
| Include a statement about **sample size** estimate even if no statistical methods were used. | Yes | Materials and Methods |
| Were any steps taken to minimize the effects of subjective bias when allocating animals/samples to treatment (e.g. **randomization procedure**)? If yes, have they been described? | Not Applicable | |
| Include a statement about **blinding** even if no blinding was done. | Yes | Materials and Methods |
| Describe **inclusion/exclusion criteria** if samples or animals were excluded from the analysis. Were the criteria pre-established?<br><br>If sample or data points were omitted from analysis, report if this was due to attrition or intentional exclusion and provide justification. | Not Applicable | |
| For every figure, are **statistical tests** justified as appropriate? Do the data meet the assumptions of the tests (e.g., normal distribution)? Describe any methods used to assess it. Is there an estimate of variation within each group of data? Is the variance similar between the groups that are being statistically compared? | Yes | Materials and Methods, Figures |

| Sample definition and in-laboratory replication | Information included in the manuscript? | In which section is the information available? (Reagents and Tools Table, Materials and Methods, Figures, Data Availability Section) |
|---|---|---|
| In the figure legends: state number of times the experiment was **replicated** in laboratory. | Yes | Figures, Materials and Methods (Statistics) |
| In the figure legends: define whether data describe **technical or biological replicates**. | Yes | Figures, , Materials and Methods (Statistics) |

## Ethics

| Ethics | Information included in the manuscript? | In which section is the information available? (Reagents and Tools Table, Materials and Methods, Figures, Data Availability Section) |
|---|---|---|
| Studies involving **human participants**: State details of **authority granting ethics approval** (IRB or equivalent committee(s), provide reference number for approval. | Not Applicable | |
| Studies involving **human participants**: Include a statement confirming that **informed consent** was obtained from all subjects and that the experiments conformed to the principles set out in the WMA Declaration of Helsinki and the Department of Health and Human Services Belmont Report. | Not Applicable | |
| Studies involving **human participants:** For publication of **patient photos**, include a statement confirming that consent to publish was obtained. | Not Applicable | |
| Studies involving experimental **animals**: State details of **authority granting ethics approval** (IRB or equivalent committee(s), provide reference number for approval. Include a statement of compliance with ethical regulations. | Not Applicable | |
| Studies involving **specimen and field samples:** State if relevant **permits** obtained, provide details of authority approving study; if none were required, explain why. | Not Applicable | |

| Dual Use Research of Concern (DURC) | Information included in the manuscript? | In which section is the information available? (Reagents and Tools Table, Materials and Methods, Figures, Data Availability Section) |
|---|---|---|
| Could your study fall under dual use research restrictions? Please check biosecurity documents and list of **select agents and toxins** (CDC): https://www.selectagents.gov/sat/list.htm | Not Applicable | |
| If you used a select agent, is the security level of the lab appropriate and reported in the manuscript? | Not Applicable | |
| If a study is subject to dual use research of concern regulations, is the name of the **authority granting approval and reference number** for the regulatory approval provided in the manuscript? | Not Applicable | |

## Reporting

The MDAR framework recommends adoption of discipline-specific guidelines, established and endorsed through community initiatives. Journals have their own policy about requiring specific guidelines and recommendations to complement MDAR.

| Adherence to community standards | Information included in the manuscript? | In which section is the information available? (Reagents and Tools Table, Materials and Methods, Figures, Data Availability Section) |
|---|---|---|
| State if relevant guidelines or checklists (e.g., **ICMJE, MIBBI, ARRIVE, PRISMA**) have been followed or provided. | Yes | Authors contribution section |
| For **tumor marker prognostic studies,** we recommend that you follow the **REMARK** reporting guidelines (see link list at top right). See author guidelines, under 'Reporting Guidelines'. Please confirm you have followed these guidelines. | Not Applicable | |
| For **phase II and III randomized controlled trials**, please refer to the **CONSORT** flow diagram (see link list at top right) and submit the CONSORT checklist (see link list at top right) with your submission. See author guidelines, under 'Reporting Guidelines'. Please confirm you have submitted this list. | Not Applicable | |

## Data Availability

| Data availability | Information included in the manuscript? | In which section is the information available? (Reagents and Tools Table, Materials and Methods, Figures, Data Availability Section) |
|---|---|---|
| Have **primary datasets** been deposited according to the journal's guidelines (see 'Data Deposition' section) and the respective accession numbers provided in the Data Availability Section? | Yes | Data Availability Section |
| Were **human clinical and genomic datasets** deposited in a public access-controlled repository in accordance to ethical obligations to the patients and to the applicable consent agreement? | Not Applicable | |
| Are **computational models** that are central and integral to a study available without restrictions in a machine-readable form? Were the relevant accession numbers or links provided? | Not Applicable | |
| If publicly available data were reused, provide the respective **data citations in the reference list**. | Not Applicable | |
